# Styrylquinazoline derivatives as ABL inhibitors selective for different DFG orientations

**DOI:** 10.1080/14756366.2023.2201410

**Published:** 2023-04-18

**Authors:** Katarzyna Malarz, Jacek Mularski, Marcin Pacholczyk, Robert Musiol

**Affiliations:** a Institute of Physics, University of Silesia in Katowice, Chorzów, Poland; b Institute of Chemistry, University of Silesia in Katowice, Chorzów, Poland; c Department of Systems Biology and Engineering, Silesian University of Technology, Gliwice, Poland

**Keywords:** Quinazoline, kinase inhibitor, tyrosine kinase, ABL kinase, leukaemia, anticancer activity

## Abstract

Among tyrosine kinase inhibitors, quinazoline-based compounds represent a large and well-known group of multi-target agents. Our previous studies have shown interesting kinases inhibition activity for a series of 4-aminostyrylquinazolines based on the CP-31398 scaffold. Here, we synthesised a new series of styrylquinazolines with a thioaryl moiety in the C4 position and evaluated in detail their biological activity. Our results showed high inhibition potential against non-receptor tyrosine kinases for several compounds. Molecular docking studies showed differential binding to the DFG conformational states of ABL kinase for two derivatives. The compounds showed sub-micromolar activity against leukaemia. Finally, in-depth cellular studies revealed the full landscape of the mechanism of action of the most active compounds. We conclude that S^4^-substituted styrylquinazolines can be considered as a promising scaffold for the development of multi-kinase inhibitors targeting a desired binding mode to kinases as effective anticancer drugs.

## Introduction

In 2001, the first kinase inhibitor targeting the ABL kinase, imatinib, was launched, and was viewed as a new wonder weapon. This triggered the development of a series of new small molecules specifically inhibiting oncogenic kinases. Shortly thereafter, it was recognised that cancer had a counter-magic, namely, mutation-dependent resistance, which turned out to be a problem even in drug naïve patients. Among mutation responsible for this resistance, the single point mutation T315I, which is often called the “gatekeeper”, is particularly difficult to target because it is resistant to subsequently introduced several drugs, regardless of their superior activity or their selectivity to imatinib. Later, ponatinib was developed, a multi-kinase inhibitor that was able to overcome this mutation. Nevertheless, therapeutic options were far from satisfactory as several other resistance-mediating mutations were identified. Moreover, some of those mutations appeared to have an additional gain-of-function effect that stabilised the protein in more active conformations^1^.

**Figure 1.**
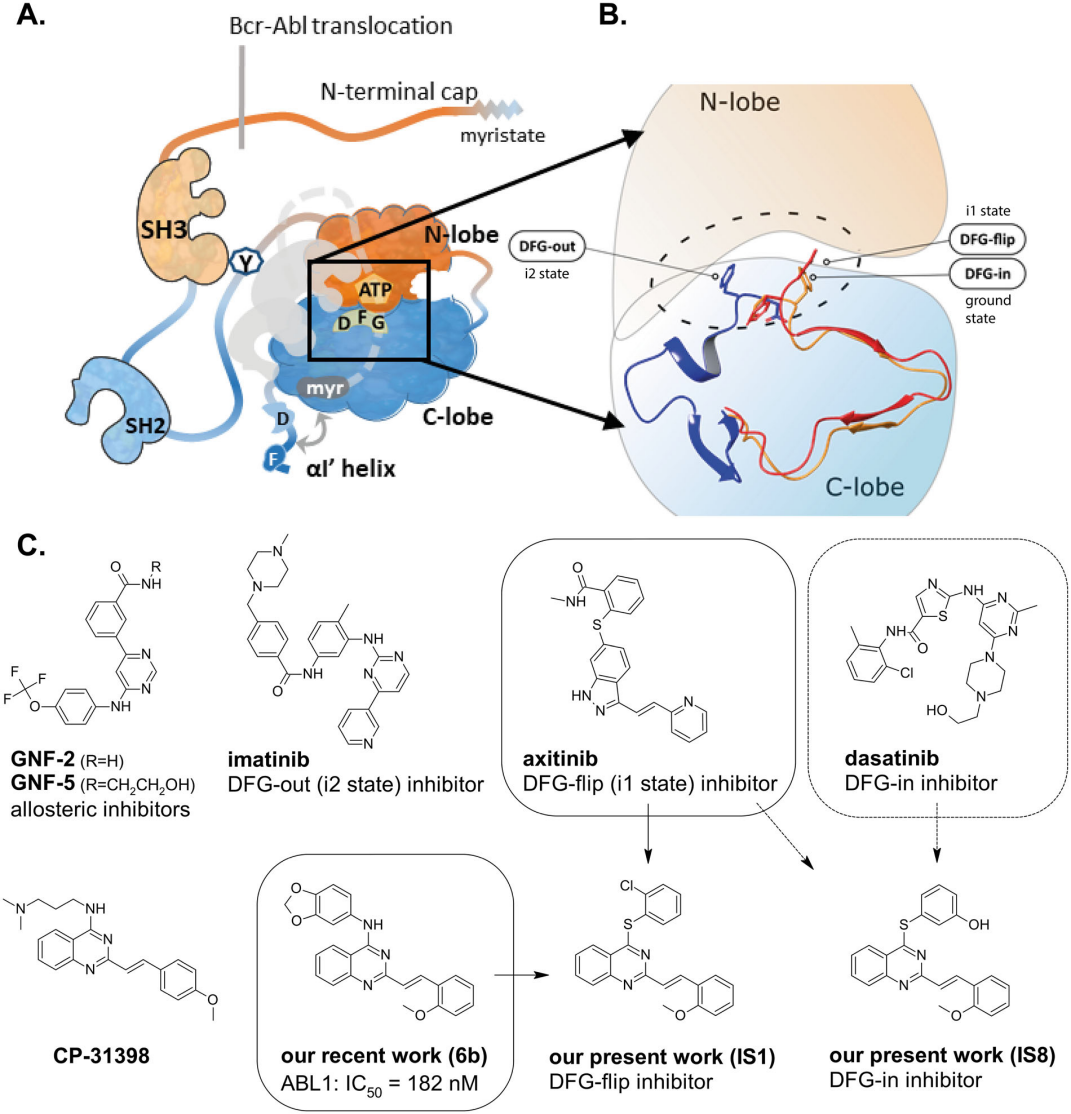
Schematic representation of the ABL kinase domains with the binding sites for competitive and allosteric inhibitors that are mentioned in the text. Tyr245 (Y) is the site of phosphorylation exposed in the open active conformation. The closed conformation of the apo enzyme is shaded with the cap clamping to the myristoyl pocket. The αl’ helix, which is composed of the DNA binding domain and the F-actin binding domain (D and F) closed after binding to the myristoyl pocket (A). Differences between the DFG in/out conformations (B). Approved ABL inhibitors (GNF-2/5, imatinib, axitinib, dasatinib) bind to different conformation states of the kinase. IS1 and IS8 are the thio-analogues of compound 6b, synthesised in our previous work^12^. Approved ABL inhibitors and CP-31398 are used as reference compounds in this work (C).

In generally, there are four main regions of conformational flexibility in the ABL kinase domain: the A-loop, the P-loop, the C-helix and the relative position of the N-terminal lobe with respect to the C-terminal lobe. These conformational differences are not only important for the normal activity cycle of the enzyme, but also lead to changes in the properties of the inhibitor-binding site and can be exploited to increase selectivity while optimising affinity. The c-ABL kinase in its normal apo form is auto-inhibited by its SH2 and SH3 domains, located near the main part of the enzyme, and by the N-terminal cap, which clasps the entire structure, stabilising the closed form^2^^,^^3^. Conversely, release of the SH2 and SH3 domains and unbounding the αl’ helix are required to activate the enzyme^4^. This opens the sites for trans-protein interactions and the residues for phosphorylation that allow normal activity. This also explains why the enzyme is constitutively active in the BCR-ABL mutation. Chromosome fusion results in the substitution of the N-terminal cap and the myristoylated end with a non-functional BCR portion (Figure 1(A)).

An intriguing alternative to these classical inhibitors, whose target is the ATP-binding site of the enzyme, was found with the observation that conformational changes of the distal SH2 and SH3 domains are required to achieve an inactive form. This spatial reorganisation is triggered by the binding of myristic acid to the pocket in the N-terminal domain of the enzyme^2^. Later, the search for allosteric myristoyl pocket inhibitors revealed that GNF-2 and GNF-5 (Figure 1(C)) are the most active compounds at sub-micromolar concentration. Their discovery and development was described in the excellent work by Gray et al.^5^. To date, several compounds have been identified that are capable of binding to this pocket^6^. These findings led to the development of asciminib (also known as ABL001), which is currently being tested in a clinical trial in patients with Acute Myeloid Leukaemia (AML) in combination with other classical inhibitors^7^.

The activation loop (A-loop) plays a key role in regulating the catalytic activity of protein kinases. This structural element of the ABL kinase can adopt three states (see Figure 1(B)). Two of them resemble similar the open form, and distinct unfolded one. The activation loop contains three conserved Asp-Phe-Gly residues. They adopt specific orientation within the catalytic cavity following rotation of the A-loop. One orientation could be defined as active state (DFG-in), where the carboxyl group of Asp is catalytically oriented towards the binding pocket. The other two orientations are defined as inactive (inactive-1 and inactive-2 denoted as i1 and i2, respectively). They are in dynamic equilibrium, and the i1 state is in equilibrium with the active state. These differences have a great potential for drug design. For example, kinases such as c-Src, which are presumably unable to adopt the DFG-out conformation, do not bind imatinib strongly^8^. The DFG motif has a conformation similar to that observed in active kinases. As a result, imatinib or its analogues bind to c-Src with a different configuration and consequently a lower affinity^9^. The DFG-out conformation was also observed with other kinases, explaining the high selectivity of imatinib^10^. It should also be noted here that the ability of a kinase to adopt the DFG-out conformation is necessary, but not sufficient, for binding to imatinib. The P-loop (phosphate-binding or glycine-rich loop) contributes to selectivity because, as mentioned earlier, this loop adopts an inactive conformation in a complex with imatinib that allows extensive contacts with the inhibitor. This conformation allows Tyr253 of the P-loop to form a face-to-edge aromatic interaction with the pyrimidine group of imatinib. Recently, Hanson et al. published an interesting discussion on the causes of the propensity of kinases to inhibitors that shed some light on this pattern^11^. The conformational stability, which depends on salt bridges, and the appearance of hydrophobic pockets provide opportunities for different molecules to bind.

**Figure 2.**
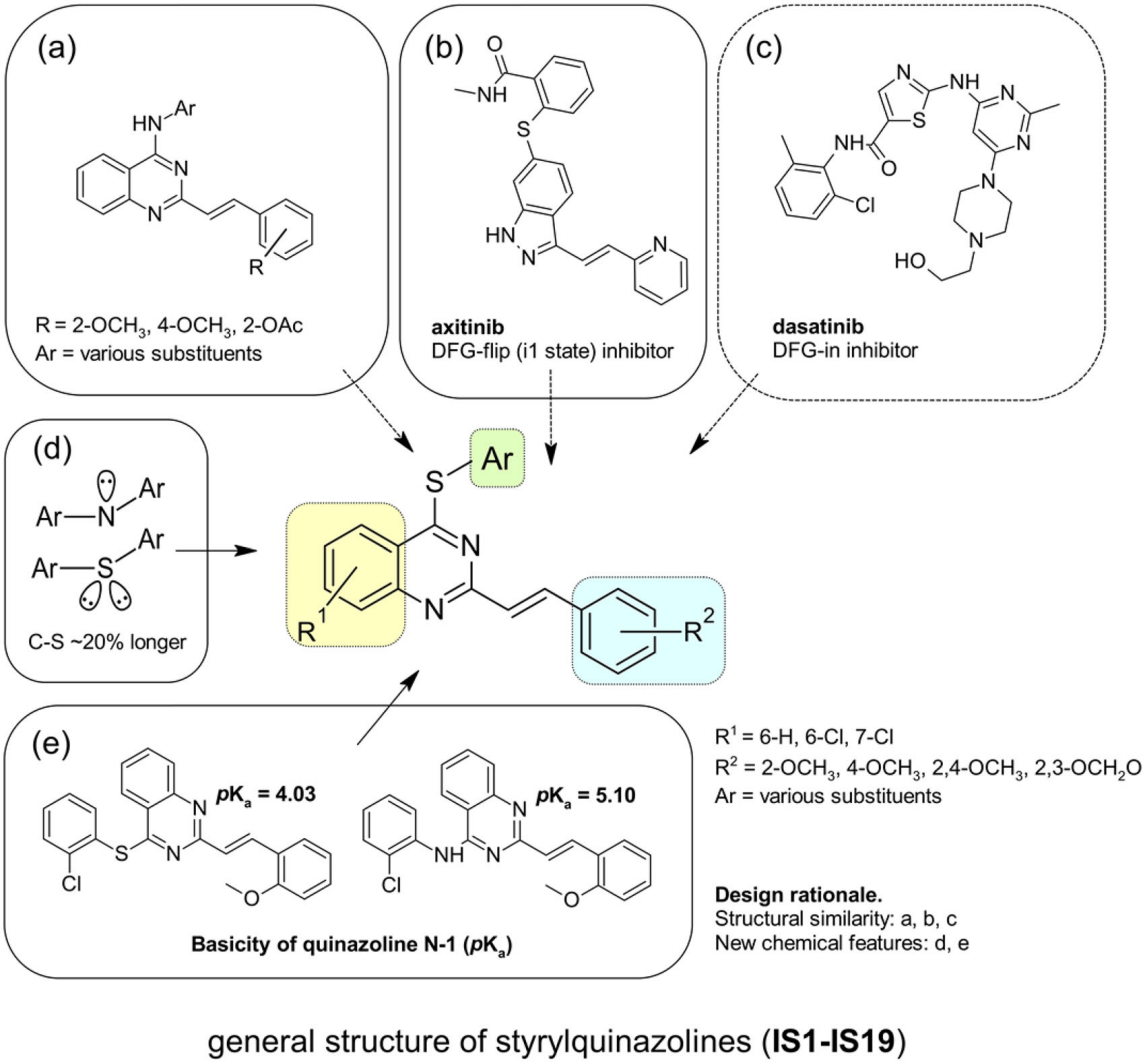
The rationalisation of the design of novel S^4^-substituted styrylquinazolines. An approach based on the similarity of structures (a-c) and chemical features (d-e).

Non-classical inhibition, e.g. by the myristoyl pocket or DFG orientation, tends to restore the closed inactive conformation, which is triggered and further stabilised by the myristoyl pocket capped by the αI’ helix^13^. Shrinkage of the flexible domains also leads to closure of the phosphorylation site. Oligomerisation (especially in BCR-ABL) is responsible for the partial phosphorylation of protein-imatinib complexes^14^. This explains the synergistic interactions between GNF-2/5 and imatinib, but not all competitive inhibitors^15^. A similar mutual enhancement of inhibitory effect was observed for asciminib in combination with ponatinib^16^. Particularly interesting is the observation that combinations of non-classical and ATP-competitive inhibitors can restore each other’s activity against resistant mutations^15^^,^^17^. We have previously investigated a series of quinolines^18–21^ and 4-aminostyrylquinazoline derivatives, e.g. 6b in Figure 1(C), which are structurally related to CP-31398 (chemical structure is shown in Figure 1(C))^12^. These compounds exhibited good anticancer activity as multi-targeted kinase inhibitors. In our current work we have focussed on this styrylquinazoline scaffold in a search for new structural features that could provide higher affinity to the protein. It is important to realise that 4-aminoarylstyrylquinazoline although similar to axitinib may adopt conformation resembling to dasatinib as presented in Figure 2. Recently, we have investigated a sulphur containing analogues - a series of 2-[(*E*)-2-phenylethenyl]quinazolin-4-yl benzenesulfonates. Interestingly, we found that these analogues acted as competitive ATP inhibitors depending on the pattern of their substituents^22^. Based on these results, in our current study we changed the linker to sulfanyl group. Conversion of a C-SO_2_R group into a sulphide is a strategy to increase biochemical stability while maintaining good bioavailability. The linker build on thioether is longer that amine counterpart and offer more electron-rich scaffold that may be beneficial for ATP –binding proteins (Figure 2(D)). Moreover, according to Lewis et al.^23^ thioethers provide stronger interactions with protein aminoacid residues including S-aromatic aminoacids and methionine. This is of particular interest because the M315T mutation, in which threonine is replaced by methionine, is frequently reported as a resistance factor for imatinib. Therefore, we designed several thio-analogues of styrylquinazoline derivatives with respect to previously described benzodioxole- and phenyl- substituted compounds. These compounds were tested for their activity as non-receptor tyrosine kinase inhibitors, including the ABL and Src family. Different behaviours and binding modes were postulated for the styrylquinazoline derivatives tested. Further biological studies revealed a good antitumor activity for the three derivatives. Because a simple analysis and linking of inhibitory activity against the enzymes to an antiproliferative activity on cancer cell lines might not be sufficient to determine the therapeutic potential of the compounds, further detailed studies on the molecular mechanism of action were performed.

## Results and discussion

### Synthesis of styrylquinazolines

**Scheme 1.**
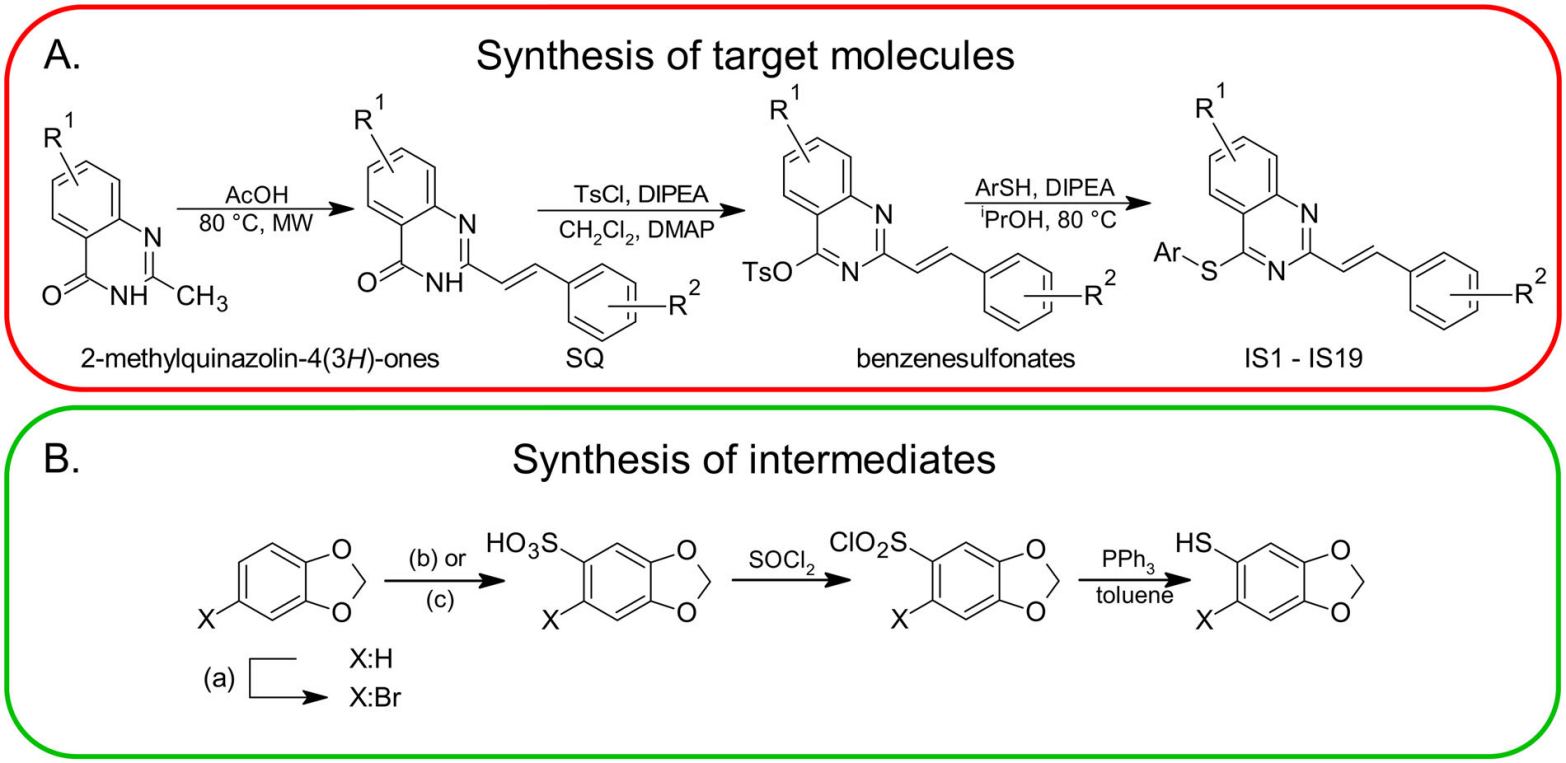
The general multi-stage chemical synthesis procedure of the target molecules. R^1^: 6-H, 6-Cl, 7-Cl; R^2^:2-OCH_3_, 4-OCH_3_, 2,4-OCH_3_, 2,3-OCH_2_O; Ar: various substituents (A). The synthetic procedure that was used to obtain arylthiols with a methylenedioxy moiety. (a) NBS, CH_3_CN; (b) X = H: Ac_2_O/H_2_SO_4_, EtOAc; (c) X = Br: HOSO_2_Cl, CH_2_Cl_2_ (B).

The synthesis routes that were used to obtain the target compounds are illustrated in Scheme 1(A). According to this scheme, the final thio-analogues were synthesised in a multi-stage procedure, which is described in detail in the experimental section. Two intermediates – *2H*-1,3-benzodioxole-5-thiol and 6-bromo-*2H*-1,3-benzodioxole-5-thiol – were obtained according by standard procedures (Scheme 1(B))^24–27^.

The structure of all synthesised compounds was confirmed using ^1^H and ^13^C NMR spectra and high-resolution mass spectrometry (HRMS) (all spectra are included in the Supplementary Data).

### Inhibition of tyrosine kinases

All newly synthesised derivatives were screened for their inhibitory potential towards eight non-receptor tyrosine kinases, such as ABL, BTK, BRK and Src family kinases, such as CSK, Lyn, Lck, Fyn and Src. The inhibitory effect of the derivatives was tested at a single-dose concentration (0.5 µM) after 1h incubation with the kinase and its peptide substrate in the presence of ATP. The references in our assay were both single-target and multi-target inhibitors, including imatinib, axitinib, dasatinib, GNF-2 and styrylquinazoline CP-31398 (chemical structures are shown in Figure 1(C)). The results are shown in Table 1 as the percentage of kinase inhibition. To facilitate the analysis of the structure and enzymatic activity, all tested compounds were divided into four subgroups based on the main structural scaffolds (the general structure of each group is shown in the second column of Table 1). Series A, B, and C were subdivided according to the S^4^-aryl substituent, while D represents the different styryl groups at the C^2^-quinazoline position.

**Table 1.** Inhibitory activity of the styrylquinazoline compounds tested against a panel of non-receptor tyrosine kinases.

Compound	General structure	Ar	Inhibition of tyrosine kinase activity [%]^a^							
			ABL	BTK	BRK	CSK	Fyn A	Lck	Lyn B	Src
Series A										
IS1	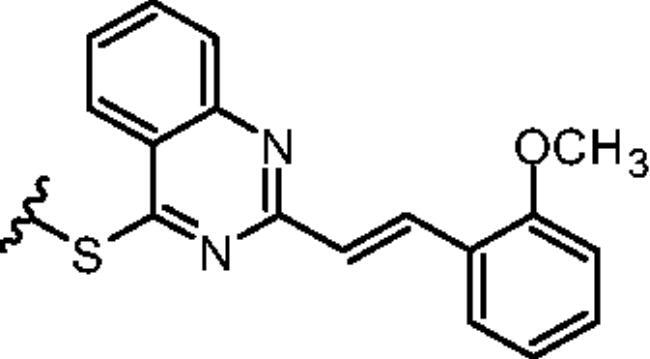	2-Cl-C_6_H_4_	**90.81 ± 5.71**	**89.02 ± 8.34**	**51.27 ± 9.04**	**51.13 ± 5.72**	**90.41 ± 9.90**	**79.08 ± 3.51**	**67.08 ± 5.10**	**79.52 ± 9.56**
IS2		3-Cl-C_6_H_4_	25.21 ± 3.69	NT	NT	**62.13 ± 4.45**	**52.32 ± 7.04**	**73.92 ± 9.72**	38.41 ± 8.58	**58.56 ± 4.80**
IS3		2,5-Cl-C_6_H_4_	4.02 ± 3.58	31.62 ± 2.38	0	35.00 ± 1.11	**59.95 ± 2.85**	13.36 ± 5.58	26.23 ± 1.55	0
IS4		2-Br-C_6_H_4_	29.84 ± 0.95	45.87 ± 7.54	0	17.43 ± 6.31	34.12 ± 2.53	0	3.39 ± 0.47	18.50 ± 1.07
IS5		2-F-C_6_H_4_	18.47 ± 1.59	NT	NT	23.14 ± 5.24	3.77 ± 1.21	37.02 ± 7.87	7.67 ± 3.28	28.20 ± 5.41
IS6		2-CF_3_-C_6_H_4_	1.18 ± 0.17	NT	NT	0	26.12 ± 8.39	0	1.39 ± 0.41	43.82 ± 9.07
IS7		3-CH_3_O-C_6_H_4_	45.94 ± 2.63	**53.46 ± 1.33**	0	39.33 ± 4.74	25.68 ± 2.29	0	0	8.17 ± 1.71
IS8		3-OH-C_6_H_4_	**54.14 ± 3.45**	19.12 ± 1.23	15.79 ± 2.25	0	0	0	0	5.12 ± 0.96
IS9		4-CH_3_S-C_6_H_4_	35.91 ± 7.23	28.66 ± 2.94	19.27 ± 4.65	39.19 ± 6.47	**52.04 ± 3.43**	**60.73 ± 5.10**	**57.44 ± 1.76**	**54.23 ± 3.07**
IS10		1,3-benzodioxol-5-yl	14.21 ± 3.91	15.93 ± 3.66	25.03 ± 8.51	0	21.49 ± 5.71	28.08 ± 5.74	0	0
IS11		6-Br-1,3-benzodioxol-5-yl	18.41 ± 2.37	2.87 ± 0.67	0	**50.68 ± 5.60**	19.15 ± 3.57	0	0.86 ± 0.23	40.91 ± 1.77
Series B										
IS12	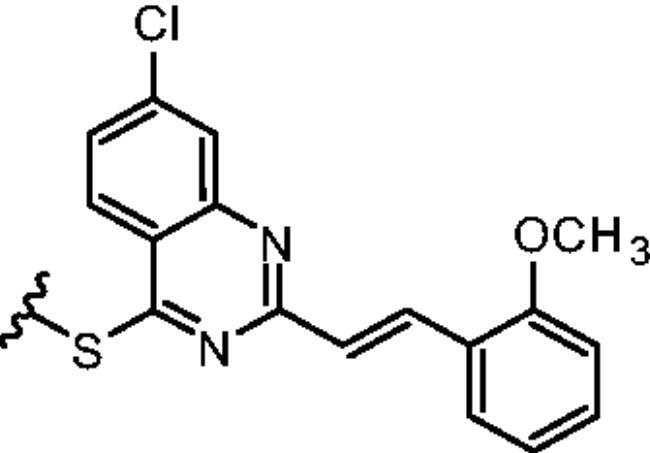	2-Cl-C_6_H_4_	0	15.36 ± 4.19	0	0	0	0	0	0
IS13		3-CH_3_O-C_6_H_4_	0	0	0	0	0	0	0	0
IS14		pyridin-2-yl	7.64 ± 0.70	NT	NT	0	15.97 ± 7.94	14.82 ± 6.02	0	40.45 ± 9.17
IS15		6-Br-1,3-benzodioxol-5-yl	41.81 ± 4.38	39.27 ± 2.69	27.24 ± 1.31	33.08 ± 1.19	**57.46 ± 4.04**	**51.49 ± 4.55**	32.32 ± 2.51	**53.36 ± 3.13**
Series C										
IS16	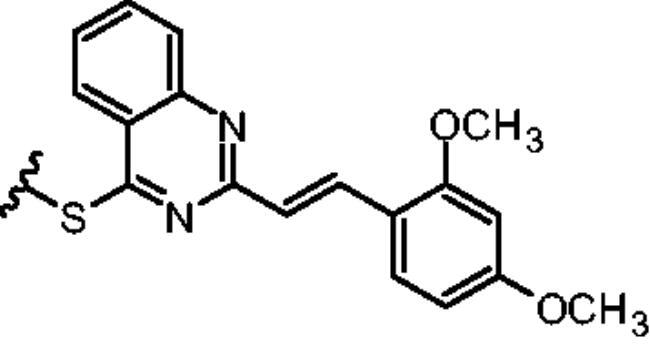	2-Cl-C_6_H_4_	35.65 ± 1.50	13.07 ± 2.69	0	15.47 ± 2.88	**50.12 ± 3.49**	48.10 ± 6.55	0	0
IS17		6-Br-1,3-benzodioxol-5-yl	11.93 ± 2.86	9.58 ± 0.62	0	7.77 ± 3.16	19.79 ± 3.51	0	0	21.00 ± 1.82

Series D										
Compound	General structure	R^2^	ABL	BTK	BRK	CSK	Fyn A	Lck	Lyn B	Src
IS18	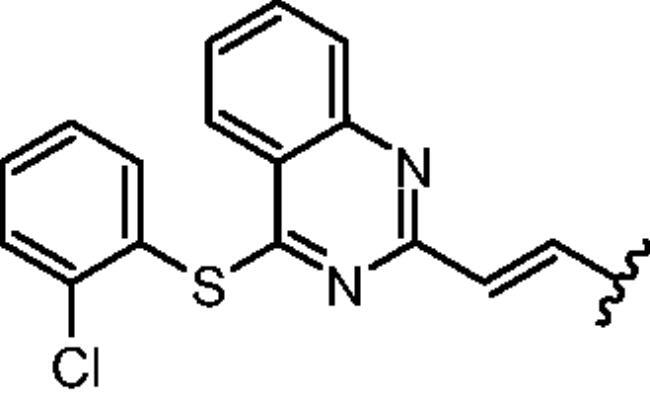	4-CH_3_O-C_6_H_4_	11.72 ± 4.03	NT	NT	0	17.26 ± 4.47	**66.49 ± 7.49**	13.84 ± 1.00	26.10 ± 6.03
IS19		1,3-benzodioxol-4-yl	8.68 ± 2.51	NT	NT	19.43 ± 7.94	24.08 ± 7.48	**54.27 ± 9.92**	9.12 ± 2.79	47.04 ± 5.70

References										
CP-31398			10.02 ± 2.69	36.83 ± 3.66	34.70 ± 7.83	0.98 ± 0.41	30.65 ± 7.83	27.87 ± 4.31	47.10 ± 4.71	15.73 ± 6.38
Imatinib			**77.17 ± 3.85**	0	0	0	0	0	0	0
Axitinib			**56.63 ± 2.41**	0	0	11.37 ± 3.89	43.81 ± 2.96	10.08 ± 4.43	38.96 ± 9.95	10.80 ± 1.95
GNF-2			**69.57 ± 7.94**	0	0	0	0	0	0	0
Dasatinib			**89.30 ± 1.01**	**97.05 ± 2.88**	**91.66 ± 1.93**	**94.25 ± 1.28**	**92.27 ± 2.55**	**97.12 ± 3.57**	**97.55 ± 1.49**	**98.16 ± 5.34**

^a^The percentage of inhibition at a 0.5 μM concentration. Compounds with an inhibition percentage above 50% are bolded. The results are expressed as mean ± *SD* from four independent experiments. NT: not tested.

Among the 4-sulphur substituted quinazolines tested, compound IS1 had the highest inhibitory potential against a panel of non-receptor tyrosine kinases. The compound belongs to series A (Table 1), which carries a 2-methoxystyryl moiety (series A). First of all, for IS1 at 0.5 µM, a 90.81% inhibition of ABL kinase activity was registered. Similar levels of IS1 inhibition were also observed for the BTK and Fyn kinases. For the reference inhibitors, imatinib, axitinib, GNF-2 and dasatinib, we recorded a 77%, 57%, 70% and 89% inhibition of the ABL kinase activity, respectively. Interestingly, in addition to IS1 (Ar = 2-Cl-C_6_H_4_) we observed good inhibitory activity against ABL only with two analogues, IS7 (Ar = 3-MeO-C_6_H_4_) and IS8 (Ar = 3-OH-C_6_H_4_), both with a meta-oriented substituent distal to the sulphur atom attached to the quinazoline core. In the case of IS8, the level of the ABL inhibition reached 54%. On the other hand, we tried a combination of the physico-chemical properties of the above substituent, resulting in IS10 (Ar = 1,3-benzodioxol-5-yl) and IS11 (Ar = 6-Br-1,3-benzodioxol-5-yl), but without any improvement in the inhibitory effect on ABL kinase. However, modification of the 7-Cl-substituted quinazoline ring (IS15, series B), resulted in moderate (41%) inhibition of ABL. All other compounds tested containing a benzodioxole moiety were inactive (IS11, IS17 and IS19). The results for compounds containing benzodioxole are all the more surprising because many reports have indicated their good efficacy in inhibiting many eukaryotic kinases^28^^,^^29^.

Interestingly, IS1 had a good inhibitory effect on Lck and Src proteins, with a degree of inhibition of 79%. Its analogue, IS2 (Ar = 3-Cl-C_6_H_4_), exhibited a similarly strong inhibitory activity on Lck kinase (almost 74% inhibition) and was even more effective against CSK kinase (compared with all other compounds). In addition, the IS2 derivative achieved a satisfactory inhibitory effect on Fyn and Src kinase (52% and 58%, respectively). Strikingly, IS9, IS15, and IS16 had a good inhibitory activity on two Src family kinases: Fyn and Lck. These derivatives had an inhibitory activity between 48% and 60%. A similar level of inhibitory activity against Lck kinase was observed with derivatives IS18 and IS19. In addition, IS9 (Ar = 4-CH_3_S-C_6_H_4_) exhibited good inhibitory potential against Lyn (57%) and Src (54%) kinases, whereas both proteins were resistant to the IS16 derivative. We found observed that Src kinase was also susceptible to several other derivatives, such as IS6, IS11, IS14, IS15 and IS19. The inhibitory effect detected ranged from 53% to 40%. The most resistant kinase to all compounds tested was BRK. Only in the case of IS1 was a 51% inhibition of BRK activity observed. Our analysis of the results revealed also that IS12 and IS13 derivatives had no impact on the activity of the tested non-receptor kinases. Some derivatives, such as IS4, IS5, and IS17, showed a weak inhibitory activity on most of the kinases, possibly because of their poor solubility, which significantly affects the behaviour of the derivatives in the cellular environment. It is worth mentioning that IS4 (Ar = 2-Br-C_6_H_4_) displayed moderate activity only against BTK kinase.

### Combination therapy

**Figure 3.**
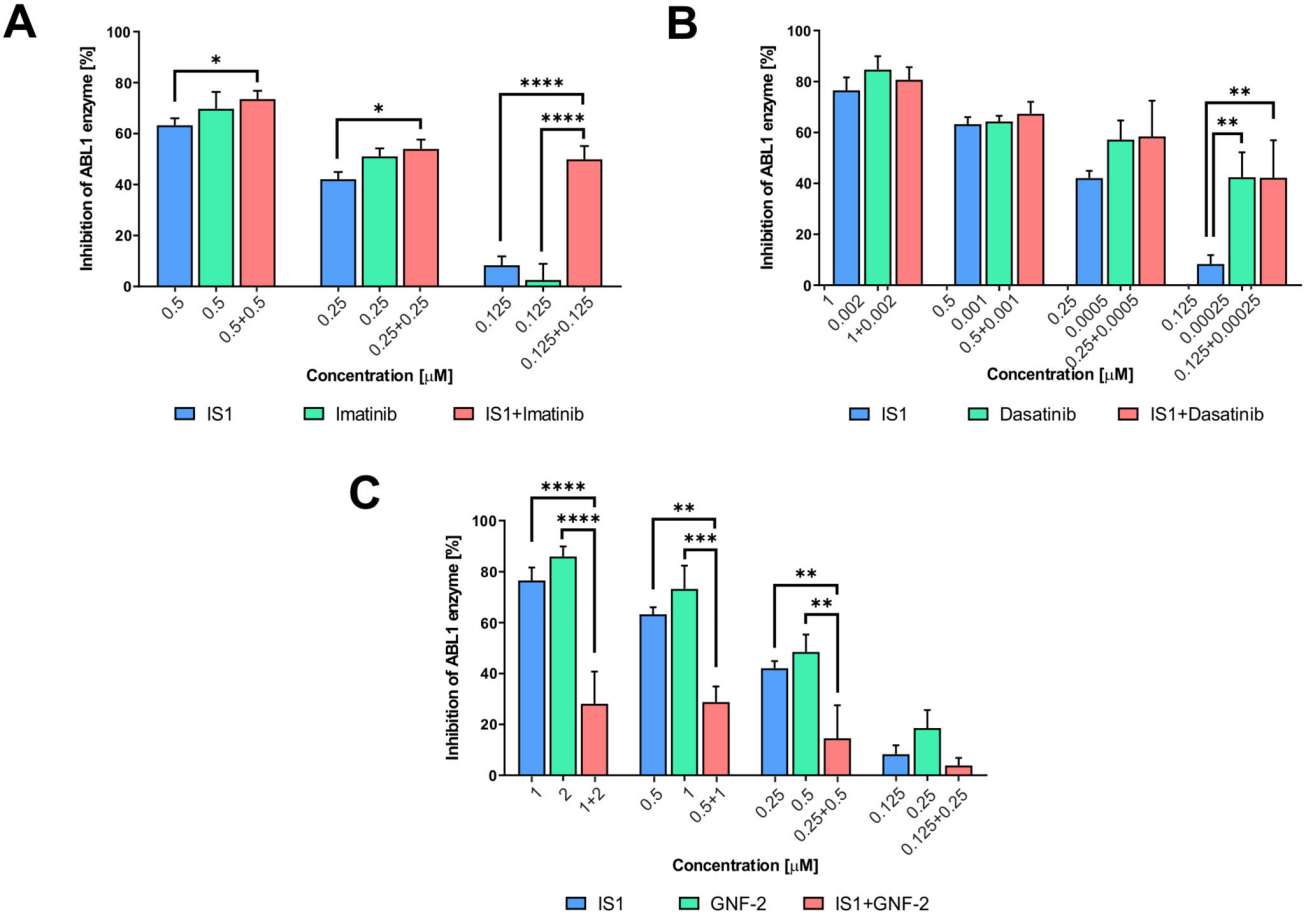
Inhibition of the ABL enzyme after treatment with IS1 styrylquinazoline alone and incombination with imatinib (A), dasatinib (B) and GNF-2 (C). The data were analysed using a two-way ANOVA with Tukey’s post-hoc test: *p < 0.05, **p < 0.01, ***p < 0.001, ****p < 0.0001.

To preliminarily evaluate the interaction of IS1 with ABL kinase, we conducted combination studies with imatinib, dasatinib and GNF-2. We selected the aforementioned reference inhibitors based on their different modes of interaction with the kinases. Namely, imatinib binds preferentially to the DFG-out conformational states of ABL kinase, dasatinib binds to DFG-in and GNF-2 is an allosteric inhibitor that interacts with the myristoyl pocket. Thus, the results of this experiment may allow identification of the site where the IS1 inhibitor binds to ABL kinase. Our analysis revealed a synergistic effect of IS1 in combination with imatinib, with the greatest inhibitory effect observed at the lowest concentration of inhibitors used in combination (Figure 3). The opposite effect was observed when IS1 was combined with dasatinib or GNF-2. We also observed that IS1 and GNF-2 significantly attenuated inhibition when used concomitantly. In light of these data, IS1 appears to have affinity for the orthosteric site of the ABL kinase, but the orientation within the cavity is different than that of imatinib.

These results can be explained on the basis of the conformational changes of the protein in its free and bound forms. ABL kinase initially populates two distinct inactive states that are quite different from each other, whereas the DFG motif is in the “out” conformation in both the i1 and i2 states. Protein kinases tend to adopt a number of different conformational states that have a discrete binding and catalytic activity^30^. In the case of the ABL kinases family, structural data and biochemical studies revealed several autoinhibitory mechanisms that rely on different conformations. In solution, a complex equilibrium occurs, and the existence of a particular form is partitioned in time. In non-transformed cells lacking BCR-ABL1 expression, the kinase is likely inhibited. However, it exhibits a catalytic activity that is enhanced by posttranslational modifications. Phosphorylation unlock the SH2 myristate, which is part of the intrinsic autoinhibitory mechanism. ABL1 kinase is also strongly negatively regulated by its SH3 domain. Overall, autoinhibition promotes the DFG-out alignment of the activation loop. It is worth noting that an inactive DFG-out state prevents phosphorylation of Y412, ATP and substrate binding. Therefore, special care should be taken when interpreting the *in vitro* experimental results, which could be influenced by the reagents and the conditions used.

Herein, we used a kinase assay that contained fully expressed kinases with their regulatory domains, which are responsible for autoinhibition. The proteins are present in their unphosphorylated form and the procedure required supplementation with ATP. Importantly, autophosphorylation of ABL is dependent on low concentration of the protein. Thus, our experimental conditions reflect the cellular process to be simulated. The luminescence signal detected after one hour of incubation with a peptide substrate was a measure of inhibition. Under these conditions, the question of whether the inhibitors tested bind to the ortho or allosteric site of the protein could be answered. Imatinib exhibited a sequential binding mechanism. First, it bound to the inactive DFG-out conformation (i2 state), and then structural changes occurred that decreased overall affinity due to energy expenditure^31^. DFG-out is the least populated state of ABL, regardless of the presence of its SH2 and SH3 regulatory domains. For the full kinase (ABL^FK^), 40% of the population was in an assembled state, in which the regulatory domains were docked to the back of the kinase domain, and 60% of the population was in a disassembled state. The regulatory domains did not stabilise the inactive DFG-flip state. An allosteric inhibitor such as GNF-2 or GNF-5 increased the population of DFG-out up to 95%. This provided an unambiguous interpretation of why the combination of IS1 and GNF-2 led to in an antagonistic outcome (Figure 3(C)), while also clarifying that IS1 is an orthosteric inhibitor. An allosteric inhibitor stabilised the i2 conformational state, increasing the DFG-out population, that was inaccessible to the orthosteric inhibitor (IS1). In this conformation, phenylalanine F382 occupied the ATP binding pocket. Conversely, imatinib caused a shift of the phenylalanine towards the c-lobe, making space for its pyrimidine ring. Therefore, a combination of these drugs showed strong synergy. It was also striking that the effect was considerably smaller at higher concentrations, and that the partial activity of each drug alone was reversed at low concentrations. For example, styrylquinazoline was substantially more active than imatinib at 125 nM (Figure 3(A)). One possible explanation is that imatinib initially saturates the population of i2 that forms the ABL-imatinib complex^32^. Because of equilibrium, saturation occurred to some extent. A concentration of 0.125 nM was too low to saturate most of the i2 population. In this case, it could be hypothesised that IS1 saturates the i1 DFG-flip conformation and thus cooperate with imatinib and that this effect should be stronger at lower concentrations. To test this hypothesis, further molecular docking experiments were performed to more precisely determine the binding of the tested compounds to the ABL kinase.

### Molecular docking

The *in silico* binding mode of IS1 and IS8 with ABL was studied in different conformations of the kinase. Three crystal structures were selected in RCSB PDB, each representing different arrangement of A-loop and Asp-Phe-Gly orientation. The 2HYY-imatinib complex represents the conformational state i2 DFG-out. The complex ABL-imatinib shows the most unfavourable state of the wild type kinase, especially in its apo form. The 4WA9-axitinib complex represents the i1 DFG-flip state and the 2GQG complex with dasatinib represents the active form of the ABL kinase in the DFG-in state. The structures were selected because FDA-approved compounds are commercially available standards supported by strong experimental data. Interestingly, the structure of axitinib contains a styryl and a thioaryl moiety attached to the heterocyclic ring, and thus shows possible similarity to the IS series. However, the Tanimoto index which is a useful measure of chemical similarity, indicates that IS1, for example shows little similarity to axitinib (T_i_ = 0.130) and even less similar to imatinib and dasatinib (T_i_ = 0.048 and 0.067, respectively). The structures are considered similar when the Tanimoto index is > 0.85. The relative docking scores are shown in Table 2. When comparing the docking scores for the same conformation of the kinase between a PDB ligand and IS1 or IS8, the smallest difference (3.889) in docking score was observed for IS1 in the i1 state (DFG-flip). IS8 had a better score than IS1 for the DFG-in ABL. This may indicate that IS1 is a better inhibitor in a kinase assay, as mentioned above. Interestingly, IS8 appears to penetrate deeper into the binding site than IS1 due to its proximity to the C-helix, and to the fact that the hydroxyl group of IS8 forms hydrogen bonds with the NH group of Y253.

**Table 2.** Docking results of the compounds tested with ABL kinase in different conformational states.

Compound	Docking score (kcal/mol)		
	2HYY (DFG-out, i2 state)	4WA9 (DFG-flip, i1 state)	2GQG (DFG-in, ground state)
IS1	−6.158	−8.630	−4.440
IS8	−6.899	−7.204	−7.858
Imatinib	−15.537	–	–
Axitinib	–	−12.529	–
Dasatinib	–	–	−13.725

**Figure 4.**
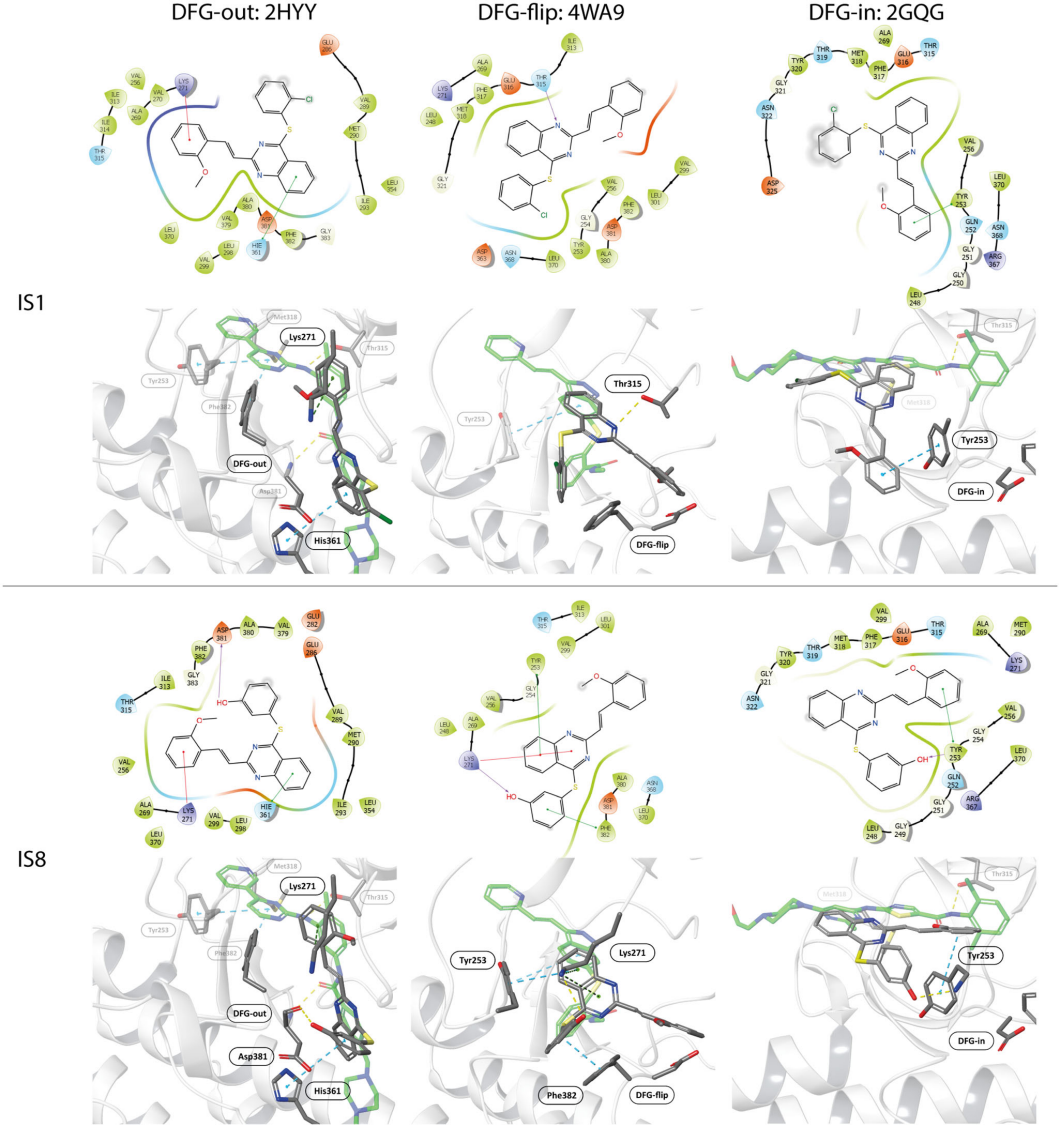
Docking results of the IS1 and I8 derivatives with ABL DFG-out (PDB: 2HYY), ABL DFG-flip (PDB: 4WA9) and ABL DFG-in (PDB: 2GQG). The first lines represent the 2D diagrams of the interactions, whereas the second lines represent the 3D models with the imatinib (DFG-out), axitinib (DFG-flip) or dasatinib (DFG-in) overlay (indicated as a green structure). The 2D and 3D models were generated using Schrodinger Maestro 12 software.

Molecular docking revealed the sensitivity of the tested compounds to substituent exchange with respect to their spatial orientation. Derivatives IS1 and IS8 bind in the selectivity pocket similarly to imatinib and nilotinib (interaction with H361), although the docking score was lower than in the case of imatinib. It is worth noting that the docking experiments support antagonism of IS1 against a GNF-2. The interaction with H361 is characteristic of type II kinase inhibitors, as it is in close contact with both the ATP cofactor and the adjacent selectivity pocket. All kinases tested were able to adopt an active conformation, and only ABL was able to adopt DFG-out. A change from chlorine to hydroxyl had a strong effect on the docking of a ligand to the DFG-in fully active kinase. The docking score was also significantly better for IS8, because the IS1 was much more exposed to the solvent unlike the hydroxyl derivative, which was buried in the nucleotide cleft. The orientation of particular fragments was different. We found that Y253 interacted with the styryl moiety of IS8 (hydrophobic) *via* a face-to-edge and formed hydrogen bonds with the hydroxyl derived from the thioaryl substituent. The styryl moiety of IS1 interacted *via* a face-to-edge hydrophobic interaction with Y253 (Table 3 and Figure 4).

**Table 3.** Interactions between the compounds tested and ABL kinase in the DFG-out, -flip or -in state. YES indicates a distance of less than 3 Å.

	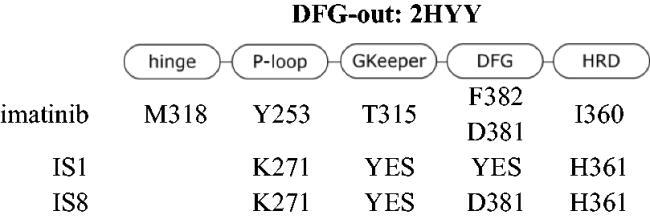				
	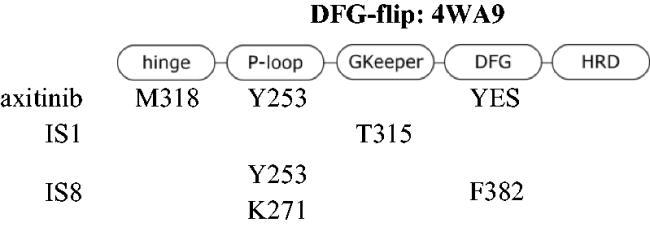				
	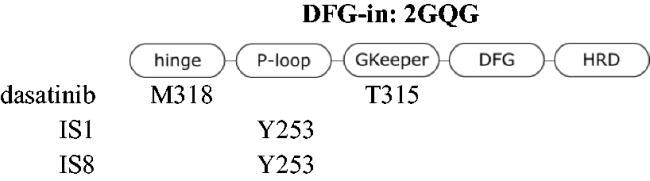				

Molecular docking to DFG-flip kinase showed that both styrylquinazoline inhibitors deeply penetrated into the kinase domain. The respective poses and interactions are shown in Figure 4. Stronger interactions were found for IS8 where Y253 interacted with the quinazoline ring (face-to-edge), K271 with quinazoline (pi-cation) and with the hydroxyl group of the thioaryl moiety, which was also in face-to-face contact with F382 of DFG motif. IS1 exhibited a similar orientation although a different conformation of the thioaryl fragments was observed (180 degree flip). This moiety was able to interact with Y253 and F382 (face-to-edge), whereas quinazoline N-1 interacted with the gatekeeper T315. These observations help explain what was discussed above. If the ligands target the same binding pocket, synergistic interactions should be to some extent limited. Another effect can be observed when one or more drug molecules stabilise a specific conformation^33^. The stronger inhibitory effect of IS1 compared with imatinib in the kinase assay could be explained by the fact that imatinib stabilises the DFG-out conformation, but the protein is trapped in the active DFG-in conformation after phosphorylation of ABL. Imatinib forces an ABL-like DFG-out conformation and is therefore an ABL selective ATP-competitive inhibitor. As a result, imatinib uses energy for a conformational change. Finally, its IC_50_ is relatively higher than that of dasatinib. In contrast, the allosteric inhibitor GNF-2 interact with the myristate site and stabilises the DFG-out-like motif. Nuclear magnetic resonance experiments on p38 kinase showed that the DFG-in and DFG-out conformations are in a dynamic equilibrium over relatively short time scales (milliseconds)^34^. Similarly, Src and ABL can switch between two inactive conformations: an ABL-like, DFG-out inactive conformation and a Src-like, DFG-in inactive conformation (corresponding to the i1 state - DFG-flip of ABL). On the time scale of the experiment, different forms of the kinase populated in the solution and the signal detected after a one-hour run was the overall result of inhibition. A combination of IS1 and GNF-2 (an allosteric inhibitor), which stabilised the kinase in the DFG-out conformation and thus suppressed conformational exchange, resulted in an antagonistic outcome. Combination of IS1 with dasatinib had virtually no effect on ABL inhibition. Therefore, IS1 can be assumed to stabilise ABL in an i1 - inactive state, whereas IS8 does not.

### Antiproliferative activity of styrylquinazoline derivatives

The antiproliferative properties of all synthesised compounds were tested on a panel of seven different human cancer cell types, including chronic myeloid leukaemia cells (K562) with p53 and ABL protein mutations and solid tumours, such as pancreatic ductal adenocarcinoma (PANC-1), wild type colon carcinoma (HCT 116 p53^+/+^) and a mutant with a deletion of the *TP53* gene (HCT 116 p53^-/-^), lung cancer (A549), breast cancer (MCF-7) and glioblastoma (U-251). Glioblastoma is the most aggressive cancer and has a poor prognosis and short survival after application of available treatments^35^. In contrast, the other cell lines studied represent common cancers found in the human population. Nevertheless, these cell lines are characterised by a different landscape of kinase expression, as reported in^36^. In chronic leukaemia cells, an aberration of the Philadelphia chromosome is common in which a fragment of chromosome 9 is displaced to chromosome 22, leading to the formation of the oncogenic protein BCR-ABL. The altered structure of the protein leads to abnormal activity that promotes excessive growth and differentiation of cells even in the absence of growth factors, as well as inhibition of apoptosis and formation of metastases^37^. Moreover, the status of the *TP53* gene in leukaemia is not insignificant. Mutations of the *TP53* gene occur in more than 50% of all cancers, leading to increased metastasis and an overall poor prognosis^38^^,^^39^. The p53 protein encoded by this gene plays a pivotal role in controlling cell division or cell fate when damage is detected. On the other hand, the presence of a non-functional protein caused by missense or null mutations of the *TP53* gene in leukaemia cells has been associated with CML progression or resistance to the kinase-dependent inhibitors used^40^. Several reports have indicated that the p53 pathway can be controlled by kinases, such as c-ABL, Src or Aurora^41^^,^^42^. Therefore, interaction between the signalling network of the BCR-ABL pathway and p53 often results in an impairment of p53 function and survival^43^^,^^44^. For example, altered down-regulation of p53 by c-ABL has been reported in colon cell lines with a wild-type or null p53 status^45^. It is noteworthy that several reports have described the involvement of Lyn, BTK or Src in resistance to drug therapy by preventing the induction of apoptosis^46^^,^^47^. Selectivity studies were performed on normal human fibroblast – NHDF cell line. The results are shown in Table 4.

**Table 4.** Antiproliferative activity of the studied derivatives against a panel of cancer cells and normal fibroblast (NHDF) cell line.

Compound	Antiproliferative activity IC_50_ [µM]							
	K562	PANC-1	HCT 116 p53^+/+^	HCT 116 p53^−/−^	MCF-7	A549	U-251	NHDF
Series A								
IS1	6.434 ± 1.197	>25	>25	>25	18.52 ± 3.87	>25	>25	>25
IS2	24.76 ± 2.45	25.13 ± 1.44	23.79 ± 5.32	NT	19.69 ± 1.13	NT	>25	NT
IS3	>25	NT	>25	>25	>25	NT	NT	NT
IS4	>15	>25	>10	>10	NT	>15	>15	>25
IS5	>25	>25	>25	NT	>25	NT	>25	NT
IS6	>25	>25	>25	NT	>25	NT	>25	NT
IS7	>10	>15	>15	>15	NT	>15	>15	>20
IS8	1.088 ± 0.299	17.85 ± 1.32	>25	>25	8.034 ± 0.958	>25	25.03 ± 2.53	>25
IS9	>25	>25	>20	>20	NT	>15	>10	>25
IS10	2.042 ± 0.454	>25	10.74 ± 0.33	9.427 ± 1.485	16.26 ± 3.22	>25	>25	>25
IS11	>2.5	>10	>15	>15	NT	>5	>5	21.70 ± 1.53
Series B								
IS12	>25	NT	>25	>25	>25	>25	>25	NT
IS13	>25	NT	>25	>25	>25	>25	>25	NT
IS14	21.63 ± 2.10	>25	>25	NT	>25	NT	>25	NT
IS15	21.25 ± 2.19	>25	>25	>25	>25	>25	>25	>25
Series C								
IS16	7.835 ± 0.753	>2.5	>15	>10	>25	13.44 ± 0.60	20.91 ± 1.63	14.57 ± 1.27
IS17	>10	>15	>15	>15	NT	>15	20.15 ± 0.90	21.30 ± 0.80
Series D								
IS18	>25	12.02 ± 1.20	11.05 ± 1.374	NT	10.62 ± 1.375	NT	10.30 ± 0.818	NT
IS19	>25	16.86 ± 1.12	13.21 ± 3.87	NT	14.06 ± 0.68	NT	7.843 ± 3.024	NT
References								
CP-31398	3.087 ± 0.36	>25	18.63 ± 0.92	26.28 ± 1.41	26.96 ± 2.10	>25	18.77 ± 1.65	12.26 ± 0.54
imatinib	0.133 ± 0.03	>25	44.55 ± 2.41	51.21 ± 4.09	>25	>25	>25	>25
GNF-2	0.208 ± 0.045	>25	>25	>25	>25	>25	>25	>25

NT: not tested.

In general, leukaemia cells were most vulnerable to the compounds tested. The highest activity of our novel compounds was observed for the IS8 derivative (Ar = 2-Cl-C_6_H_4_ attached to the S^4^-quinazoline core). The calculated IC_50_ value was 1.088 µM. Another derivative (Ar = 1,3-benzodioxol-5-yl) characterised by a high sub-micromolar activity level was IS10 (IC_50_ = 2.042 µM). Notably, both derivatives were inactive against normal cells (IC_50_ >25). The selectivity index for IS8 and IS10 was higher than 22.98 and 12.24, respectively (Table S1 in Supporting Information). In contrast, attachment of a bromine atom to the 1,3-benzodioxol-5-yl ring (IS11) at position 6 decreased the solubility of the compound, rendering it inactive against the cancer cell lines examined. Moreover, IS11 had a low cytotoxic effect on the fibroblast cells (IC_50_ = 21.7 µM). IS15 and IS17 with the 6-Br-1,3-benzodioxol-5-yl group had a similar cytotoxicity profile against the cell lines tested. Interestingly, the IS1 derivative (Ar = 2-Cl-C_6_H_4_) attached to the S^4^-quinazoline ring, which showed the highest potential to inhibit ABL kinase, had good, although not the best, anticancer activity against K562 cells. The calculated IC_50_ was 6.434 µM. IS1 showed a weak activity against the MCF-7 cells, for which the IC_50_ value was 18.52 µM. Its analogue, IS16 bearing the same (Ar = 2-Cl-C_6_H_4_) S^4^-aryl substituent, although attached to a 7-chloroquinazoline ring, exhibited a similar level of activity on leukaemia cells (IC_50_ = 7.835 µM). Moreover, it had only moderate activity against A549 lung cancer cells (IC_50_ was 13.44 µM). In fact, the A549 cell line and the HCT 116 p53^-/-^ cell lines were the most resistant to the tested compounds, including imatinib and GNF-2. In the case of the HCT 116 mutant cells, only the IS10 derivative showed good activity, with an IC_50_ of 9.427 µM. In comparison, CP-31398, a reactivator of the p53 protein, showed little antiproliferative effect (IC_50_ = 26.28 µM). In wild-type HCT 116 cells, three derivatives, IS10, IS18 and IS19, had moderate antiproliferative activity. The calculated IC_50_ values were above 10 µM (10.74 µM for IS10, 11.05 µM for IS18 and 13.21 µM for IS19). The IS18 derivative, which has the same S^4^-substituent (Ar = 2-Cl-C_6_H_4_) as IS1, but different fragment of the styryl moiety (4-MeO-C_6_H_4_ instead of 2-MeO-C_6_H_4_ as found in the majority of the IS series), had similar activity against breast cancer (IC_50_ = 10.62 µM), pancreatic cancer (IC_50_ = 12.02 µM) and glioblastoma cells (IC_50_ = 10.30 µM). However, in the U-251 cell line, IS19 with the 1,3-benzodioxol-4-yl group attached to the styryl moiety had better activity (IC_50_ = 7.843 µM). Moreover, the lowest IC_50_ value (8.034 µM) for MCF-7 cells was found for the IS8 derivative. Similar to leukaemia cells, the IS10 derivative had a two-fold less active level (16.26 µM) against breast cancer cells than IS8. It is worth noting that the reference ABL inhibitors, such as imatinib and GNF-2, were inactive against all solid tumours tested. Due to their known single-target nature, these derivatives exhibited high activity levels only on K562 cells, characterised by elevated ABL gene and protein expression levels^36^^,^^48^. On the other hand, the single-target nature of the potential inhibitors might allow the development of resistance mechanisms in cells. In the case of imatinib, these typically include a *BCR-ABL* gene amplification, gene mutation, incomplete inhibition, altered binding affinity and a P-glycoprotein overexpression^49^. In addition to BCR-ABL, the JAK/STAT, Raf/MEK/ERK and PI3K/Akt signalling pathways may also play important roles in leukemogenesis and cell cycle regulation^50^. Therefore, compounds targeting multiple molecular targets and signalling pathways may be more valuable due to their multifaceted mechanism of action and their ability to overcome resistance.

### Styrylquinazoline derivatives induced cell cycle arrest

The deeper molecular mechanism of action of the novel styrylquinazolines, concerning effects on cell cycle arrest, apoptosis induction and impact on modulation of the expression of genes and proteins related to the ABL signalling pathway, we investigated on the K562 cell line. This cell line was selected due to the high expression of the BCR-ABL protein, as reported elsewhere^37^^,^^51^. In addition, our previous work has determined *ABL* gene expression in several cell lines examined in this study^36^.

The cellular level of ABL kinase is tightly controlled due to its involvement in cell growth, adhesion and cell cycle regulation^52^. Interestingly, nuclear and cytoplasmic ABL kinase exerts antagonistic effects on cell proliferation and stress response in normal and cancer cells, respectively. In normal cell development, c-ABL kinase binds the p53 protein after DNA damage, thereby enhancing its DNA binding and transcriptional activity^53^. This effect leads to negative stimulation of cell growth and induces cell arrest and apoptosis *via* the p53 pathway in normal cells. Leukaemia cells. on the other hand, express the cytoplasmic mutant protein BCR-ABL, which is constitutively active and can cause a different cell cycle delay outcome, leading to inhibition of caspase activation and protection from apoptosis^51^. Notably, this protein can promote cell cycle entry in CML cells in the absence of growth factors^54^. Moreover, the BCR/ABL cells may be more susceptible to accumulation of DNA damage after genotoxic stress because the repair system is impaired^55^^,^^56^. A similar effect has been reported for styrylquinolines^19^. Moreover, in the absence of ABL or p53, mutant cells lose the G1/S checkpoint and survive despite treatment^53^. ABL also regulates cell growth by affecting actin polymerisation and the dynamics of actin filament rearrangement within the cytoskeleton^57^.

**Figure 5.**
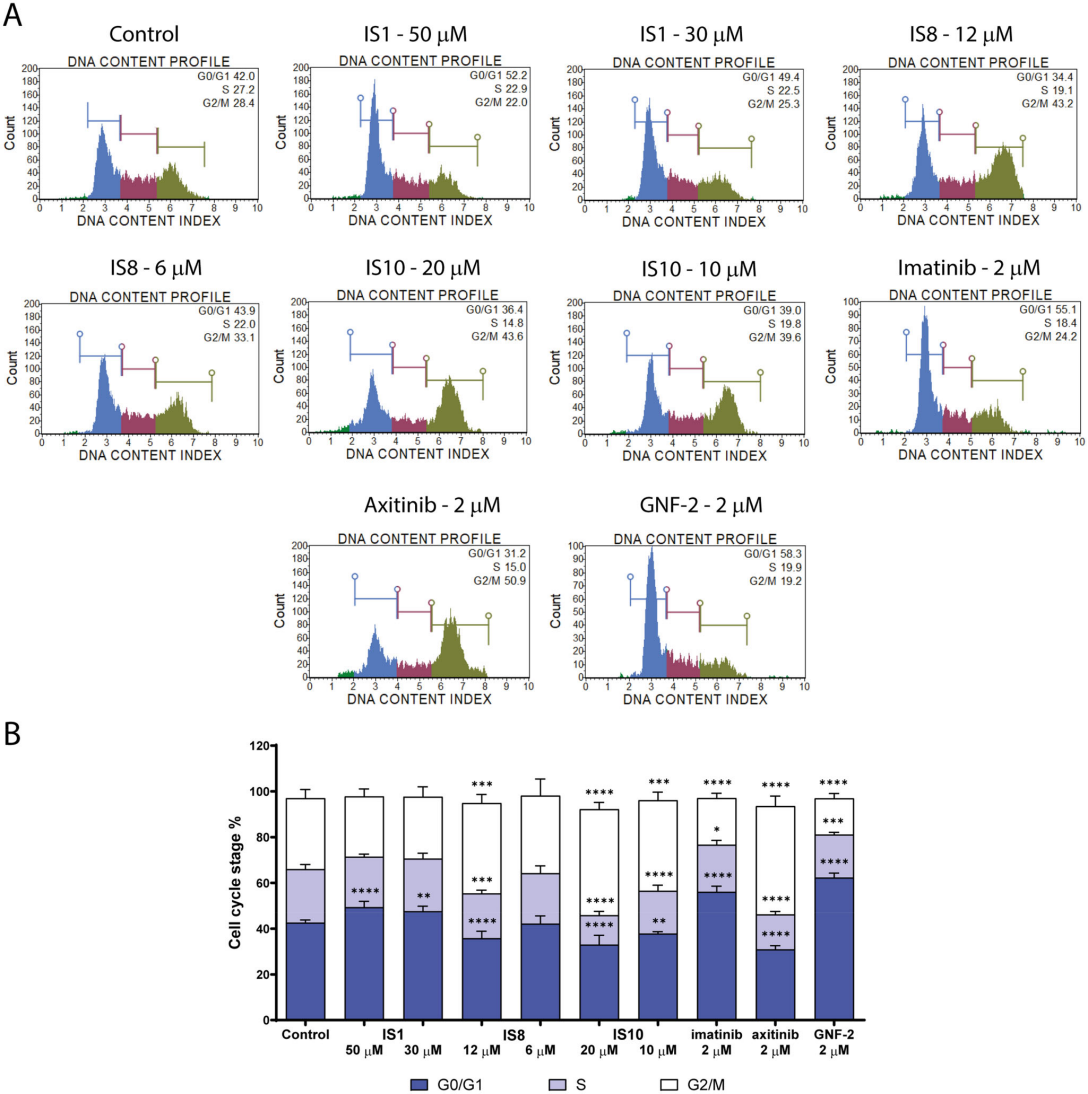
Effect of IS1, IS8, IS10, imatinib, axitinib and GNF-2 on the progression of the cell cycle in K562 cells. The flow cytometry histograms show the distribution of cells in the G0/G1, S and G2/M phases of the cell cycle for one of the experiments (A). The data were analysed using a one-way ANOVA with Dunnett’s post-hoc test: *p < 0.05, **p < 0.01, ***p < 0.001, ****p < 0.0001 compared with the control (B).

The interesting inhibitory potential of ABL kinase and the high antiproliferative activity of several styrylquinazoline derivatives prompted us to further investigate their mechanism of action. As a first step, we analysed the effect of the compounds (IS1, IS8 and IS10) that were most active on cell cycle progression in leukaemia cells. The results of flow cytometry assays are shown in Figure 5. In general, the tested compounds exhibited different patterns of cell cycle arrest. In the case of the IS1 derivative, there was a significant increase in the population of cells in the G0/G1 phase from 42.56% (untreated cells) to 50.85% with a concomitant decrease in DNA content in S and G2/M phases. Similar results were observed for the reference compounds imatinib and GNF-2, which caused inhibition of G0/G1 phase of the leukaemia cell cycle. These results for the reference inhibitors are consistent with literature data^58^.

In contrast, the other two styrylquinazolines studied caused cell cycle inhibition in G2/M phase. Namely, IS8 at a concentration of 12 µM caused a statistically significant increase in the fraction of cells in G2/M phase from 28.68% (control cells) to 39.38%. In turn, the 1,3-benzodioxole derivative (IS10) caused a significant increase (46.35%) of cells in G2/M. As expected, the cell population in G0/G1 and S phases decreased significantly to 33.00% (from 42.56% in the untreated cells) and 12.85% (from 23.66% in the control), respectively. For axitinib, there was a similarly significant increase in the fraction of cells in the G2/M phase to 47.28%.

### Styrylquinazoline derivatives induce apoptosis

**Figure 6.**
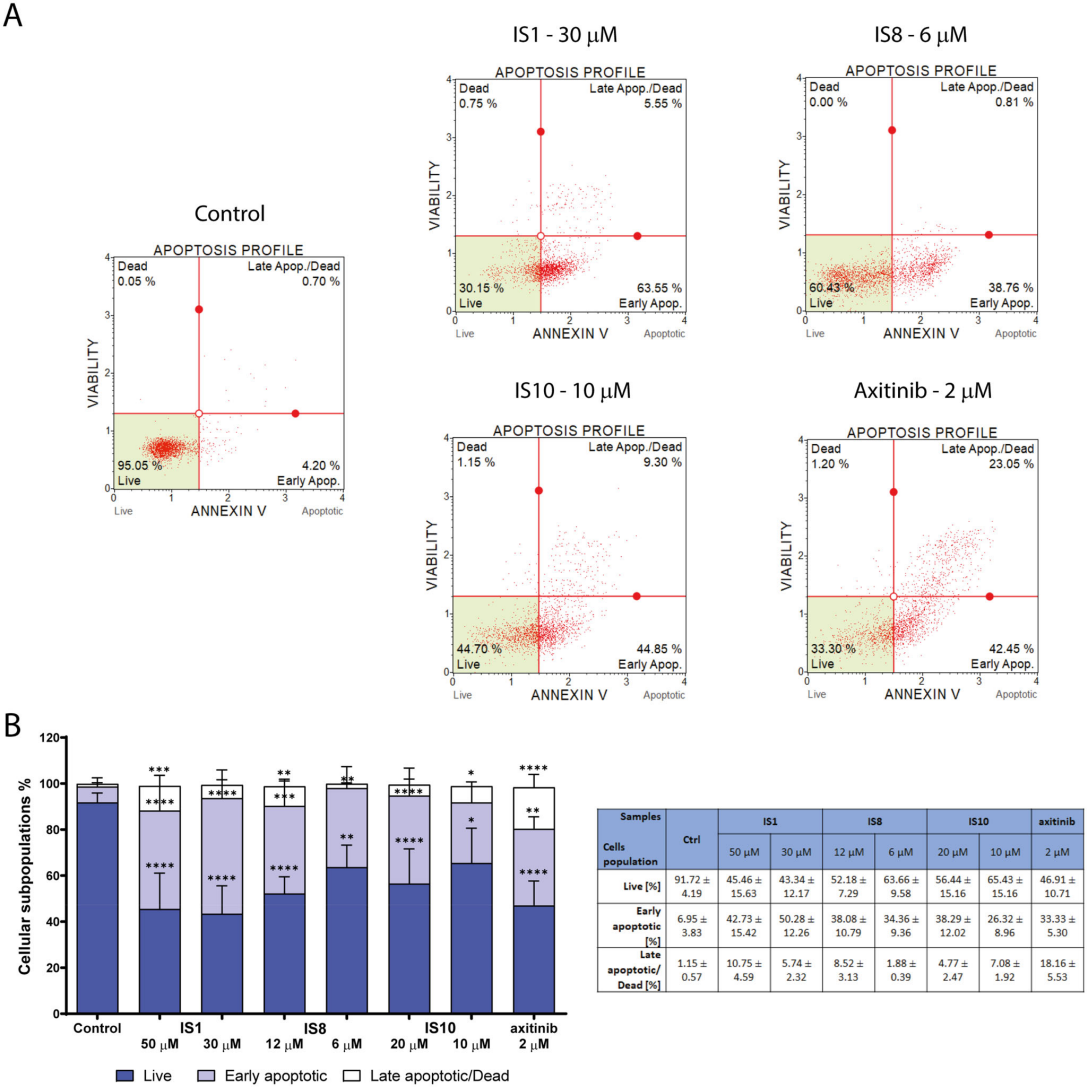
Evaluation of apoptosis induction in K562 cells after a 30-h treatment with IS1, IS8, IS10 and axitinib. The flow cytometry histograms indicate the percentage of early and late apoptosis for one of the experiments (A). The data were analysed using a one-way ANOVA with Dunnett’s post-hoc test: *p < 0.05, **p < 0.01, ***p < 0.001, ****p < 0.0001 compared to the control. The table includes the mean ± SD percentage of the live, early and late apoptotic cells from all of the conducted experiments (B).

As mentioned above, regulation of ABL kinase may be crucial for apoptosis induction and the efficacy of treatment. Therefore, the next step was to investigate apoptosis induction in K562 cells after 30 h of treatment with the tested styrylquinazolines. For this purpose, annexin V-FITC and 7-ADD staining were performed, and the fluorescence emitted from damaged cells was quantified using a flow cytometer. Annexin V is a well-known apoptosis marker. When cells are damaged, the phosphatidylserine that is located inside the cell membrane changes its position and arranges itself on the cell surface. This behaviour is an early feature of the initiation of apoptosis in cells. Phosphatidylserine that is located on the cell surface becomes accessible to annexin V-FITC, which has a high affinity for it. The results of these experiments are shown in Figure 6. In general, the percentage of apoptotic cells increased in a concentration-dependent manner after administration of the tested compounds. Moreover, in all cases, the highest percentage of cells was in the early phase of apoptosis. The highest increased population of apoptotic cells was in cells after exposure to the IS1 derivative at both concentrations. There was a significant increase in the percentage of apoptotic cells (early and late) from 8.1% (in control cells) to 53–56%. For the IS8 derivative, there was an increase in the total number of apoptotic cells ranging from 46.6% to 36.24%. The third derivative studied, IS10, caused a slightly smaller apoptotic effect (increase in the population of apoptotic cells to 33.4–43.06%). Similar to IS1, the reference inhibitor axitinib also elicited an apoptotic effect.

### Effects of styrylquinazoline derivatives on ABL signalling

**Figure 7.**
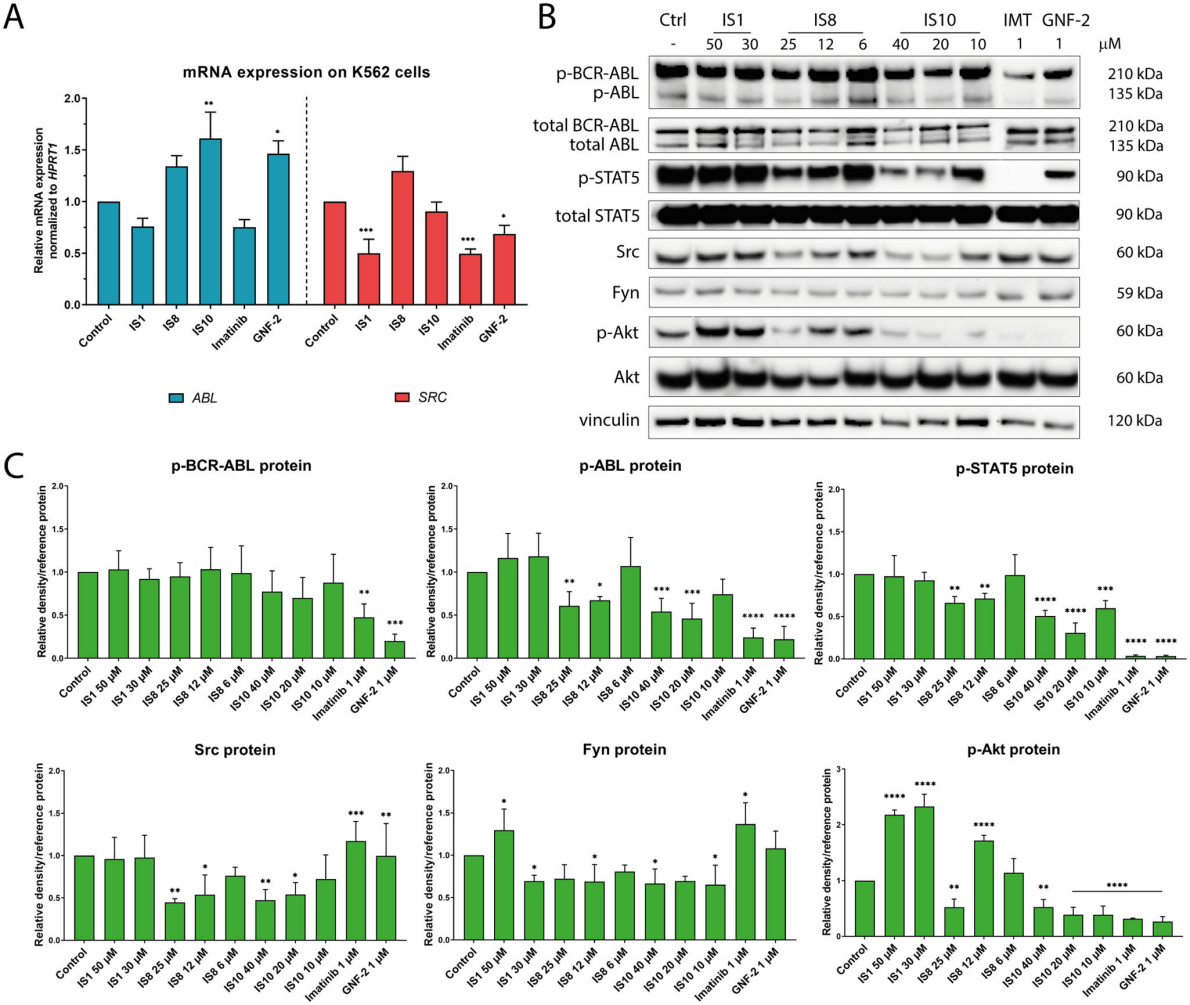
Effect of the derivatives tested for activation of the ABL pathway and its downstream targets (A, B). The densitometric analysis plots show the obtained results normalised to the reference protein (vinculin, GAPDH or β-actin). Results are shown as the mean value ± *SD* from three independent experiments (C). The data were analysed using a one-way ANOVA with Dunnett’s post-hoc test: *p < 0.05, **p < 0.01, ***p < 0.001, ****p < 0.0001 compared to the control.

In the next step, the inhibitory potential of the tested compounds at the gene and protein levels was verified in K562 cells. For this purpose, we examined changes in the expression of the ABL signalling pathway and ABL downstream targets after exposure to IS1, IS8 and IS10. As shown in Figure 7(A), IS1 and imatinib caused a slight non-significant decrease in the expression of the *ABL* gene, while IS10 and GNF-2 significantly enhanced *ABL* transcription. The enhanced transcription of the *ABL* gene might be related to the low protein level that was reached after exposure to the tested drugs. Thus, the cellular machinery could induce increased protein synthesis to counteract the effects of inhibition. In addition, a significant decrease in *SRC* mRNA levels was observed after exposure to IS1, imatinib, and GNF-2. The second gene studied, *SRC*, is involved in signal transduction from growth factors and receptors. Similar to ABL, the Src protein has oncogenic activity and is overexpressed in cancer cells. It is also important to emphasise that both kinases form a specific direct signalling network, as ABL kinase is an important substrate required for Src activation^59^.

Changes in the expression of the BCR-ABL and ABL proteins and their downstream targets, such as STAT5, Src, Akt and Fyn after treatment with the tested compounds were also examined. As shown in Figure 7(B), no significant changes in the phosphorylation of BCR-ABL were observed after treatment with the tested styrylquinazolines. Interestingly, a different situation was observed in the case of the ABL protein. Namely, IS8 and IS10 caused a significant decrease (about two-fold) in ABL phosphorylation. For reference inhibitors, there were greater changes in the expression of this protein. There were also interesting changes in the downstream targets of ABL, STAT5 and Src. IS8 (at 12 and 6 µM) caused a significant 1.5-fold inhibition of STAT5 phosphorylation at Tyr694. In addition, IS10 at 20 µM caused a more than three-fold decrease in p-STAT5 expression. A stronger effect was observed for imatinib and GNF-2. The reverse situation was observed for the Src protein. Both styrylquinazolines, IS8 and IS10, caused an almost two-fold decrease in Src expression, whereas imatinib and GNF-2 caused a significant accumulation of this protein. Unfortunately, contrary to our assumptions and earlier results, IS1 was ineffective in inhibiting ABL, STAT5, and Src phosphorylation at the concentrations used. However, this derivative caused downregulation of Fyn expression, which could confirm its high inhibitory potential from *in vitro* kinase assays. Similar changes were observed after exposure to IS8 and IS10. Our analyses showed that IS8 (only at 25 µM), IS10, and the references inhibited the expression of Ser473 phosphorylated Akt, another protein involved in ABL signalling, by more than two-fold. Thus, these compounds were capable of blocking the pro-survival pathway facilitated by Akt kinase.

### Effect of the styrylquinazoline derivatives on cell cycle inhibition and apoptosis induction routes

**Figure 8.**
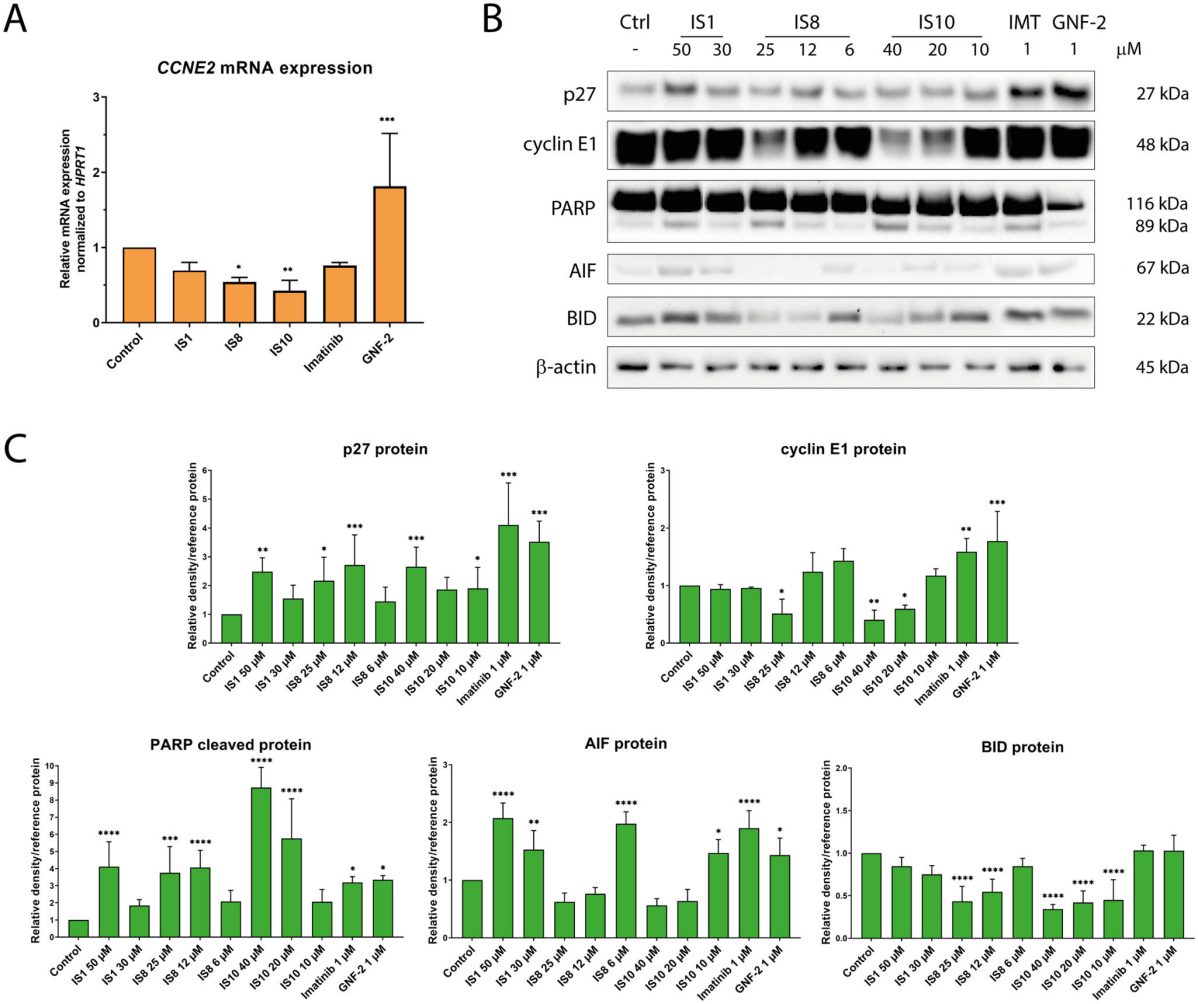
The impact of the tested styrylquinazoline on the expression of the genes (A) and proteins (B) involved in cell cycle progression and apoptosis induction. Densitometric analysis results have been normalised to reference proteins (vinculin, GAPDH or β-actin). Results are shown as mean ± *SD* from three independent experiments (C). Data were analysed using a one-way ANOVA with Dunnett’s post-hoc test: *p < 0.05, **p < 0.01, ***p < 0.001, ****p < 0.0001 compared to the control.

Finally, we investigated the effects of the tested derivatives on *CCNE2*, cyclin E1 and p27, involved in cell cycle progression, as well as PARP, AIF and BID related to apoptosis induction pathway. *CCNE2* encodes a cyclin E2 protein involved in the G1/S phase transition. Together with cyclin E1, cyclin E2 forms a complex with Cdk2, which phosphorylate various targets, leading to cell cycle progression^60^. It should be noted that several reports have shown the role of cyclin E in triggering cell death^61^^,^^62^. Another protein, p27, is a cyclin-dependent kinase inhibitor and a negative regulator of cell cycle progression. p27 can bind to the cyclin E2-Cdk2 complex, inhibiting its activity and preventing the transition from G1 phase to DNA synthesis. In addition, the aforementioned complex leads to phosphorylation of p27, which induces ubiquitin binding and its degradation in the proteasome^63^. It is also worth noting that several reports have shown that inhibition of the BCR-ABL protein can affect p27 protein recovery, and thus cell cycle arrest^64^. qRT-PCR analysis revealed that IS8 and IS10 derivatives caused a significant decrease in *CCNE2* expression in K562 cells (Figure 8(A)). Conversely, imatinib caused an increase in *CCNE2* transcript. At the protein level, cyclin E1 was significantly downregulated by IS8 (only at 25 µM) and IS10 (40 and 20 µM). As expected, these derivatives caused an more than two-fold increase in p27 expression. The IS1 compound had a similar effect on the p27 protein. Interestingly, both references resulted in upregulation of cyclin E1 and p27.

All tested compounds caused cleavage of the PARP protein, a hallmark of apoptosis, in K562 cells. This protein is responsible for DNA repair, chromatin remodelling and metabolic regulation. When PARP is cleaved and inactivated by caspases, cells disintegrate and enter the final stage of cell death. The other pro-apoptotic protein examined was BID, which is cleaved by caspases and released from mitochondria into the cytoplasm. As shown in Figure 8(B,C), only IS8 and IS10 were able to decrease the level of BID by approximately 2-fold. The effects on AIF protein changes were also remarkable. The IS1, IS8 derivatives, as well as imatinib caused a significant increase in AIF protein, which is known to be a caspase-independent effector of apoptosis death. These results suggest that the compounds used in this study can induce apoptosis through caspase-dependent and -independent pathways.

## Conclusion

A series of novel styrylquinazolines with a phenylthiol group in the C4 position was designed and synthesised based on our previous results for CP-31398 analogues. Their inhibitory activity against eight non-receptor tyrosine kinases was determined. Several compounds showed inhibitory activity against ABL and Src family kinases. Among them, IS1 (0.5 µM) caused almost 91% inhibition and IS8 almost 55% inhibition of ABL activity. Further docking studies revealed different binding modes for the IS1 and IS8 derivatives, indicating interaction with the DFG-flip and DFG-flip/DFG-in conformational states, respectively. Cellular studies were performed to capture the full landscape of activity and possible therapeutic applications of the novel derivatives. The three derivatives exhibited anticancer activity in the sub-micromolar level against leukaemia cells and desirable selectivity against normal fibroblast cells. Detailed analysis revealed different behaviours of the IS1 and IS8 derivatives in the cellular environment, an observation possibly related to the mode of binding to ABL kinase. IS1 caused cell cycle arrest in G0/G1 phase, while IS8 arrested the cells in G2/M phase. Protein analysis showed that IS8 was able to inhibit phosphorylation of ABL kinase as well as that of its downstream targets such as STAT5 and Src. In contrast, IS1 inhibited Fyn at the cellular level, whereas it was ineffective in inhibiting ABL, STAT5, and Src signalling. Both styrylquinazolines acted on PARP, BID, and AIF to trigger apoptosis *via* caspase-dependent and -independent pathways. In conclusion, these results provide new insights into the behaviour and mechanism of action of styrylquinazoline derivatives at the cellular level. Thus, these data may be useful to develop new multi-target inhibitors characterised by a desirable mode of binding to kinases that makes them effective in anticancer therapy.

## Experimental section

Fourier transform nuclear magnetic resonance spectra of the sample solutions were obtained using a Bruker Ascend 500 for ^1^H (500 MHz), and chemical shifts are reported in *δ* units (parts per million) relative to tetramethylsilane and the peaks of the residual solvent are used as reference. Chemical synthesis was performed with the Microwave Synthesiser Discover 2.0 (CEM Corporation) or using a heating plate with magnetic stirring. High-resolution mass spectra were measured using a DionexUltiMate^®^ 3000 high-performance liquid chromatograph (Thermo Fisher Scientific, West Palm Beach, FL, USA) coupled to an LTQ Orbitrap XL™ Hybrid Ion Trap-Orbitrap Fourier Transform Mass Spectrometer (Thermo Fisher Scientific) with injection into HESI II in positive or negative modes. Chromatographic separations were performed using a Teledyne ISCO Combiflash Rf150+. NMR samples were prepared at concentrations in the range of 5–10 mM. The purity of the styrylquinazolines was confirmed by HPLC and was at least 95%. The arylthiols, except those described in Scheme 1(B) with a methylenedioxy group, were purchased from Merck. The reference inhibitors: imatinib, axitinib, dasatinib and GNF-2 were purchased from Merck. The CP-31398 dihydrochloride (*N*'-[2-[2–(4-methoxyphenyl)ethenyl]-4-quinazolinyl]-*N*,*N*-dimethyl-1,3-propanediamine dihydrochloride) was purchased from Cayman Chemical Company. Preferred IUPAC names (according to the IUPAC blue book 2013) were generated in Chemdoodle software – version 11.13.0).

### Synthesis

2-Methylquinazolin-4(3*H*)-ones, further 2-[(*E*)-2-phenylethenyl]quinazolin-4(3*H*)-ones and their benzenesulfonate derivatives were synthesised according to previously reported protocols (see Scheme 1)^22^, and several others methods for synthesis of the precursors used can be found. We used 2-aminobenzoic acid (or its analogue) as the starting material and heated it in the presence of 20-fold excess acetic anhydride in a microwave reactor (80 W, 80 °C, 40 min). The precipitate formed was filtered and washed with 2-propanol (volume equivalent to one filter cake). This procedure yields pure 2-methyl-*4H*-3,1-benzoxazin-4-ones. After air drying, solids were suspended in an aqueous solution of ammonia (32% w/w) and stired for 7 days. The solid was then filtered through a funnel and the filter cake was washed with water and then with 2-propanol (volume equivalent of a filter cake). The compounds were recrystallized from 99% ethanol to yield pure 2-methylquinazolin-4(*3H*)-one or its analogue. It is important to avoid the presence of the starting material (2-methyl-*4H*-3,1-benzoxazin-4-ones) at this stage because it can react with aldehyde in the next step causing potential purification problems. However, we did not find any impurities when using our method. Styrylquinazolinones (SQ) were obtained by reaction with aryl aldehydes in pure acetic acid (2 M solution of a substrate) as reaction medium. Reactions were carried out in a microwave reactor (80 W, 130 °C, 80 min). After cooling to ambient temperature, the precipitate was filtered through a funnel and washed with 2-propanol (2x volume of filter cake) and air dried. Benzenesulfonates were prepared by adding tosyl chloride (1 eq), DIPEA (1 eq) and DMAP (0.05 eq) to the suspension of 2-styrylquinazolinone (0.2 mmol) in 5 ml CH_2_Cl_2_ at room temperature. A good indication of the progress of the reaction is the disappearance of the suspension. The solution was then separated by flash chromatography. The first fraction, eluted with CH_2_Cl_2_ contained unreacted tosyl chloride. Benzenesulfonate was collected as the second fraction. The solvent was removed *in vacuo* (30 °C). Target compounds were synthesised *via* a general route by reacting a benzenesulfonate (1 eq) with an arylthiol compound (1 eq). Heating the reaction mixture to 80 °C in 2-propanol and addition of equivalent amount of *N*,*N*-diisopropylethylamine (DIPEA) produced the target compounds which crystallised from the reaction medium. Washing the crude compound with 2-propanol generally leads to pure compounds.

#### IS1 2-[(E)-2–(2-methoxyphenyl)ethenyl]-4–(2-chlorophenylthio)quinazoline

^1^H NMR (500 MHz; (CD_3_)_2_SO): *δ* 8.22 (ddd, *J* = 8.2, 6.8, 0.6 Hz, 1H), 8.00 (ddd, *J* = 8.2, 6.9, 1.4 Hz, 1H), 7.94 (ddd, *J* = 8.5, 1.4, 0.7 Hz, 1H), 7.90 (dd, *J* = 7.7, 1.6 Hz, 1H), 7.82 (dd, *J* = 8.0, 1.3 Hz, 1H), 7.75–7.70 (m, 2H), 7.70 (d, *J* = 16.3 Hz, 1H), 7.60 (dd, *J* = 7.9, 1.5 Hz, 1H), 7.58 (apptd, *J* = 7.4, 1.3 Hz, 1H), 7.34 (ddd, *J* = 8.4, 7.2, 1.7 Hz, 1H), 7.13 (d, *J* = 16.0 Hz, 1H), 7.05 (dd, *J* = 8.4, 1.2 Hz, 1H), 6.97 (apptd, *J* = 7.7, 0.8 Hz, 1H), 3.86 (s, 3H); ^13^C NMR (126 MHz, (CD_3_)_2_SO): *δ* 168.53, 159.63, 157.77, 149.34, 139.57, 138.98, 135.22, 133.97, 132.54, 131.20, 130.74, 128.79, 128.62, 128.13, 127.91, 127.58, 126.57, 124.31, 124.16, 121.29, 121.17, 112.11, 56.00; HRMS (ESI) calcd for C_23_H_17_ClN_2_OS ${\left[M+H\right]}^{+}$ 405.0823; found 405.0827.

#### IS2 2-[(E)-2–(2-methoxyphenyl)ethenyl]-4–(3-chlorophenylthio)quinazoline

^1^H NMR (500 MHz, (CD_3_)_2_CO): *δ* 8.21 (ddd, *J* = 8.2, 1.4, 0.7 Hz, 1H), 7.99 (ddd, *J* = 8.2, 6.9, 1.4 Hz, 1H), 7.98 (d, *J* = 16.1 Hz, 1H), 7.94 (ddd, *J* = 8.5, 1.5, 0.8 Hz, 1H), 7.84 (ddd, *J* = 2.1, 1.8, 0.4 Hz, 1H), 7.76–7.70 (m, 3H), 7.68 (apptd, *J* = 8.3, 0.5 Hz, 1H), 7.65 (dd, *J* = 7.6, 1.8 Hz, 1H), 7.35 (ddd, *J* = 8.2, 7.3, 1.7 Hz, 1H), 7.22 (d, *J* = 16.0 Hz, 1H), 7.07 (dd, *J* = 8.4, 1.2 Hz, 1H), 7.00 (dddd, *J* = 7.9, 7.5, 0.7, 0.5 Hz, 1H), 3.96 (s, 3H); ^13^C NMR (126 MHz, (CD_3_)_2_SO): *δ* 169.42, 159.56, 157.83, 149.26, 135.63, 135.22, 135.05, 134.04, 133.97, 131.47, 131.19, 130.24, 129.34, 128.77, 128.11, 128.04, 127.74, 124.32, 124.02, 121.24, 121.16, 112.07, 55.95; HRMS (ESI) calcd for C_23_H_17_ClN_2_OS ${\left[M+H\right]}^{+}$ 405.0823; found 405.0836.

#### IS3 2-[(E)-2–(2-methoxyphenyl)ethenyl]-4–(2,5-dichlorophenylthio)quinazoline

^1^H NMR (500 MHz, (CD_3_)_2_SO): *δ* 8.20 (ddd, 1H), 8.00 (apptd, *J* = 8.9, 1.3 Hz, 1H), 8.00 (d, *J* = 2.6 Hz, 1H), 7.95 (ddd, *J* = 8.4, 1.4, 0.6 Hz, 1H), 7.82 (d, *J* = 8.7 Hz, 1H), 7.78 (dd, *J* = 8.1, 2.8 Hz, 1H), 7.76 (d, *J* = 15.9 Hz, 1H), 7.72 (ddd, *J* = 8.2, 6.9, 1.4 Hz, 1H), 7.61 (dd, *J* = 7.6, 1.8 Hz, 1H), 7.35 (ddd, *J* = 9.0, 7.3, 1.8 Hz, 1H), 7.15 (d, *J* = 16.0 Hz, 1H), 7.07 (dd, *J* = 8.4, 1.1 Hz, 1H), 6.99 (appt, *J* = 7.0 Hz, 1H), 3.88 (s, 3H); ^13^C NMR (126 MHz, (CD_3_)_2_SO): *δ* 168.04, 159.69, 157.99, 149.55, 138.27, 137.84, 135.22, 134.06, 132.52, 132.14, 132.00, 131.11, 129.06, 128.85, 128.12, 128.03, 127.81, 124.62, 124.07, 121.39, 121.24, 112.32, 56.14; HRMS (ESI) calcd for C_23_H_16_Cl_2_N_2_OS ${\left[M+H\right]}^{+}$ 439.0433; found 439.0439.

#### IS4 2-[(E)-2–(2-methoxyphenyl)ethenyl]-4–(2-bromophenylthio)quinazoline

^1^H NMR (500 MHz; (CD_3_)_2_SO): *δ* 8.21 (ddd, *J* = 8.2, 1.4, 0.7 Hz, 1H), 8.00 (ddd, *J* = 8.7, 6.9, 1.3 Hz, 1H), 7.98–7.96 (m, 1H), 7.94 (ddd, *J* = 8.4, 1.3, 0.6 Hz, 1H), 7.91–7.89 (m, 1H), 7.72 (ddd, *J* = 8.1, 6.9, 1.2 Hz, 1H), 7.71 (d, *J* = 16.2 Hz, 1H), 7.64–7.61 (m, 2H), 7.60 (dd, *J* = 7.6, 2.2 Hz, 1H), 7.34 (ddd, *J* = 8.4, 7.3, 1.7 Hz, 1H), 7.13 (d, *J* = 16.0 Hz, 1H), 7.05 (dd, *J* = 8.5, 1.1 Hz, 1H), 6.99–6.95 (m, 1H), 3.86 (s, 3H); ^13^C NMR (126 MHz, (CD_3_)_2_SO): *δ* 168.65, 159.63, 157.77, 149.33, 139.00, 135.23, 134.06, 132.49, 131.21, 131.05, 129.17, 128.75, 128.52, 128.11, 127.91, 127.56, 125.96, 124.31, 124.11, 121.28, 121.18, 112.10, 55.99. HRMS (ESI) calcd for C_23_H_17_BrN_2_OS ${\left[M+H\right]}^{+}$ 449.0318; found 449.0321.

#### IS5 2-[(E)-2–(2-methoxyphenyl)ethenyl]-4–(2-fluorophenylthio)quinazoline

^1^H NMR (500 MHz, (CD_3_)_2_CO): *δ* 8.25 (ddd, *J* = 8.2, 1.4, 0.7 Hz, 1H), 7.99 (ddd, *J* = 8.2, 6.9, 1.4 Hz, 1H), 7.94 (ddd, *J* = 8.4, 1.4, 0.6 Hz, 1H), 7.90 (d, *J* = 16.0 Hz, 1H), 7.84–7.78 (m, 1H), 7.72 (ddd, *J* = 8.2, 6.8, 1.4 Hz, 1H), 7.61 (dd, *J* = 7.7, 1.8 Hz, 1H), 7.53–7.45 (m, 2H), 7.34 (ddd, *J* = 8.2, 7.3, 1.8 Hz, 1H), 7.19 (d, *J* = 16.0 Hz, 1H), 7.05 (dd, *J* = 8.4, 1.2 Hz, 1H), 6.99 (dddd, *J* = 8.0, 7.5, 1.0, 0.5 Hz, 1H), 3.95 (s, 3H); ^13^C NMR (126 MHz, (CD_3_)_2_CO): *δ* 205.30, 168.17, 163.37 (d, *J* = 248.0 Hz), 159.87, 157.90, 149.56, 137.95, 134.23, 133.91, 132.78 (d, *J* = 8.4 Hz), 130.38, 128.69, 127.69, 127.68, 127.20, 125.15 (d, *J* = 3.8 Hz), 124.71, 123.67, 121.34, 120.63, 116.16 (d, *J* = 22.9 Hz), 114.69 (d, *J* = 18.5 Hz), 111.31, 55.03. HRMS (ESI) calcd for C_23_H_17_FN_2_OS ${\left[M+H\right]}^{+}$ 389.1118; found 389.1130.

#### IS6 2-[(E)-2–(2-methoxyphenyl)ethenyl]-4-[2-(trifluoromethyl)phenylthio]quinazoline

^1^H NMR (500 MHz, (CD_3_)_2_CO): *δ* 8.26 (ddd, *J* = 8.4, 1.4, 0.8 Hz, 1H), 8.14–8.00 (m, 2H), 7.99 (ddd, *J* = 8.2, 6.9, 1.4 Hz, 1H), 7.98–7.93 (m, 3H), 7.76 (d, *J* = 16.3 Hz, 1H), 7.73 (ddd, *J* = 8.2, 6.9, 1.5 Hz, 1H), 7.59 (dd, *J* = 7.6, 1.7 Hz, 1H), 7.34 (ddd, *J* = 8.4, 7.3, 1.8 Hz, 1H), 7.16 (d, *J* = 16.0 Hz, 1H), 7.05 (dd, *J* = 8.4, 1.1 Hz, 1H), 6.98 (dddd, *J* = 7.8, 7.5, 0.6, 0.5 Hz, 1H), 3.95 (s, 3H); ^13^C NMR (126 MHz, (CD_3_)_2_CO) *δ* 169.38, 159.82, 157.86, 149.52, 141.04, 134.31, 133.75 (q, *J* = 30.1 Hz), 133.85, 132.95 (q, *J* = 1.0 Hz), 130.59, 130.40, 128.66, 127.65, 127.61, 127.28 (q, *J* = 5.4 Hz), 127.27, 126.17 (q, *J* = 1.7 Hz), 124.66, 123.61, 123.13 (q, *J* = 273.8 Hz), 121.26, 120.62, 111.30, 55.04. HRMS (ESI) calcd for C_24_H_17_F_3_N_2_OS ${\left[M+H\right]}^{+}$ 439.1086; found 439.1101.

#### IS7 2-[(E)-2–(2-methoxyphenyl)ethenyl]-4–(3-methoxyphenylthio)quinazoline

^1^H NMR (500 MHz; (CD_3_)_2_SO): *δ* 8.18 (ddd, *J* = 8.2, 1.4, 0.6 Hz, 1H), 7.98 (ddd, *J* = 8.4, 6.9, 1.4 Hz, 1H), 7.92 (ddd, *J* = 8.5, 1.4, 0.7 Hz, 1H), 7.89 (d, *J* = 16.0 Hz, 1H), 7.70 (ddd, *J* = 8.2, 6.9, 1.4 Hz, 1H), 7.62 (dd, *J* = 7.8, 1.8 Hz, 1H), 7.52 (dd, *J* = 8.5, 7.4 Hz, 1H), 7.42–7.27 (m, 3H), 7.22 (ddd, *J* = 8.4, 2.5, 1.0 Hz, 1H), 7.17 (d, *J* = 16.0 Hz, 1H), 7.06 (dd, *J* = 8.5, 1.1 Hz, 1H), 7.00–6.96 (m, 1H), 3.85 (s, 3H), 3.81 (s, 3H); ^13^C NMR (126 MHz, (CD_3_)_2_SO): *δ* 169.85, 169.84, 160.15, 159.65, 157.82, 149.21, 135.09, 133.98, 131.18, 130.67, 128.74, 128.17, 128.00, 127.75, 127.74, 124.30, 124.02, 121.32, 121.17, 120.77, 116.59, 112.08, 55.89, 55.84. HRMS (ESI) calcd for C_24_H_20_N_2_O_2_S ${\left[M+H\right]}^{+}$ 401.1318; found 401.1318.

#### IS8 3-{2-[(E)-2–(2-methoxyphenyl)ethenyl]quinazolin-4-ylthio}phenol

^1^H NMR (500 MHz, (CD_3_)_2_SO): *δ* 9.82 (s, 1H), 8.18 (ddd, *J* = 8.2, 1.4, 0.8 Hz, 1H), 7.98 (ddd, *J* = 8.2, 6.9, 1.4 Hz, 1H), 7.92 (ddd, *J* = 8.5, 1.4, 0.6 Hz, 1H), 7.89 (d, *J* = 16.2 Hz, 1H), 7.69 (ddd, *J* = 8.2, 6.9, 1.4 Hz, 1H), 7.63 (dd, *J* = 7.8, 1.8 Hz, 1H), 7.41 (dd, *J* = 8.5, 8.0 Hz, 1H), 7.35 (ddd, *J* = 8.4, 7.3, 1.7 Hz, 1H), 7.17 (d, *J* = 16.0 Hz, 2H), 7.15 (ddd, *J* = 7.6, 1.8, 1.0 Hz, 1H), 7.12 (dd, *J* = 2.5, 1.5 Hz, 1H), 7.06 (dd, *J* = 8.2, 1.0 Hz, 1H), 7.04 (ddd, *J* = 8.2, 2.4, 0.9 Hz, 1H), 6.98 (dddd, *J* = 7.8, 7.8, 1.1, 0.6 Hz, 2H), 3.87 (s, 3H); ^13^C NMR (126 MHz, (CD_3_)_2_SO): *δ* 170.18, 159.70, 158.48, 157.89, 149.22, 135.04, 134.27, 131.13, 130.72, 128.75, 128.18, 127.96, 127.77, 127.59, 126.79, 124.39, 124.08, 122.72, 121.31, 121.14, 117.31, 112.08, 55.90. HRMS (ESI) calcd for C_23_H_18_N_2_O_2_S ${\left[M+H\right]}^{+}$ 387.1162; found 387.1161.

#### IS9 2-[(E)-2–(2-methoxyphenyl)ethenyl]-4-[4-(methylthio)phenylthio]quinazolin

^1^H NMR (500 MHz; (CD_3_)_2_SO): *δ* 8.19 (ddd, *J* = 8.2, 1.4, 0.6 Hz, 1H), 7.98 (ddd, *J* = 8.4, 6.9, 1.3 Hz, 1H), 7.92 (ddd, *J* = 8.4, 1.3, 0.6 Hz, 1H), 7.85 (d, *J* = 16.0 Hz, 1H), 7.70 (ddd, *J* = 8.2, 6.9, 1.3 Hz, 1H), 7.67–7.64 (m, 2H), 7.60 (dd, *J* = 7.6, 1.7 Hz, 1H), 7.50–7.46 (m, 2H), 7.35 (ddd, *J* = 8.4, 7.3, 1.7 Hz, 1H), 7.16 (d, *J* = 16.0 Hz, 1H), 7.06 (dd, *J* = 8.5, 1.1 Hz, 1H), 7.00–6.96 (m, 1H), 3.88 (s, 3H), 2.57 (s, 3H); ^13^C NMR (126 MHz, (CD_3_)_2_SO): *δ* 170.08, 159.62, 157.89, 149.17, 141.38, 136.69, 136.69, 135.10, 134.20, 131.18, 128.72, 128.26, 128.02, 127.86, 126.64, 126.64, 124.37, 124.04, 122.56, 121.28, 121.18, 112.12, 56.06, 14.80. HRMS (ESI) calcd for C_24_H_20_N_2_OS_2_ ${\left[M+H\right]}^{+}$ 417.1090; found 417.1092.

#### IS10 5-{2-[(E)-2–(2-methoxyphenyl)ethenyl]quinazolin-4-ylthio}-2H-1,3-benzodioxole

^1^H NMR (500 MHz; (CD_3_)_2_SO): *δ* 8.15 (appdq, *J* = 8.2, 1.7 Hz, 1H), 7.97 (ddd, *J* = 8.2, 6.8, 1.4 Hz, 1H), 7.91 (dd, *J* = 8.6, 1.6 Hz, 1H), 7.91 (d, *J* = 15.7 Hz, 1H), 7.68 (apptd, *J* = 8.3, 1.0 Hz, 1H), 7.65 (dd, *J* = 7.8, 1.8 Hz, 1H), 7.35 (ddd, *J* = 8.2, 7.2, 1.7 Hz, 1H), 7.27 (d, *J* = 1.7 Hz, 1H), 7.23 (dd, *J* = 8.0, 1.8 Hz, 1H), 7.17 (d, *J* = 7.9 Hz, 1H), 7.15 (d, *J* = 15.8 Hz, 1H), 7.06 (dd, *J* = 8.4, 1.1 Hz, 1H), 6.99 (appt, *J* = 7.5 Hz, 1H), 6.16 (s, 2H), 3.86 (s, 3H); ^13^C NMR (126 MHz, (CD_3_)_2_SO): *δ* 170.65, 159.64, 157.83, 149.39, 149.13, 148.44, 135.02, 133.92, 131.15, 131.04, 128.71, 127.93, 127.89, 127.76, 124.41, 123.98, 121.24, 121.21, 118.73, 116.29, 112.14, 109.62, 102.25, 55.93. HRMS (ESI) calcd for C_24_H_18_N_2_O_3_S ${\left[M+H\right]}^{+}$ 415.1111; found 415.1110.

#### IS11 5-{2-[(E)-2–(2-methoxyphenyl)ethenyl]quinazolin-4-ylthio}-6-bromo-2H-1,3-benzodioxole

^1^H NMR (500 MHz; (CD_3_)_2_SO): *δ* 8.17 (dd, *J* = 8.4, 1.7 Hz, 1H), 7.98 (ddd, *J* = 8.4, 6.9, 1.4 Hz, 1H), 7.93 (d, *J* = 7.9 Hz, 1H), 7.86 (d, *J* = 16.2 Hz, 1H), 7.70 (ddd, *J* = 8.2, 6.9, 1.4 Hz, 1H), 7.66 (dd, *J* = 7.8, 1.8 Hz, 1H), 7.61 (s, 1H), 7.46 (s, 1H), 7.36 (ddd, *J* = 8.9, 7.3, 1.8 Hz, 1H), 7.16 (d, *J* = 16.0 Hz, 1H), 7.07 (d, *J* = 8.5 Hz, 1H), 6.99 (appt, *J* = 7.4 Hz, 1H), 6.22 (s, 2H), 3.87 (s, 3H); ^13^C NMR (126 MHz, (CD_3_)_2_SO): *δ* 169.04, 159.66, 157.80, 150.59, 149.26, 148.09, 135.15, 133.90, 131.22, 128.74, 128.03, 127.80, 127.64, 124.37, 124.03, 123.34, 121.25, 121.23, 120.04, 117.49, 113.84, 112.16, 103.28, 56.00. HRMS (ESI) calcd for C_24_H_17_BrN_2_O_3_S ${\left[M+H\right]}^{+}$ 493.0216; found 493.0219.

#### IS12 2-[(E)-2–(2-methoxyphenyl)ethenyl]-7-chloro-4–(2-chlorophenylthio)quinazoline

^1^H NMR (500 MHz; (CD_3_)_2_CO): *δ* 8.26 (dd, *J* = 8.9, 0.6 Hz, 1H), 7.94 (dd, *J* = 2.1, 0.6 Hz, 1H), 7.90 (ddd, *J* = 7.6, 1.6, 0.3 Hz, 1H), 7.88 (d, *J* = 16.1 Hz, 1H), 7.81 (ddd, *J* = 8.1, 1.5, 0.2 Hz, 1H), 7.75 (ddd, *J* = 8.1, 7.4, 1.6 Hz, 1H), 7.70 (dd, *J* = 8.8, 2.1 Hz, 1H), 7.61 (dd, *J* = 7.8, 1.8 Hz, 1H), 7.61 (apptd, *J* = 7.6, 1.5 Hz, 1H), 7.35 (ddd, *J* = 8.4, 7.3, 1.8 Hz, 1H), 7.17 (d, *J* = 16.0 Hz, 1H), 7.05 (dd, *J* = 8.5, 0.9 Hz, 1H), 6.99 (ddd, *J* = 7.8, 1.0, 0.5 Hz, 1H), 3.94 (s, 3H); ^13^C NMR (126 MHz, (CD_3_)_2_CO): *δ* 168.55, 160.89, 158.00, 150.46, 139.74, 139.54, 138.58, 134.94, 131.92, 130.67, 130.37, 127.94, 127.85, 127.66, 127.47, 127.26, 126.55, 125.57, 124.52, 120.65, 119.91, 111.35, 55.06. HRMS (ESI) calcd for C_23_H_16_Cl_2_N_2_OS ${\left[M+H\right]}^{+}$ 439.0433; found 439.0440.

#### IS13 2-[(E)-2–(2-methoxyphenyl)ethenyl]-7-chloro-4–(3-methoxyphenylthio)quinazoline

^1^H NMR (500 MHz; (CD_3_)_2_SO): *δ* 8.21 (d, *J* = 8.7 Hz, 1H), 7.99 (d, *J* = 2.1 Hz, 1H), 7.90 (d, *J* = 16.2 Hz, 1H), 7.71 (dd, *J* = 8.7, 2.1 Hz, 1H), 7.63 (dd, *J* = 7.8, 1.7 Hz, 1H), 7.55–7.50 (m, 1H), 7.36 (ddd, *J* = 8.9, 7.2, 1.7 Hz, 1H), 7.34–7.29 (m, 2H), 7.23 (ddd, *J* = 8.4, 2.6, 1.1 Hz, 1H), 7.16 (d, *J* = 16.0 Hz, 1H), 7.06 (d, *J* = 8.4 Hz, 1H), 6.98 (t, *J* = 7.5 Hz, 0H), 3.86 (s, 3H), 3.81 (s, 3H); ^13^C NMR (126 MHz, (CD_3_)_2_SO): *δ* 170.15, 160.71, 160.28, 158.04, 150.23, 139.57, 135.00, 131.34, 130.69, 128.35, 128.23, 128.15, 127.97, 127.52, 126.13, 124.34, 121.21, 120.87, 120.05, 116.70, 112.24, 56.00, 55.92. HRMS (ESI) calcd for C_24_H_19_ClN_2_O_2_S ${\left[M+H\right]}^{+}$ 435.0929; found 435.0936.

#### IS14 2-[(E)-2–(2-methoxyphenyl)ethenyl]-7-chloro-4-(pyrid-2-ylthio)quinazoline

^1^H NMR (500 MHz, (CD_3_)_2_CO): *δ* 9.04 (dd, *J* = 4.9, 1.8 Hz, 1H), 8.59 (dd, *J* = 7.9, 1.8 Hz, 1H), 8.26 (dd, *J* = 8.9, 0.6 Hz, 1H), 8.02 (ddd, 1H), 7.99 (dd, *J* = 2.1, 0.6 Hz, 1H), 7.96 (dd, *J* = 7.8, 4.9 Hz, 1H), 7.83 (d, *J* = 16.0 Hz, 1H), 7.74 (dd, *J* = 8.8, 2.1 Hz, 1H), 7.67 (dd, *J* = 7.7, 1.8 Hz, 1H), 7.37 (ddd, *J* = 8.2, 7.3, 1.7 Hz, 1H), 7.19 (d, *J* = 16.0 Hz, 1H), 7.07 (dd, *J* = 8.4, 1.2 Hz, 1H), 7.02–6.98 (m, 1H), 6.91 (dd, *J* = 7.3, 6.4 Hz, 1H), 3.96 (s, 3H); ^13^C NMR (126 MHz, (CD_3_)_2_CO): *δ* 168.18, 160.76, 157.90, 153.65, 153.57, 150.68, 144.96, 142.06, 140.08, 134.51, 130.82, 128.08, 127.56, 126.88, 125.80, 124.31, 124.29, 120.71, 120.07, 118.06, 111.46, 111.38, 55.17. MS (ESI) calcd for C_23_H_15_ClN_4_OS ${\left[M+H\right]}^{+}$ 431.0728; found 431.2.

#### IS15 5-{2-[(E)-2–(2-methoxyphenyl)ethenyl]-7-chloroquinazolin-4-ylthio}-6-bromo-2H-1,3-benzodioxole

^1^H NMR (500 MHz; (CD_3_)_2_SO): *δ* 8.19 (dd, *J* = 8.8, 0.5 Hz, 1H), 7.97 (dd, *J* = 2.2, 0.5 Hz, 1H), 7.89 (d, *J* = 16.0 Hz, 1H), 7.69 (dd, *J* = 8.7, 2.1 Hz, 1H), 7.63 (dd, *J* = 7.8, 1.8 Hz, 1H), 7.55 (s, 1H), 7.42 (s, 1H), 7.36 (ddd, *J* = 8.4, 7.3, 1.7 Hz, 1H), 7.14 (d, *J* = 16.0 Hz, 1H), 7.08 (dd, *J* = 8.4, 1.2 Hz, 1H), 7.00 (t, *J* = 7.5 Hz, 1H), 6.21 (s, 2H), 3.89 (s, 3H); ^13^C NMR (126 MHz, (CD_3_)_2_SO): *δ* 169.32, 160.78, 158.12, 150.71, 150.33, 148.19, 139.64, 135.01, 131.31, 128.33, 128.12, 127.55, 127.53, 126.05, 124.57, 123.08, 121.29, 120.02, 120.00, 117.38, 113.82, 112.43, 103.29, 56.17. HRMS (ESI) calcd for C_24_H_16_BrClN_2_O_3_S ${\left[M+H\right]}^{+}$ 526.9826; found 526.9834.

#### IS16 2-[(E)-2–(2,4-dimethoxyphenyl)ethenyl]-4–(2-chlorophenylthio)quinazoline

^1^H NMR (500 MHz; (CD_3_)_2_CO): *δ* 8.22 (ddd, *J* = 8.2, 1.4, 0.7 Hz, 1H), 7.96 (ddd, *J* = 8.2, 6.8, 1.4 Hz, 1H), 7.91 (ddd, *J* = 8.4, 1.4, 0.7 Hz, 1H), 7.90 (ddd, *J* = 7.6, 1.6, 0.5 Hz, 1H), 7.80 (ddd, *J* = 8.1, 1.4, 0.5 Hz, 1H), 7.79 (d, *J* = 16.0 Hz, 1H), 7.74 (ddd, *J* = 8.1, 7.3, 1.6 Hz, 1H), 7.69 (ddd, *J* = 8.2, 6.9, 1.4 Hz, 1H), 7.60 (ddd, *J* = 7.5, 7.5, 1.4 Hz, 1H), 7.52 (ddd, *J* = 8.4, 0.5, 0.5 Hz, 1H), 7.07 (d, *J* = 15.9 Hz, 1H), 6.60 (d, *J* = 2.3 Hz, 1H), 6.58 (ddd, *J* = 8.3, 2.4, 0.2 Hz, 1H), 3.94 (s, 3H), 3.86 (s, 3H); ^13^C NMR (126 MHz, (CD_3_)_2_CO): *δ* 168.05, 162.19, 160.34, 159.34, 149.79, 139.75, 138.58, 134.09, 134.02, 131.58, 130.27, 128.99, 128.58, 127.76, 127.18, 126.72, 125.32, 123.58, 121.34, 117.89, 105.77, 98.32, 55.10, 54.88. HRMS (ESI) calcd for C_24_H_19_ClN_2_O_2_S ${\left[M+H\right]}^{+}$ 435.0929; found 435.0929.

#### IS17 5-{2-[(E)-2–(2,4-dimethoxyphenyl)ethenyl]quinazolin-4-ylthio}-6-bromo-2H-1,3-benzodioxole

^1^H NMR (500 MHz; (CD_3_)_2_SO): *δ* 8.15 (ddd, *J* = 8.2, 1.4, 0.6 Hz, 1H), 7.96 (ddd, *J* = 8.4, 6.9, 1.4 Hz, 1H), 7.89 (ddd, *J* = 8.4, 1.3, 0.7 Hz, 1H), 7.77 (d, *J* = 16.0 Hz, 1H), 7.67 (ddd, *J* = 8.2, 6.9, 1.3 Hz, 1H), 7.60 (s, 1H), 7.58 (d, *J* = 8.1 Hz, 1H), 7.45 (s, 1H), 7.03 (d, *J* = 16.0 Hz, 1H), 6.60 (s, 1H), 6.59 (dd, *J* = 10.0, 2.2 Hz, 1H), 6.22 (s, 2H), 3.87 (s, 3H), 3.81 (s, 3H); ^13^C NMR (126 MHz, (CD_3_)_2_SO): *δ* 168.82, 162.25, 160.08, 159.25, 150.57, 149.38, 148.08, 135.07, 134.03, 129.17, 128.61, 127.70, 125.09, 124.01, 123.34, 121.12, 120.12, 117.53, 117.36, 113.83, 106.54, 103.28, 98.88, 56.08, 55.88. HRMS (ESI) calcd for C_24_H_16_BrClN_2_O_3_S ${\left[M+H\right]}^{+}$ 523.0322; found 523.0333.

#### IS18 2-[(E)-2–(4-methoxyphenyl)ethenyl]-4–(2-chlorophenylthio)quinazoline

^1^H NMR (500 MHz, (CD_3_)_2_SO): *δ* 8.20 (ddd, *J* = 8.2, 1.3, 0.6 Hz, 1H), 7.99 (ddd, *J* = 8.4, 6.9, 1.4 Hz, 1H), 7.92 (ddd, *J* = 8.4, 1.3, 0.7 Hz, 1H), 7.90 (dd, *J* = 7.7, 1.6 Hz, 1H), 7.86 (dd, *J* = 8.0, 1.3 Hz, 1H), 7.74 (ddd, *J* = 8.1, 7.5, 1.7 Hz, 1H), 7.71 (ddd, *J* = 8.2, 7.0, 1.3 Hz, 1H), 7.60 (apptd, *J* = 7.6, 1.4 Hz, 1H), 7.46–7.40 (m, 1H), 7.25 (d, *J* = 15.9 Hz, 1H), 7.00 (d, *J* = 15.7 Hz, 1H), 7.00–6.94 (m, 2H), 3.80 (s, 3H); ^13^C NMR (126 MHz, (CD_3_)_2_SO): *δ* 168.45, 160.78, 159.46, 149.45, 139.76, 139.20, 138.94, 135.21, 132.59, 130.67, 129.58, 129.58, 128.73, 128.69, 128.41, 128.00, 126.98, 125.04, 124.16, 121.27, 114.95, 114.95, 55.74. HRMS (ESI) calcd for C_23_H_17_ClN_2_OS ${\left[M+H\right]}^{+}$ 405.0823; found 405.0836.

#### IS19 5-{(E)-2-[4–(2-chlorophenylthio)quinazolin-2-yl]ethenyl}-2H-1,3-benzodioxole

^1^H NMR (500 MHz, (CD_3_)_2_SO): *δ* 8.23 (ddd, *J* = 8.2, 1.2, 0.7 Hz, 1H), 8.01 (ddd, *J* = 8.2, 6.9, 1.4 Hz, 1H), 7.95 (ddd, *J* = 8.4, 1.3, 0.6 Hz, 1H), 7.89 (dd, *J* = 7.7, 1.6 Hz, 1H), 7.83 (dd, *J* = 8.1, 1.4 Hz, 1H), 7.74 (ddd, *J* = 8.2, 6.9, 1.4 Hz, 1H), 7.71 (ddd, *J* = 8.5, 7.7, 1.7 Hz, 1H), 7.58 (ddd, *J* = 7.6, 7.6, 1.4 Hz, 1H), 7.29 (d, *J* = 15.9 Hz, 1H), 7.21 (d, *J* = 15.9 Hz, 1H), 6.92 (dd, *J* = 8.9, 4.1 Hz, 1H), 6.88 (s, 1H), 6.88 (dd, *J* = 4.5, 0.9 Hz, 1H), 6.16 (s, 2H); ^13^C NMR (126 MHz, (CD_3_)_2_SO): *δ* 168.69, 159.06, 149.33, 148.04, 146.14, 139.63, 139.01, 135.32, 133.26, 132.72, 130.77, 129.72, 128.92, 128.71, 128.39, 126.68, 124.18, 122.48, 121.64, 121.40, 118.37, 109.45, 101.89. HRMS (ESI) calcd for C_23_H_15_ClN_2_O_2_S ${\left[M+H\right]}^{+}$ 419.0616; found 419.0631.

### Docking studies

Docking to protein kinase structures was performed using Glide XP (eXtra Precision) in Schrodinger Suite 2019–2. The poses (protein-ligand complex) obtained with Glide XP were further refined in a Variable-Dielectric Generalised Born (VSGB) solvent model that accounts for local protein flexibility. The structures were prepared for docking using Protein Preparation Wizard. Conformers of ligands were generated using LigPrep and Epik. The protonation states of proteins and ligands were calculated at pH 7.4. Protein-ligand interactions were analysed using the protein-ligand interaction tool in Schroedinger Maestro 12.

### Tyrosine kinase assay

To determine the inhibition of non-receptor tyrosine kinases, assays were performed using the kinase selectivity system TK-2 and the ADP-Glo Kinase Assay (both from Promega). The protocol was previously developed by our group and described in reports^12^^,^^36^. Experiments were performed at least four times. Data are expressed as percentage of tyrosine kinase inhibitory activity after treatment with the derivatives tested.

### Pharmacological synergy on the ABL enzyme model

Inhibition of ABL with a combination of IS1 derivative and ABL inhibitors: imatinib, dasatinib and GNF-2 were measured with an ABL1 Kinase Assay (Promega) according to a methodology that was similar to the one described above. Synergy experiments were performed analogously to our previous reports^36^^,^^65^. Synergy was calculated by the Chou-Talay method (CompuSyn software)^66^^,^^67^. Experiments were performed at least three times.

### Cell culture

The wild type human colon carcinoma cell line - HCT 116 (p53^+/+^), the human alveolar basal epithelial cell line - A549 and the human breast carcinoma cell line - MCF-7 were purchased from ATCC. The human colon cancer cell line HCT 116 with a p53 deletion (p53^-/-^) was kindly provided by Prof. M. Rusin from the Maria Sklodowska-Curie Memorial Cancer Centre and Institute of Oncology in Gliwice, Poland. The glioblastoma cell line U-251 was kindly provided by Prof. G. Kramer-Marek from the Institute of Cancer Research in London, United Kingdom. The human suspension chronic myeloids leukaemia cell line K562 and the pancreatic ductal adenocarcinoma cell line PANC-1 were purchased from Merck. The normal human dermal fibroblasts cell line NHDF were obtained from PromoCell. All adherent cancer cell lines were cultured in Dulbecco’s Modified Eagle’s medium (DMEM) supplemented with 10% heat-inactivated foetal bovine serum – FBS (all from Merck). The suspension K562 cell line was grown in RPMI-1640 medium (Merck) containing 10% heat-inactivated FBS. The DMEM for NHDF cells was supplemented with 15% non-inactivated FBS. Each complete medium contained a mixture of two antibiotics - penicillin and streptomycin (1% v/v; Gibco). Cell lines were cultured at 37 °C with 5% CO_2_ humidified atmosphere.

### Cytotoxicity studies

Cells were seeded in 96-well plates (Nunc) at a density of 5,000 cells per well (K562, HCT 116, MCF-7, U-251, A549, PANC-1) or 4,000 cell per well (NHDF) and incubated under standard condition at 37 °C for 24 h. The assay was carried out after 72 h of incubation with different concentrations of the tested compounds. Then DMEM without phenol red was added to each well with CellTiter 96^®^AQueous One Solution-MTS (Promega) solution and incubated for 1 h or 3 h (PANC-1) at 37 °C. The optical densities of the samples were measured at 490 nm using a multi-plate reader (Synergy 4, BioTek). The obtained results were compared with the control and estimated as inhibitory concentration values (IC_50_) using GraphPad Prism 8.0. Each compound was tested in triplicate in a single experiment and each experiment was performed three or four times.

### Cell cycle assay

K562 cells were seeded in 3 cm Petri dishes (Nunc) at a density of 150,000 cells per well and incubated under standard conditions at 37 °C for 24 h. The medium was then removed and freshly prepared solutions of the tested compounds: IS1 (50 µM, 30 µM), IS8 (12 µM, 6 µM), IS10 (20 µM, 10 µM), imatinib (2 µM, 1 µM), GNF-2 (2 µM, 1 µM) and axitinib (2 µM, 1 µM) were added. After 16 h of treatment, assays were performed using a Muse Cell-Cycle Kit (Millipore) according to the supplier’s instructions. Briefly, leukaemia cells were collected, washed in cold PBS, and centrifuged. Cells were then fixed in ice-cold 70% ethanol and stored at −20 °C overnight. The next day, leukaemia cells were washed with cold PBS, centrifuged and resuspended in Muse™ Cell Cycle Reagent. Samples were incubated for 30 min at room temperature in the dark. After staining, the levels of cellular subpopulations in each cell cycle phase were determined using a Muse Cell Analyser (Millipore). Experiments were performed at least four times.

### Annexin V binding assay

Leukaemia cells were seeded as described above in Section "Cell cycle assay". The following day, the medium was removed and solutions of the tested compounds: IS1 (50 µM, 30 µM), IS8 (12 µM, 6 µM), IS10 (20 µM, 10 µM) and axitinib (2 µM, 1 µM) were added. After 30 h incubation, assays were carried out using the FITC Annexin V Apoptosis Detection kit with 7-AAD (Bio-Legend) according to the manufacturer’s instructions. Briefly, the cells were collected, washed with cold PBS and centrifuged. Cells were then resuspended in Annexin V Binding Buffer and incubated for 15 min at room temperature in the dark with FITC Annexin V and 7-AAD Viability Staining Solution. After staining, the number of events for live, early apoptotic and late apoptotic cells was determined using a Muse Cell Analyser. Experiments were performed at least four times.

### Analysis of mRNA expression

Total RNA was isolated from leukaemia cells after 16 h exposure to IS1 (50 µM), IS8 (12 µM), IS10 (20 µM), imatinib (2 µM) and GNF-2 (2 µM) using a TRIzol Reagent procedure (Ambion). Reverse transcription was carried out with 1 μg total RNA using a ProtoScript M-MuLV First Strand cDNA Synthesis Kit (New England BioLabs). The RT-qPCR was performed using a CTX96 Real-Time PCR Detection System (Biorad) in a 10 μL reaction volume containing Luna Universal qPCR Master Mix (New England BioLabs), specific primer pair mix and cDNA. The PCR reaction was performed as follows: initial denaturation at 95 °C for 60 s; followed by 40 cycles of denaturation at 95 °C, 15 s; annealing (primer-specific temperature for 30 s) and extension at 72 °C for 30 s. Melting curve analysis was used to determine the specific PCR products. The results were analysed based on comparison of the expression of the target genes with the reference gene – *HPRT1* using the 2^−^*^ΔΔ^*^CT^ method. Experiments were performed at least four times. All primers were purchased from Merck and are listed in Table S2.

### Immunoblotting

Prior to the experiment, K562 cells were seeded at a density of 500,000 cells per well in 3 cm Petri dishes (Nunc). The following day, the medium was replaced with the solution of the tested compounds: IS1 (50 µM, 30 µM), IS8 (25 µM, 12 µM, 6 µM), IS10 (40 µM, 20 µM, 10 µM), imatinib, and GNF-2 (both: 1 µM). After 16 h of exposure, cells were collected, centrifuged, and lysed on ice in complete RIPA buffer containing Halt Protease Inhibitor Cocktail, Halt Phosphatase Inhibitor Cocktail and 0.5 M EDTA (all from Thermo Scientific). Protein quantification was measured using a BCA Protein Assay Kit (Thermo Scientific) according to the manufacturer’s protocol. Equal amounts of the proteins were electrophoresed on SDS-page gels and transferred to nitrocellulose membranes. After blocking in 5% non-fat milk prepared in TTBS (Tris buffered saline with Tween 20), membranes were incubated overnight at 4 °C with specific primary antibodies (all from Cell Signalling) at a dilution of 1:1000 for c-ABL, phospho-c-ABL, Stat5, phospho-Stat5, cyclin E1, p27, PARP, AIF, BID, Src, Fyn, Akt, phospho-Akt, and at a dilution of 1:2000 for reference proteins (vinculin, β-actin and GAPDH). The next day, membranes were washed and incubated with horseradish peroxidase (HRP)-conjugated secondary antibodies for 1 h at room temperature. Chemiluminescence signals were recorded after staining with SuperSignal™ West Pico Chemiluminescent Substrate (Thermo Scientific) using a ChemiDoc™ XRS + System (BioRad). Experiments were performed four to five times. Densitometric analysis was performed using ImageJ software (Wayne Rasband, National Institutes of Health, USA).

### Statistical analysis

Results are expressed as mean ± standard deviation (SD) of all independent experiments performed. Statistical analysis for cell cycle, apoptosis, mRNA and immunoblotting experiments was performed using the one- or two-way test ANOVA with a Dunnett post-hoc test. Statistical analysis of pharmacological synergy was estimated using a two-way ANOVA with Tukey’s post-hoc test. A *p* values of 0.05 or less was considered statistically significant.

The supporting information contains the ^1^H and ^13^C NMR and HRMS spectra for all of the synthesised compounds, the selectivity indexes of the active styrylquinazolines and the molecular docking results of the reference inhibitors to ABL kinase in different conformational states.

## Supplementary Material

Supplemental Material
