# Supplementary material for: Styrylquinazoline derivatives as ABL inhibitors selective for different DFG orientations
Source: J Enzyme Inhib Med Chem. 2023 Apr 18;38(1):2201410. doi: 10.1080/14756366.2023.2201410 (PMC10120462; doi:10.1080/14756366.2023.2201410)
Supplement: Supplemental Material [file IENZ_A_2201410_SM6061.pdf]

Styrylquinazoline derivatives as ABL inhibitors selective for different DFG orientations

Katarzyna Malarz[1*†], Jacek Mularski[2†], Marcin Pacholczyk[3], Robert Musiol[2]

[1] Institute of Physics, University of Silesia in Katowice, 75 Pułku Piechoty 1a, 41-500 Chorzów, Poland
[2] Institute of Chemistry, University of Silesia in Katowice, 75 Pułku Piechoty 1a, 41-500 Chorzów, Poland
[3] Silesian University of Technology, Department of Systems Biology and Engineering, Akademicka 16, 44-100 Gliwice, Poland

[†]These authors contributed equally.
Corresponding author: katarzyna.malarz@us.edu.pl

1. Chemistry

Fourier transform nuclear magnetic resonance spectra of the sample solutions were obtained using a Bruker Ascend 500 for ^{1}H (500 MHz), and chemical shifts are reported in δ units (parts per million) relative to tetramethylsilane and the peaks of the residual solvent are used as reference. High-resolution mass spectra were measured using a DionexUltiMate® 3000 high-performance liquid chromatograph (Thermo Fisher Scientific, West Palm Beach, FL, USA) coupled to an LTQ Orbitrap XL™ Hybrid Ion Trap-Orbitrap Fourier Transform Mass Spectrometer (Thermo Fisher Scientific) with injection into HESI II in positive or negative modes.

1.1. ^{1}H NMR spectra of target compounds

For better visibility of overlapping multiplets, in some cases color was used (MestReNova – simulate multiplet functionality).

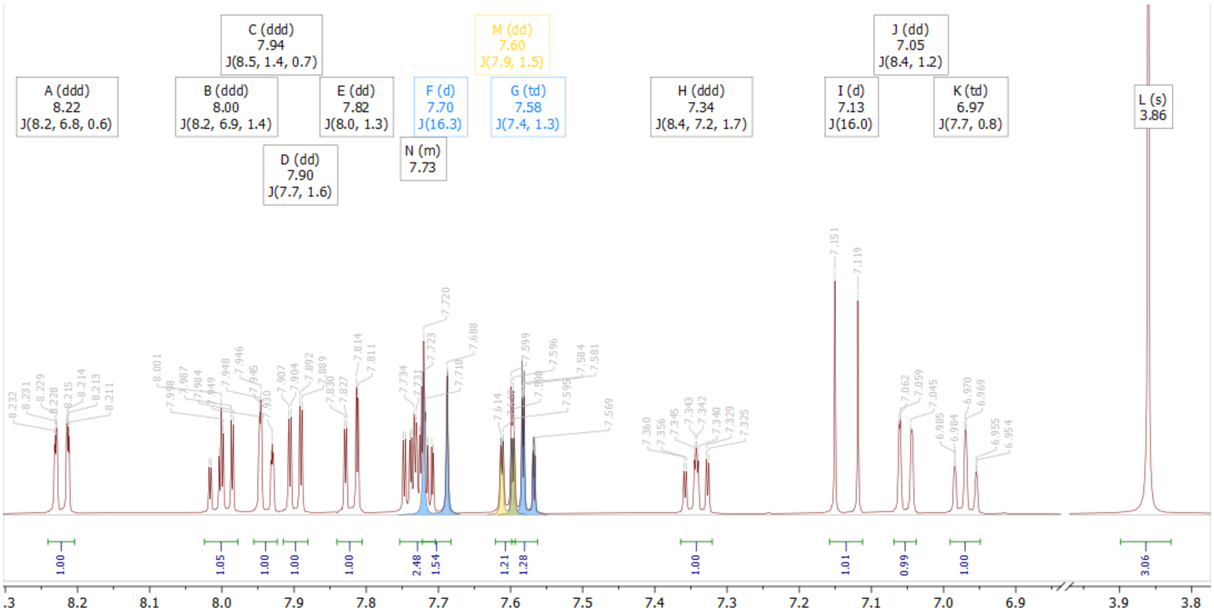

Fig. S1 ^{1}H NMR plot of **IS1**.

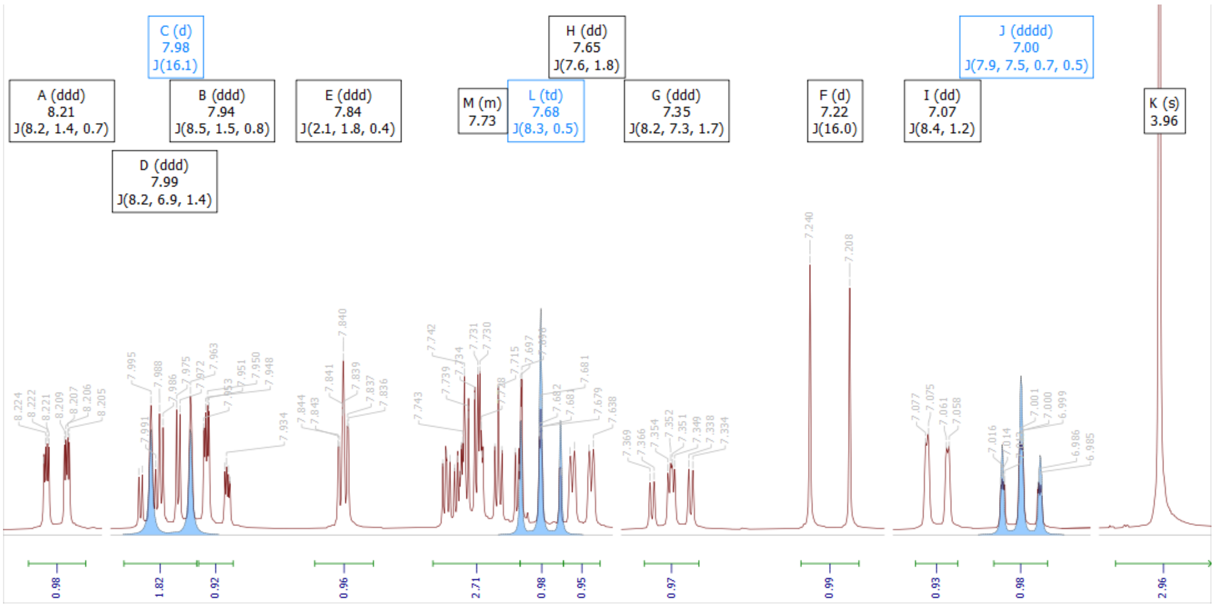

Fig. S2 ^{1}H NMR plot of **IS2**.

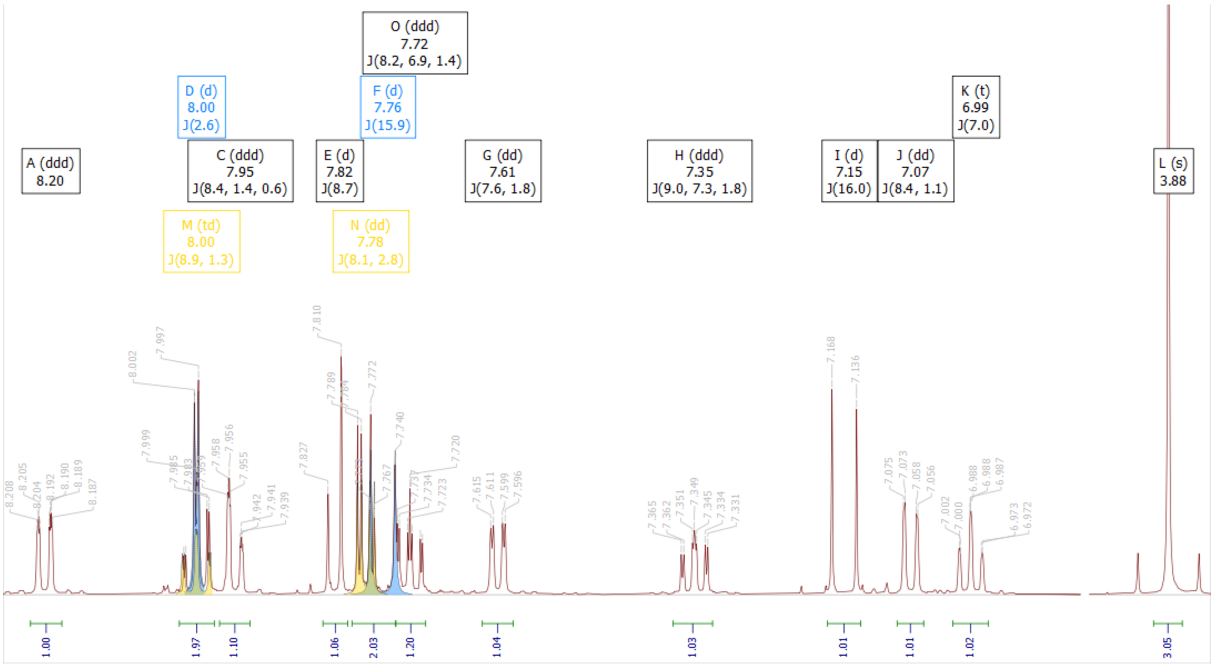

Fig. S3 ^{1}H NMR plot of **IS3**.

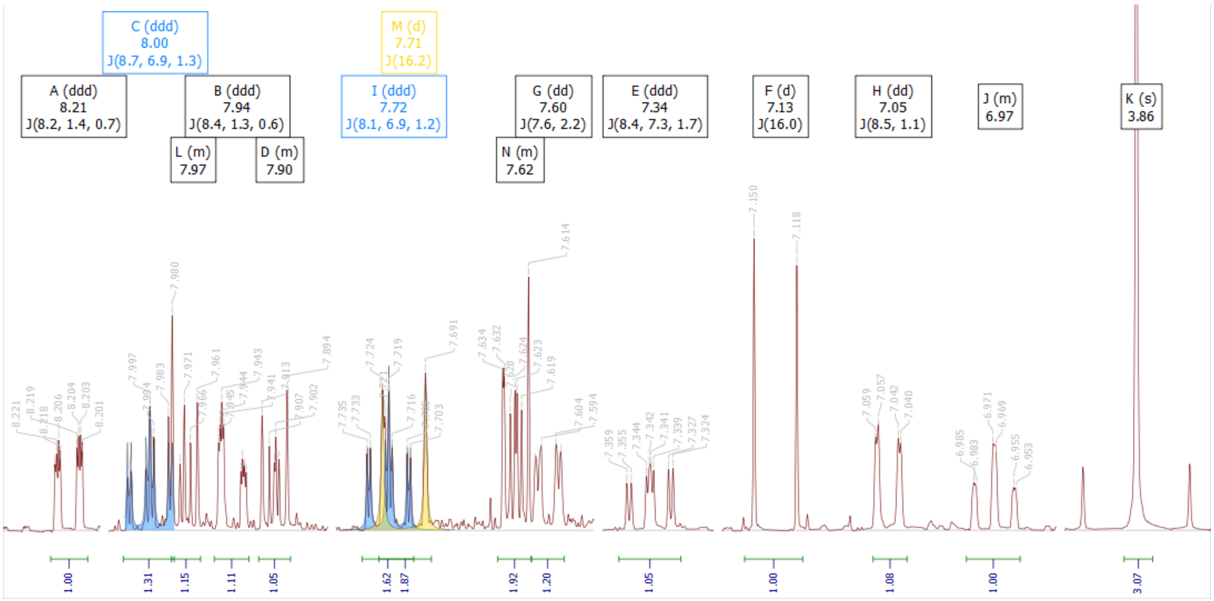

Fig. S4 ^{1}H NMR plot of **IS4**.

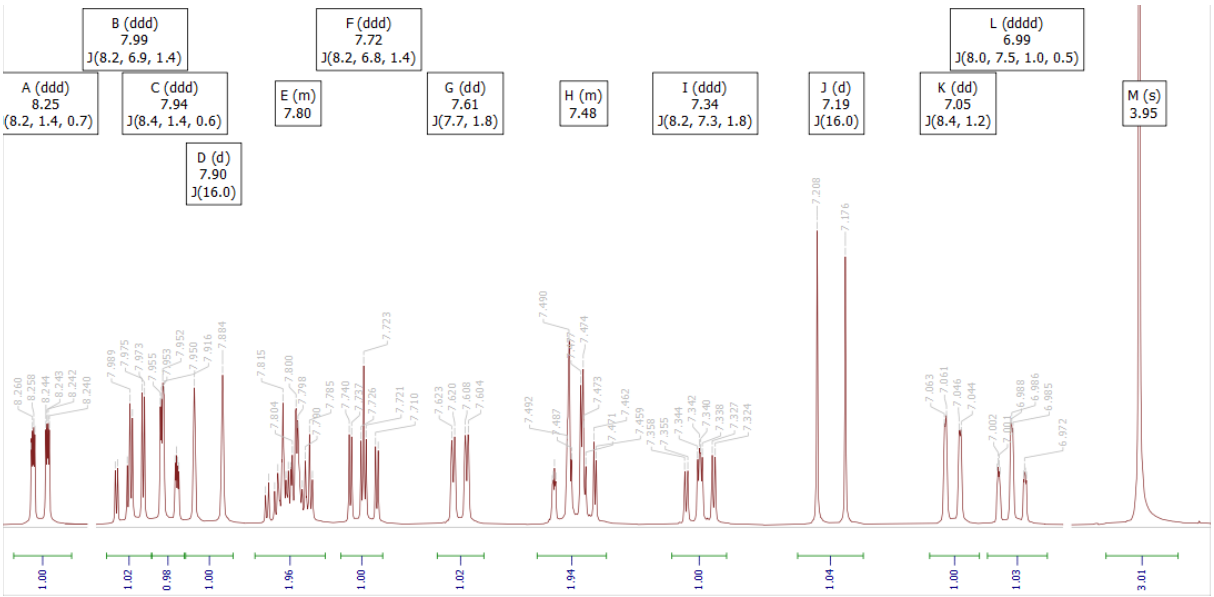

Fig. S5 ^{1}H NMR plot of **IS5**.

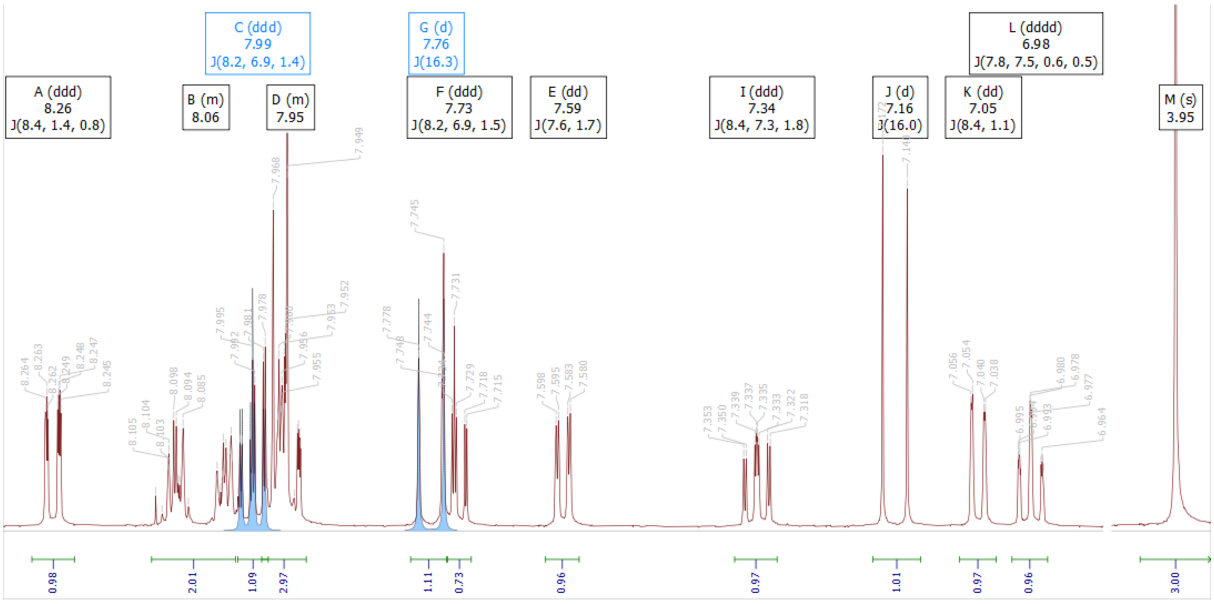

Fig. S6 ^{1}H NMR plot of **IS6**.

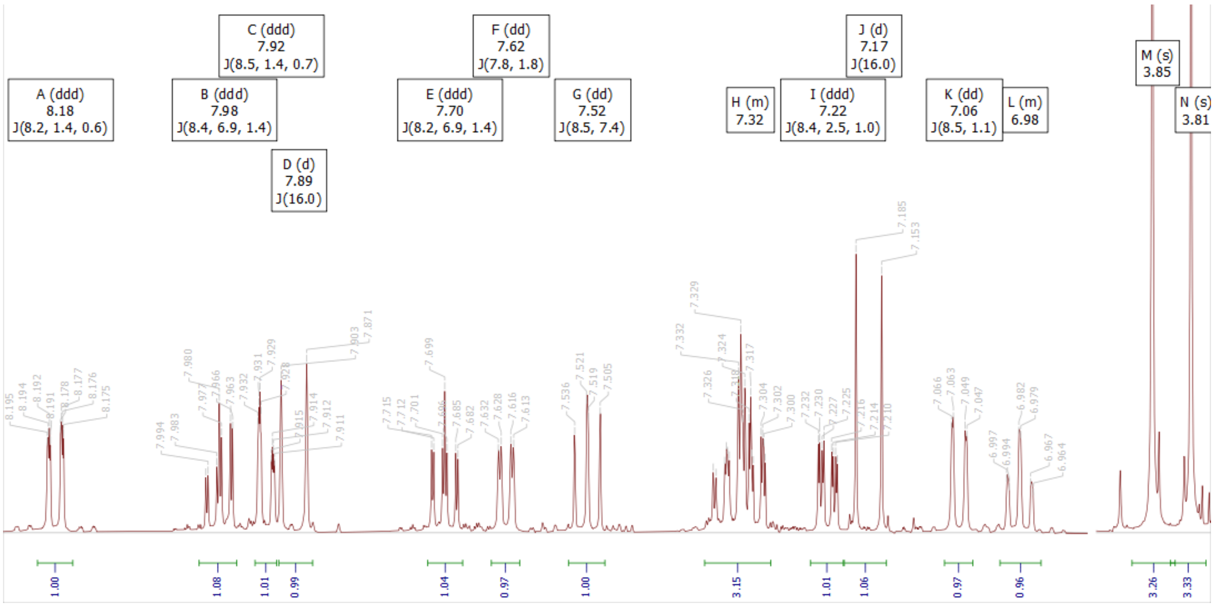

Fig. S7 ^{1}H NMR plot of **IS7**.

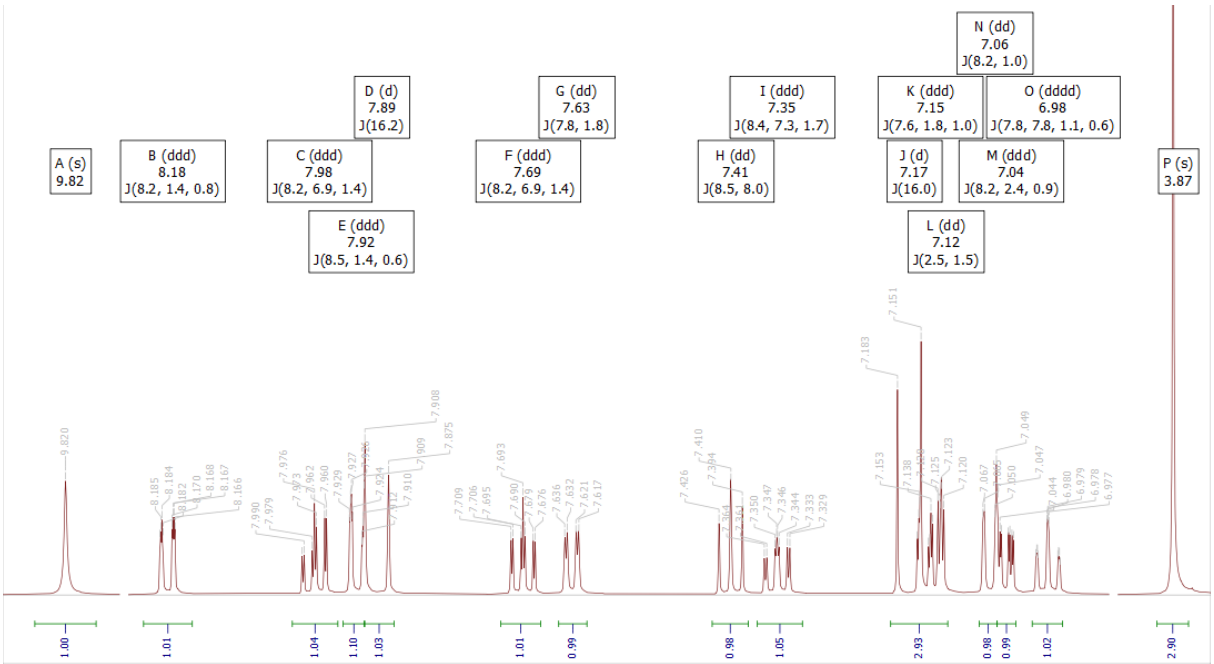

Fig. S8 ^{1}H NMR plot of **IS8**.

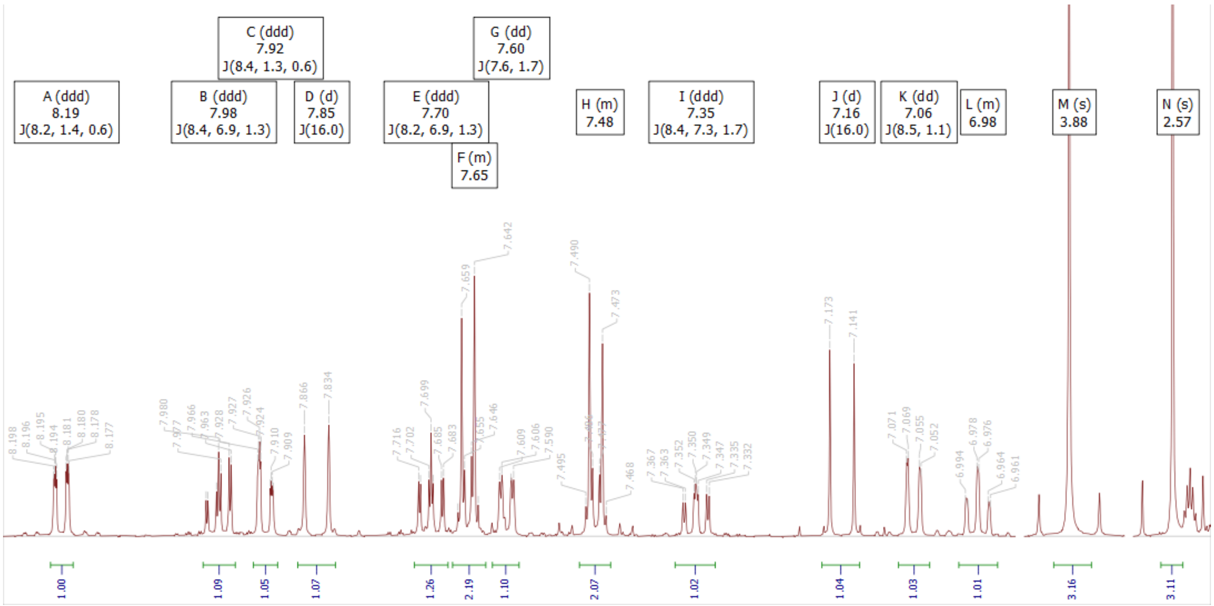

Fig. S9 ^{1}H NMR plot of **IS9**.

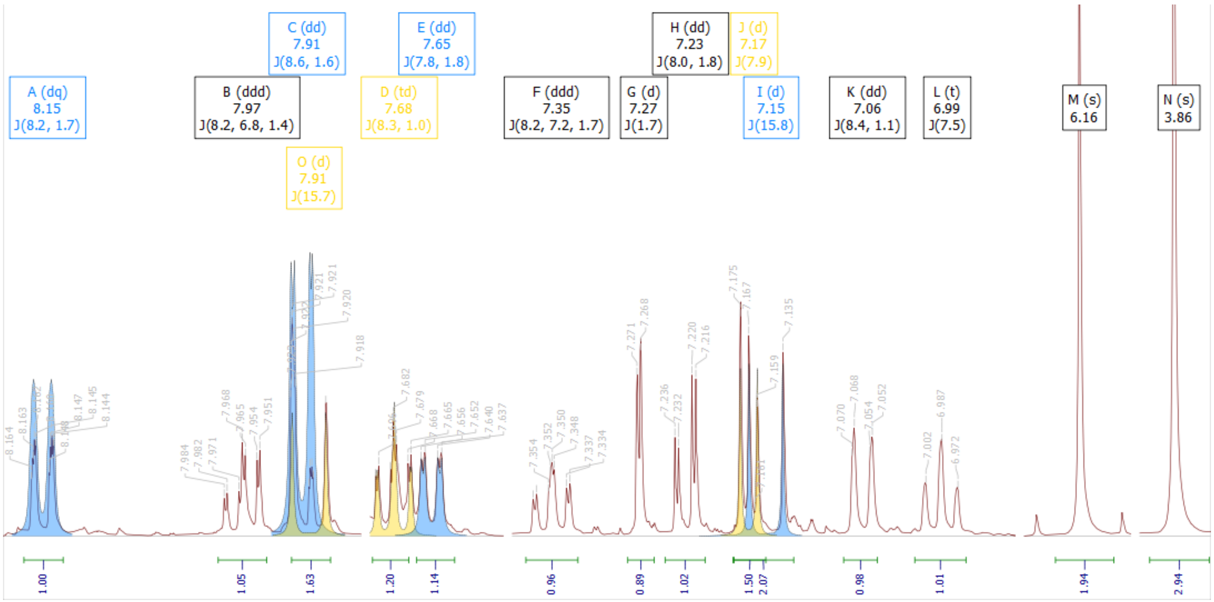

Fig. S10 ^{1}H NMR plot of **IS10**.

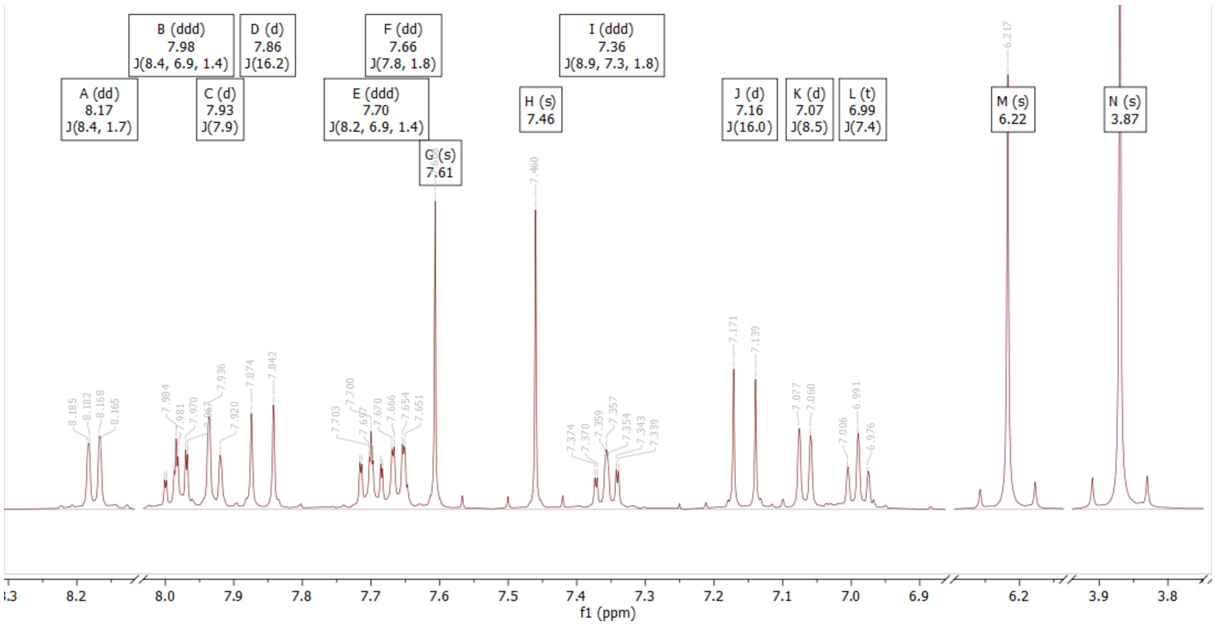

Fig. S11 ^{1}H NMR plot of **IS11**.

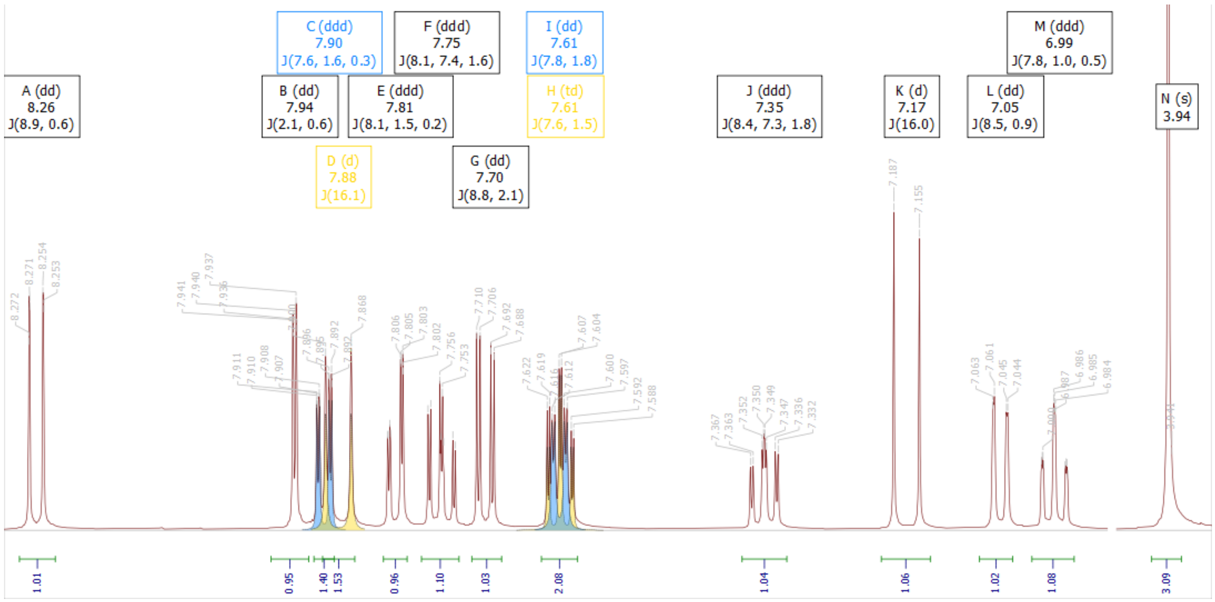

Fig. S12 ^{1}H NMR plot of **IS12**.

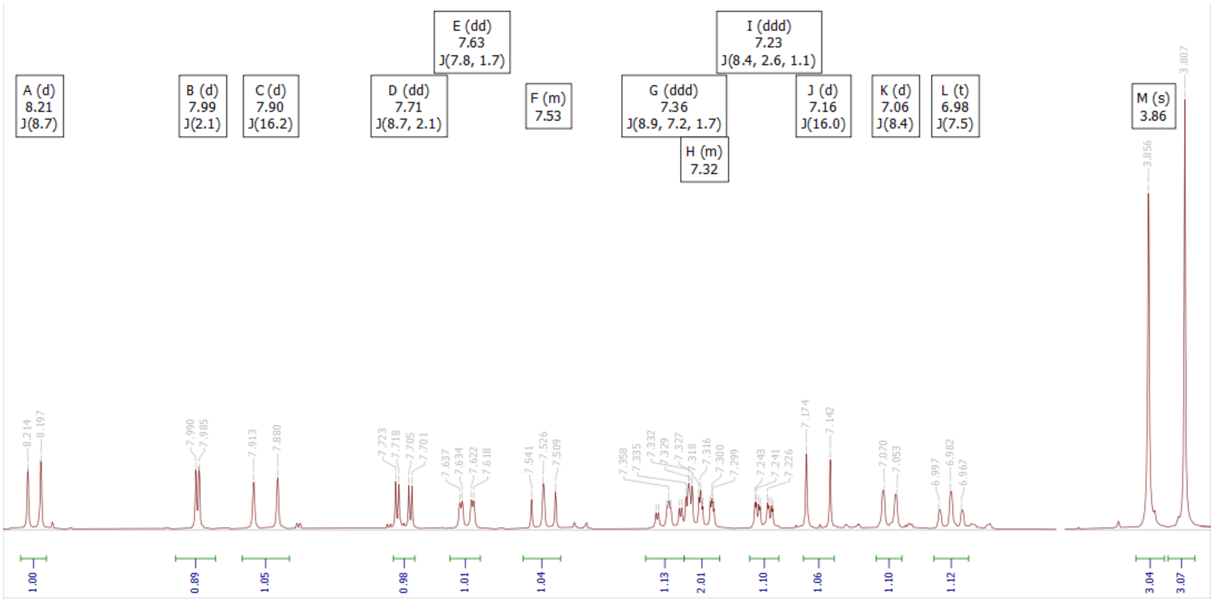

Fig. S13 ^{1}H NMR plot of **IS13**.

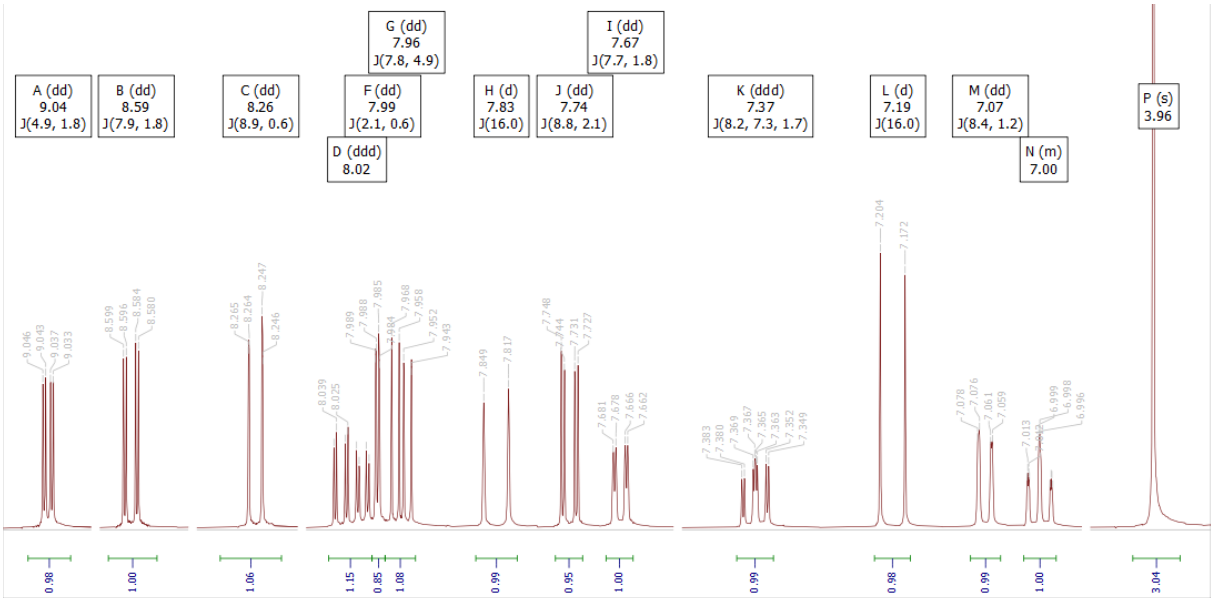

Fig. S14 ^{1}H NMR plot of **IS14**.

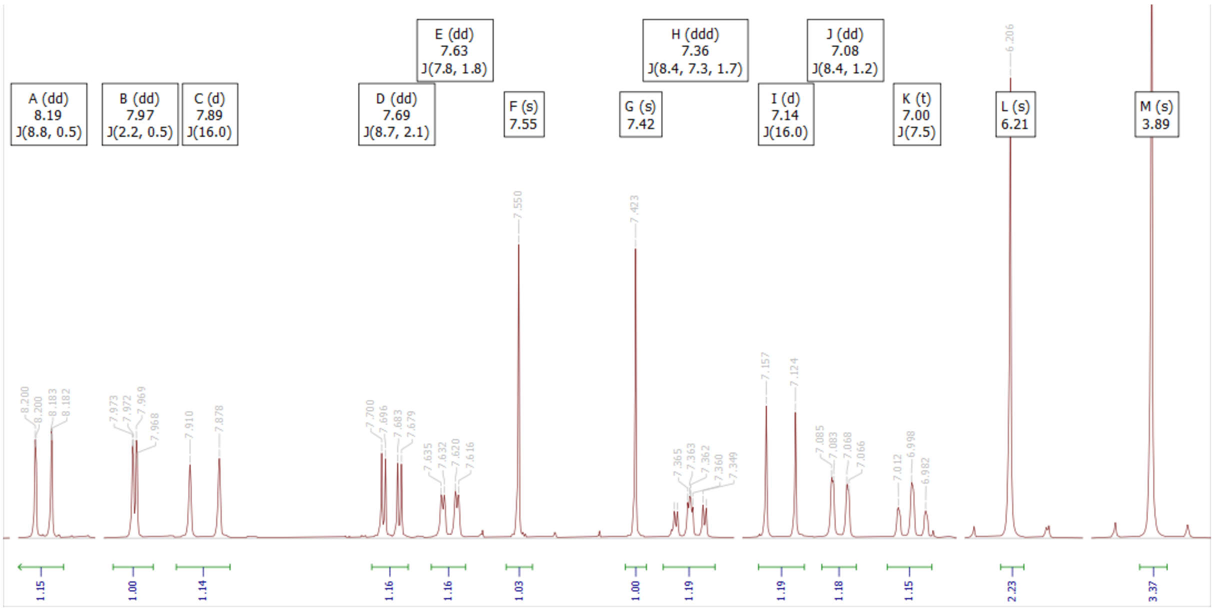

Fig. S15 ^{1}H NMR plot of **IS15**.

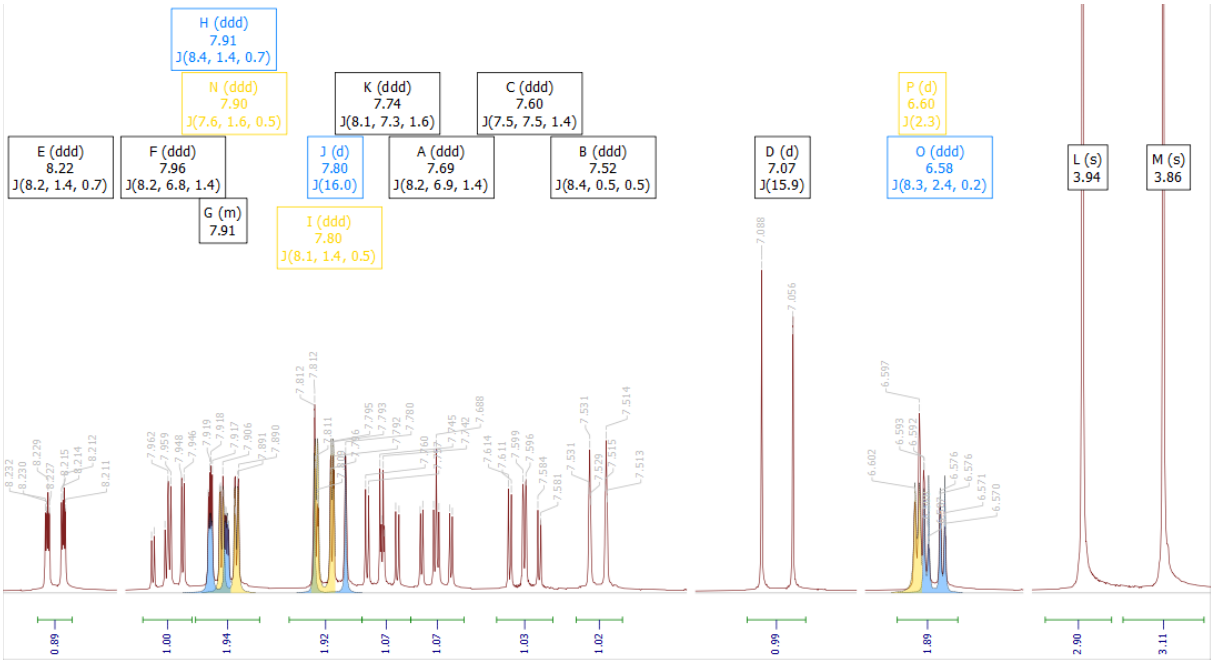

Fig. S16 ^{1}H NMR plot of **IS16**.

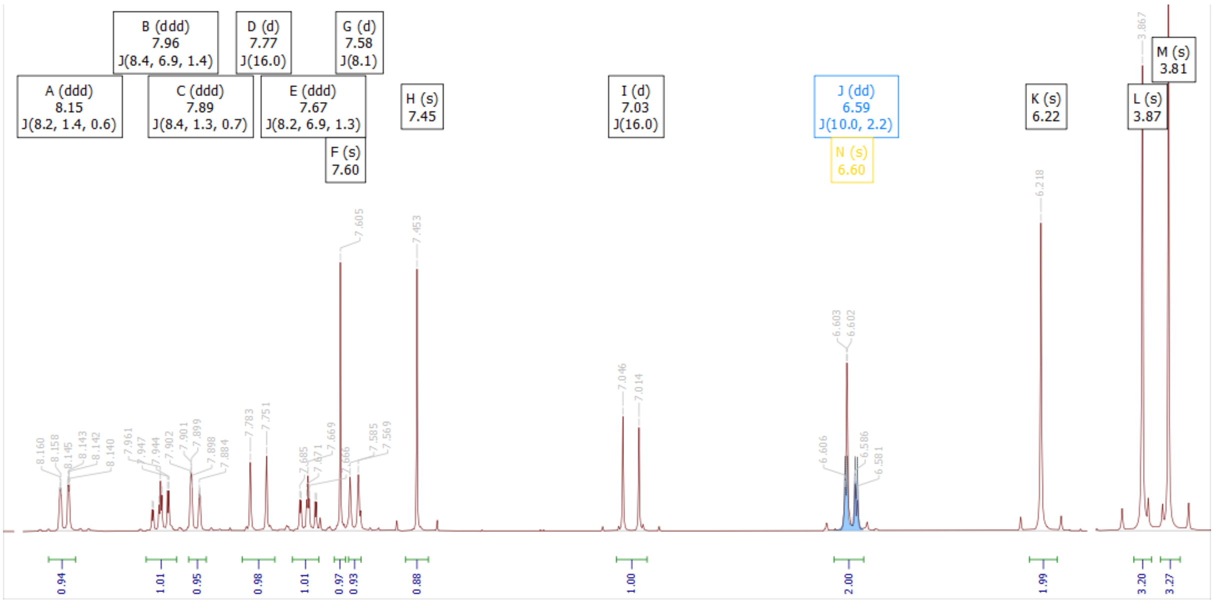

Fig. S17 ^{1}H NMR plot of **IS17**.

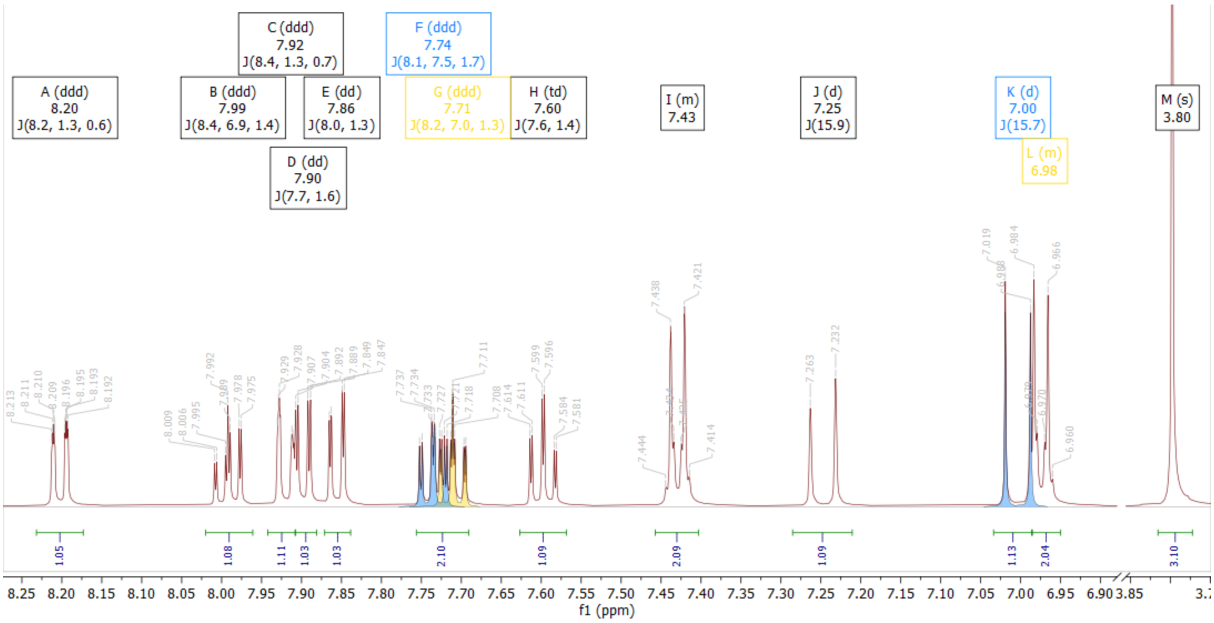

Fig. S18 ^{1}H NMR plot of **IS18**.

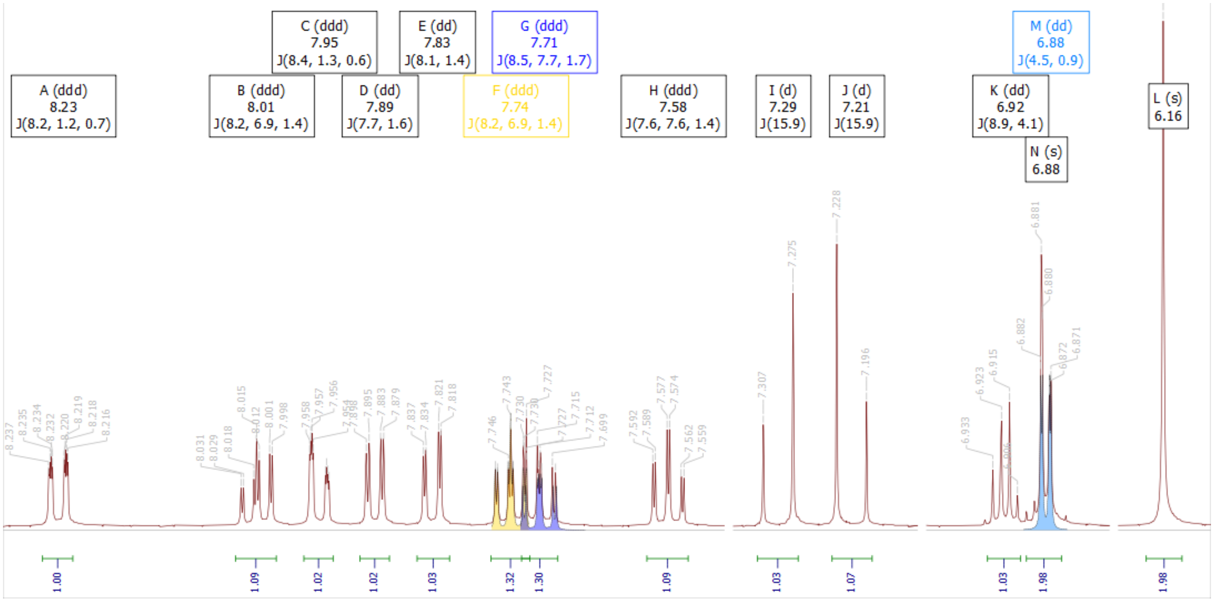

Fig. S19 ^{1}H NMR plot of **IS19**.

1.2.^{13}C NMR spectra of target compounds

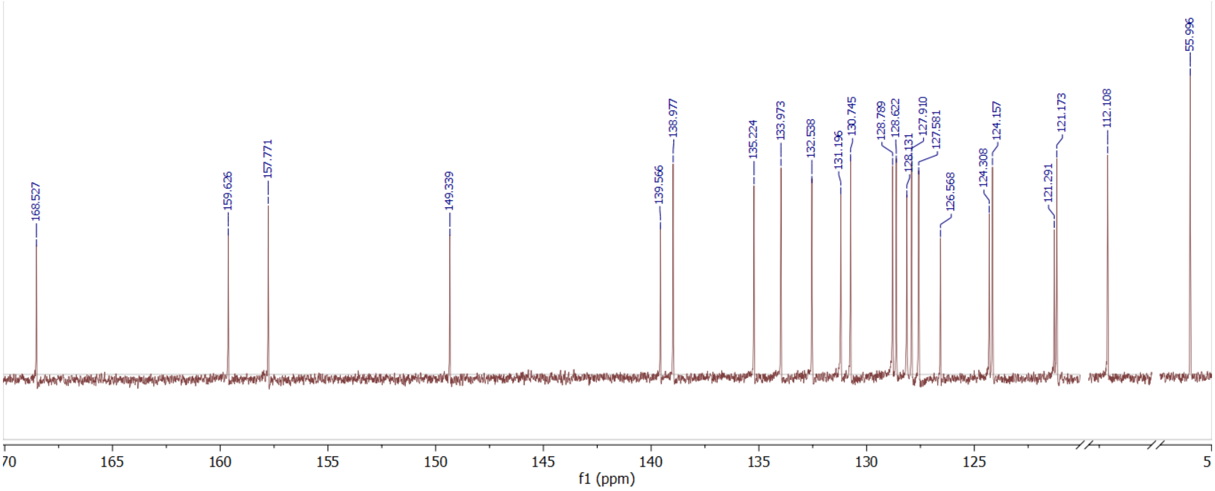

Fig. S20 ^{13}C NMR plot of **IS1**.

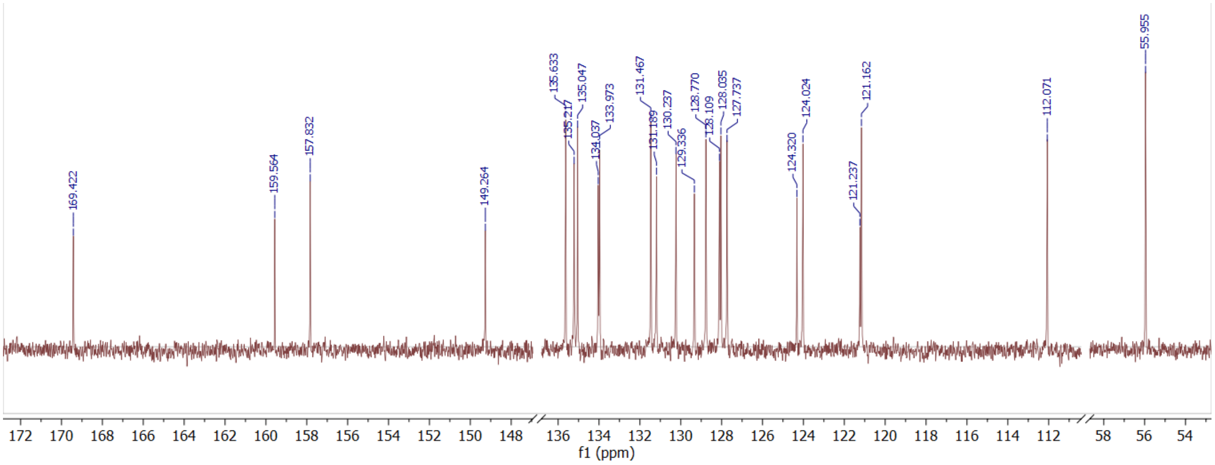

Fig. S21 ^{13}C NMR plot of **IS2**.

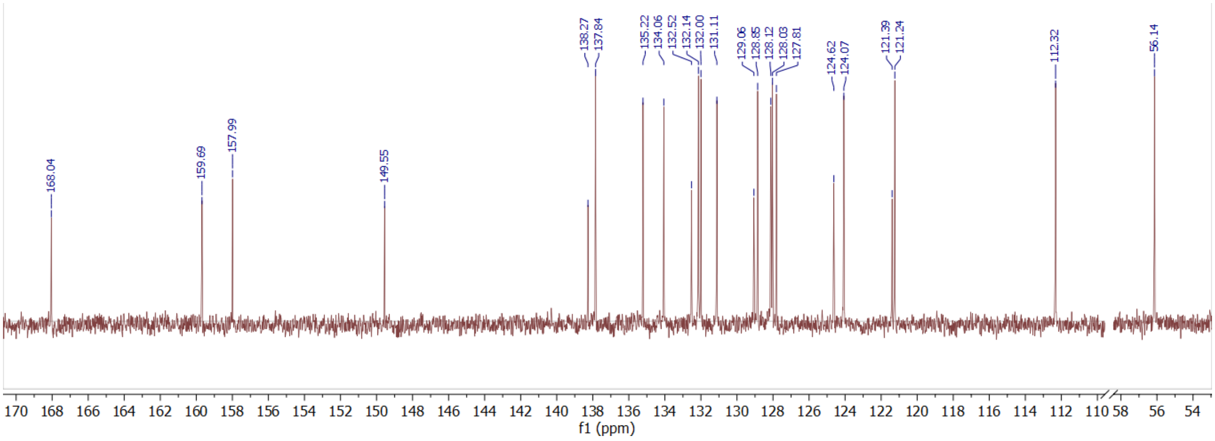

Fig. S22 ^{13}C NMR plot of **IS3**.

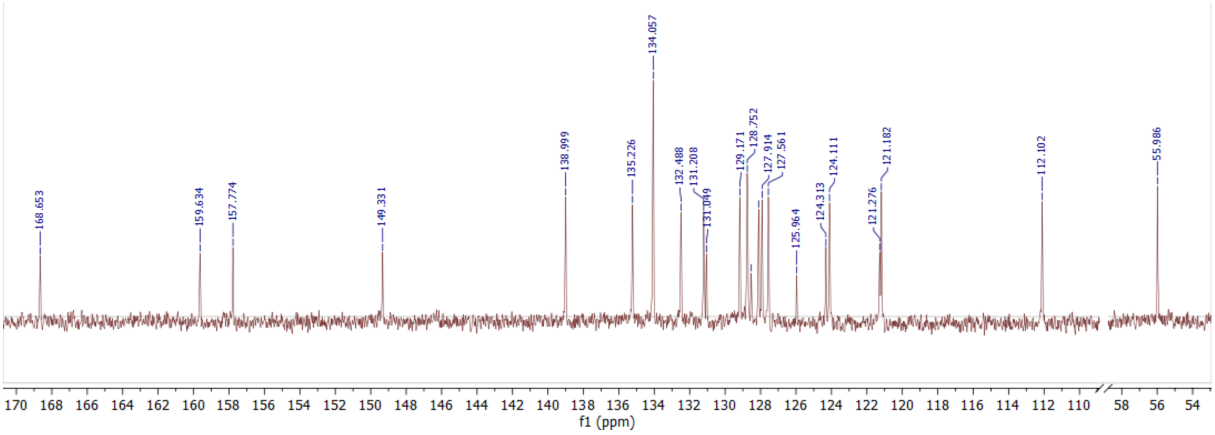

Fig. S23 ^{13}C NMR plot of **IS4**.

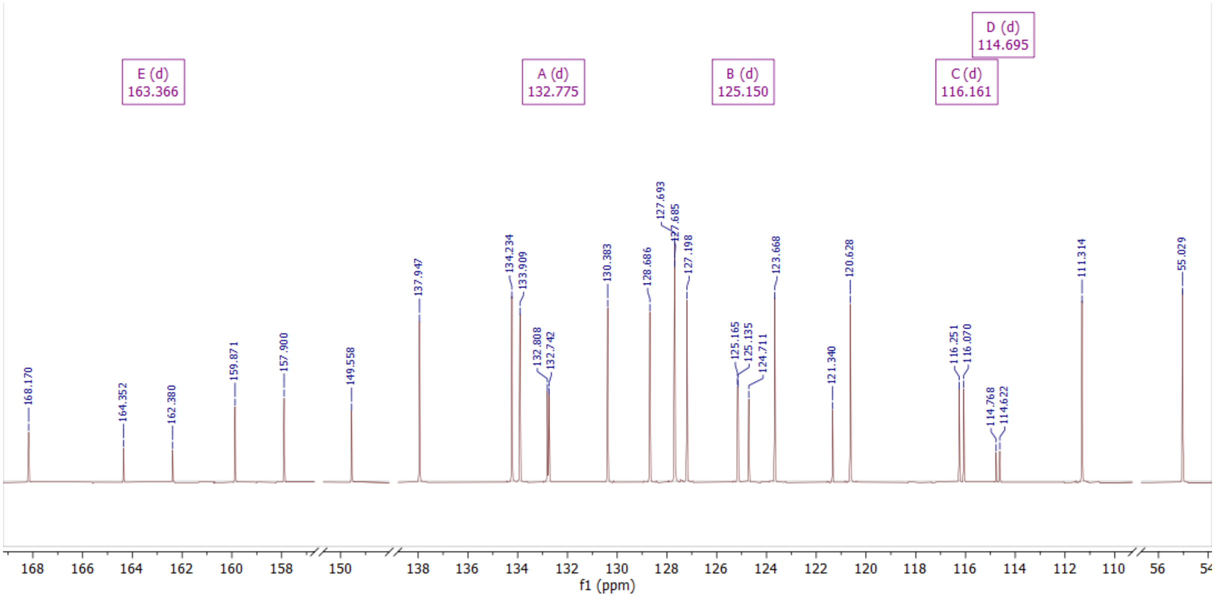

Fig. S24 ^{13}C NMR plot of **IS5**.

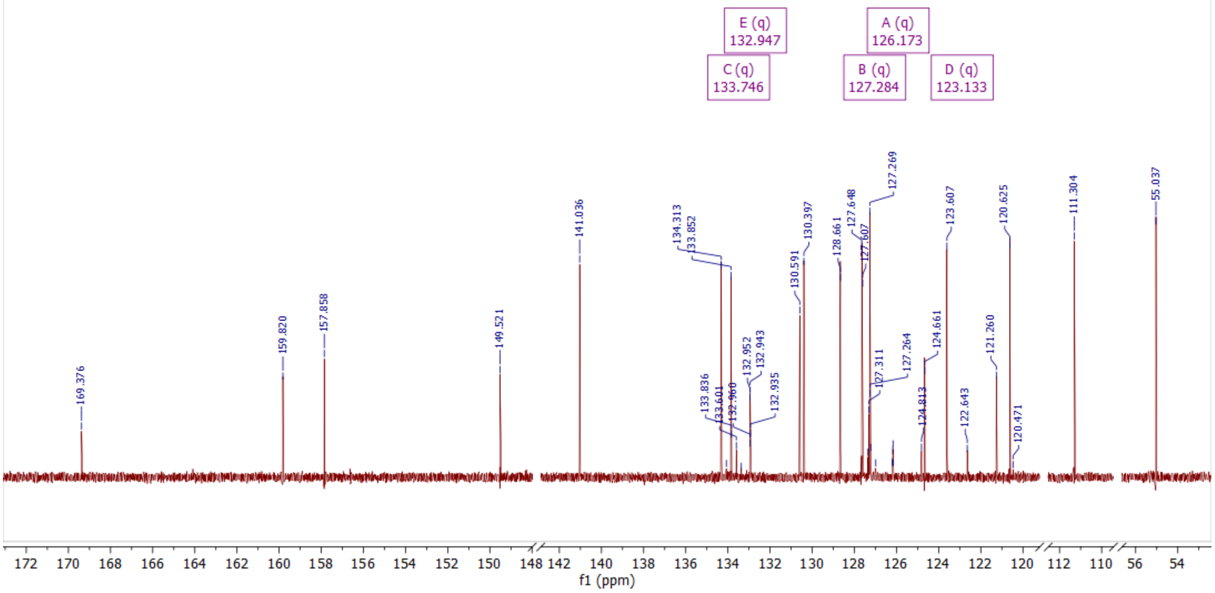

Fig. S25 ^{13}C NMR plot of **IS6**.

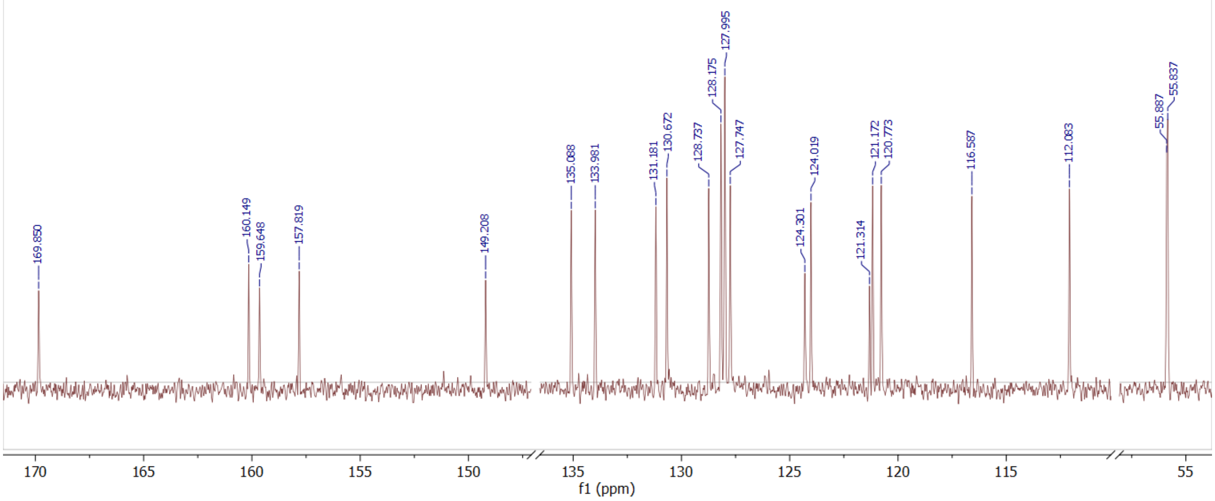

Fig. S26 ^{13}C NMR plot of **IS7**.

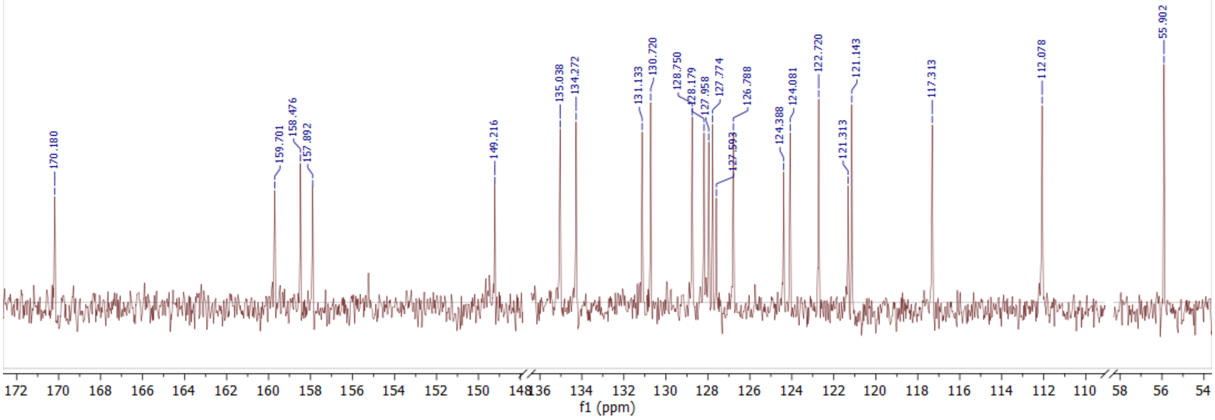

Fig. S27 ^{13}C NMR plot of **IS8**.

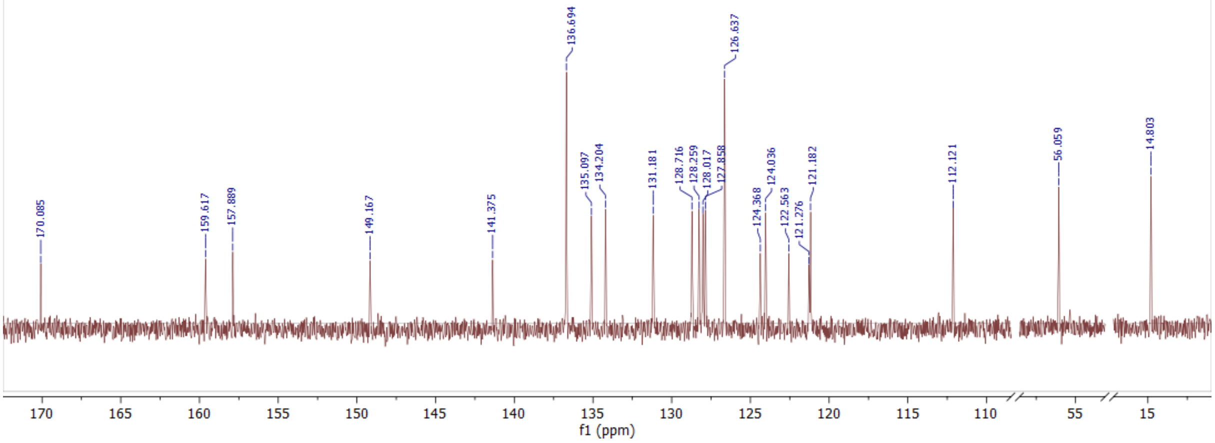

Fig. S28 ^{13}C NMR plot of **IS9**.

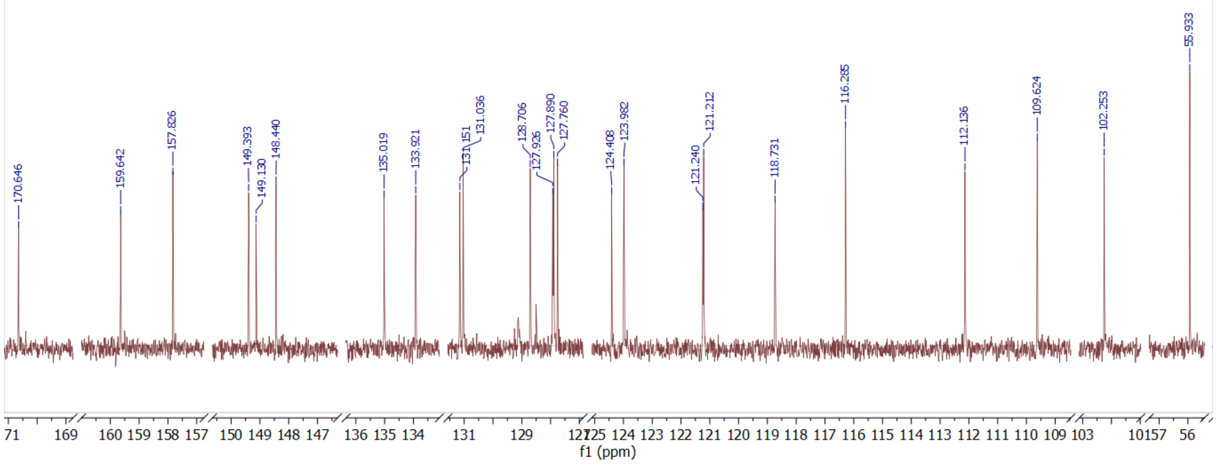

Fig. S29 ^{13}C NMR plot of **IS10**.

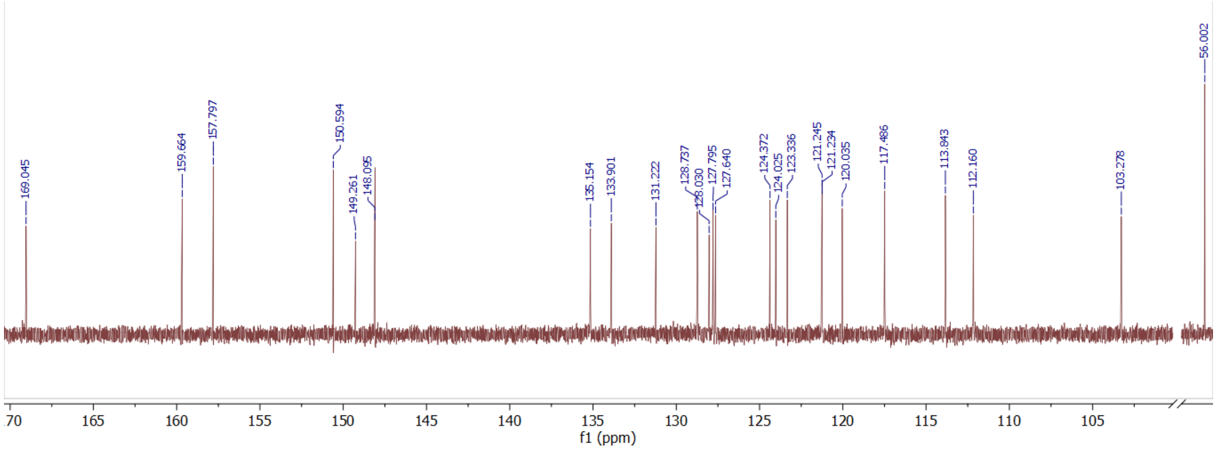

Fig. S30 ^{13}C NMR plot of **IS11**.

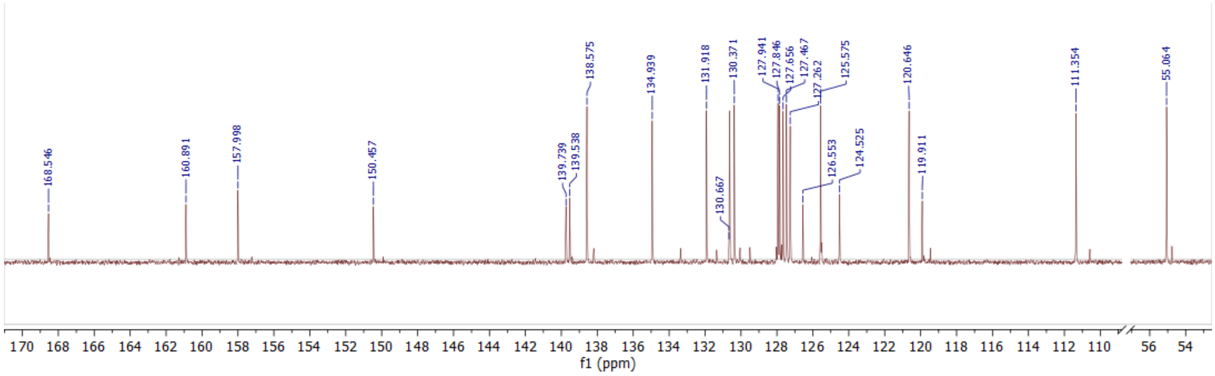

Fig. S31 ^{13}C NMR plot of **IS12**.

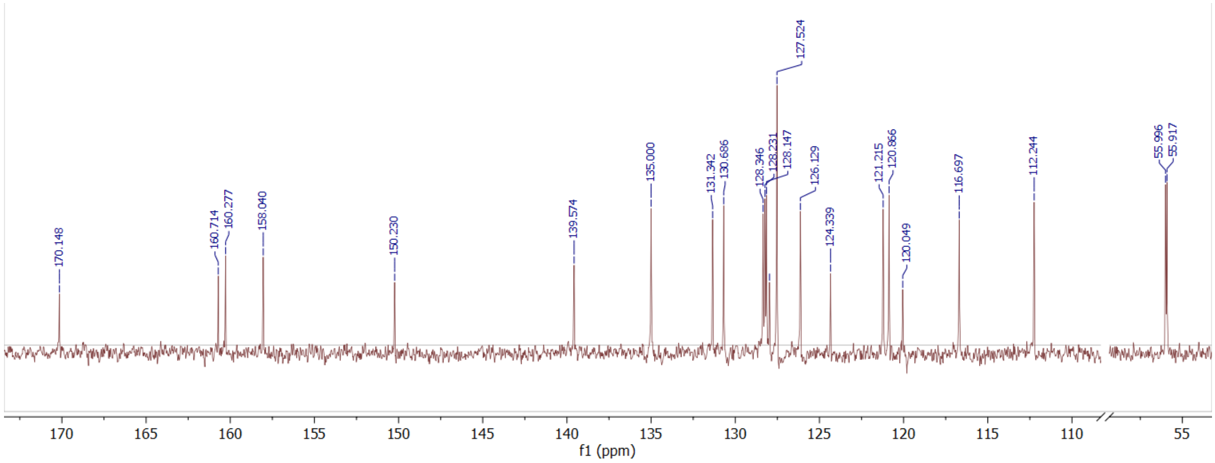

Fig. S32 ^{13}C NMR plot of **IS13**.

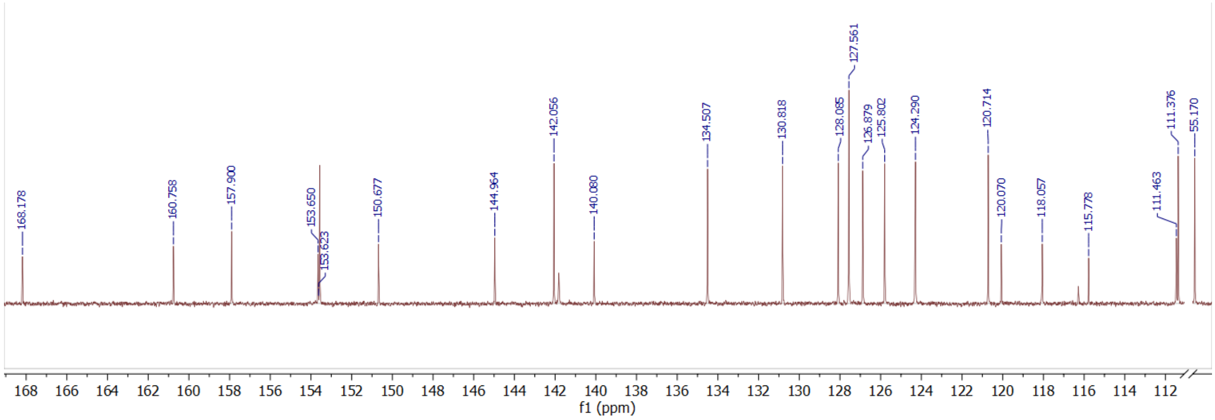

Fig. S33 ^{13}C NMR plot of **IS14**.

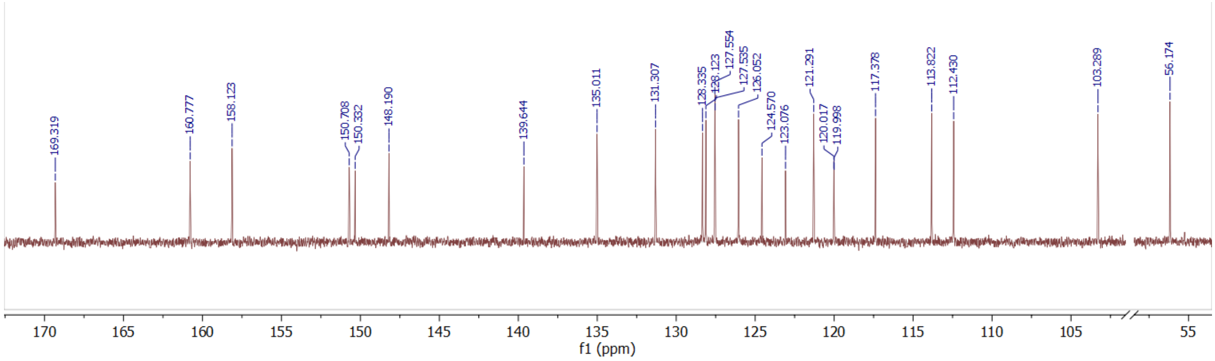

Fig. S34 ^{13}C NMR plot of **IS15**.

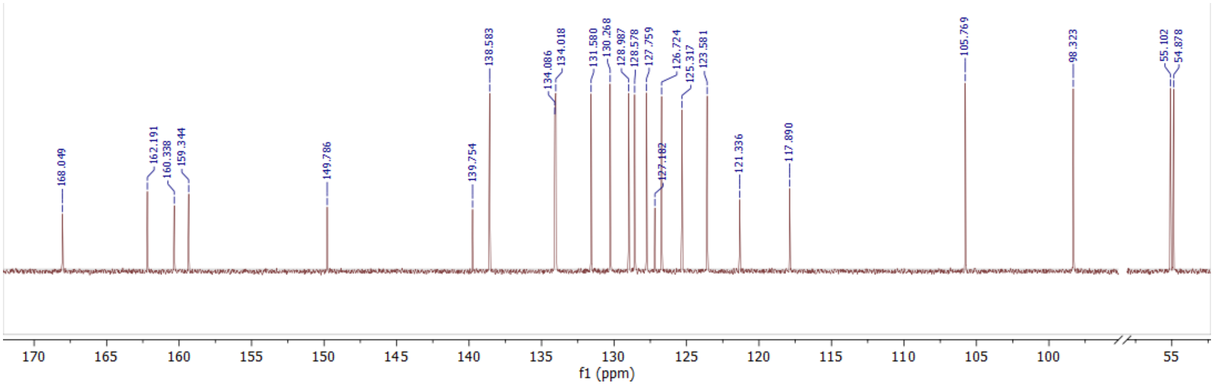

Fig. S35 ^{13}C NMR plot of **IS16**.

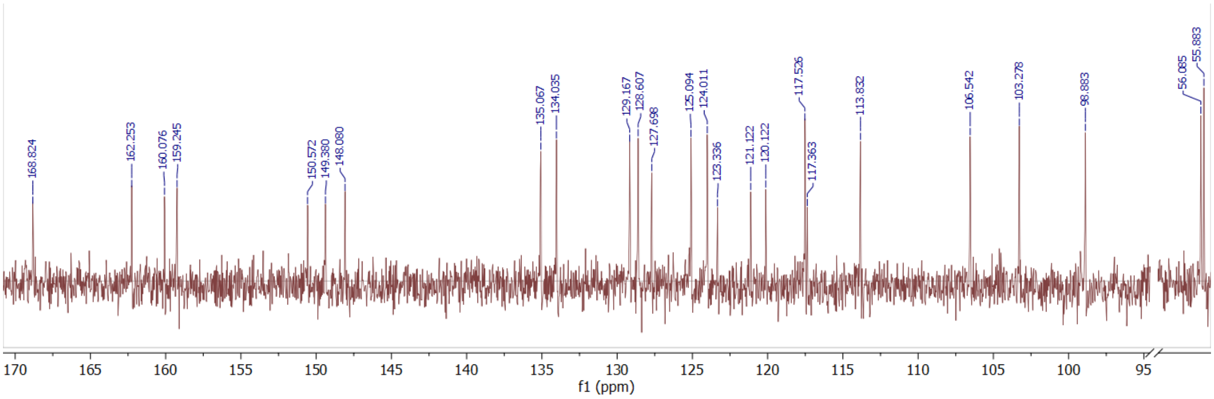

Fig. S36 ^{13}C NMR plot of **IS17**.

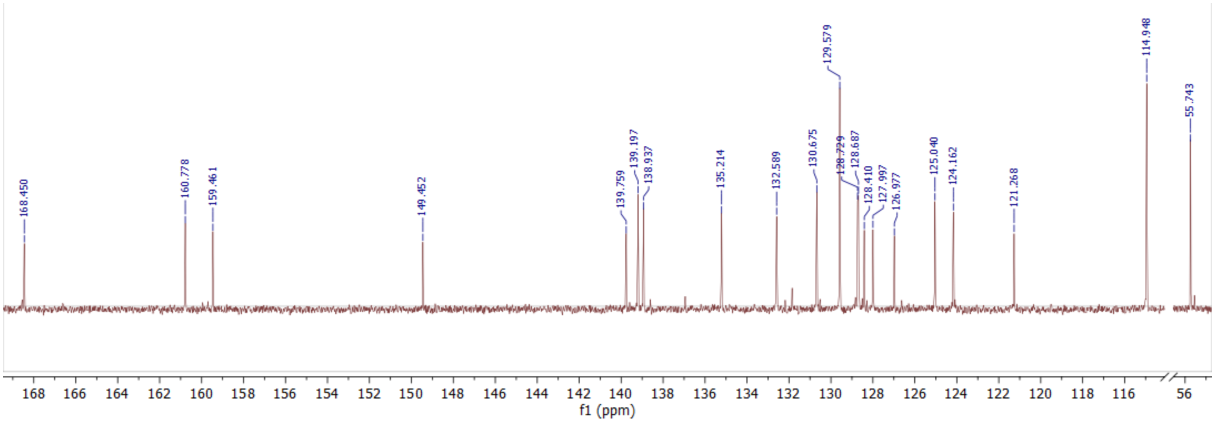

Fig. S37 ^{13}C NMR plot of **IS18**.

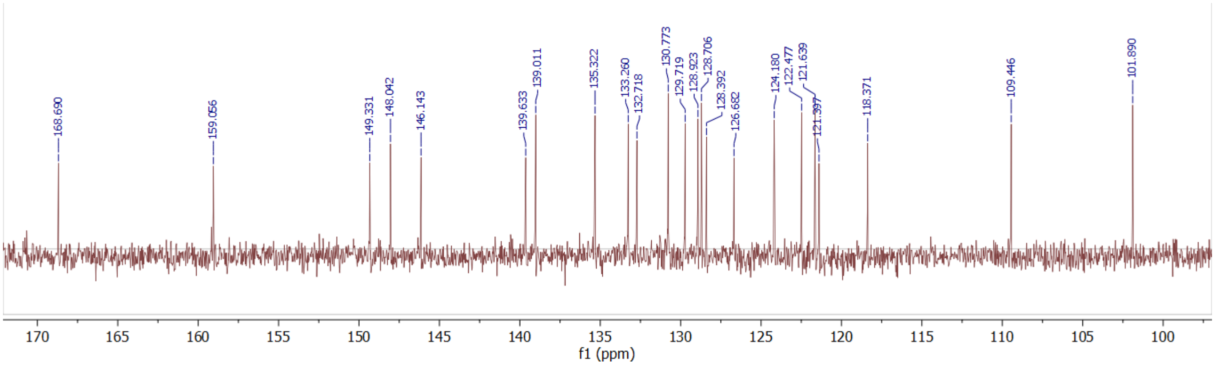

Fig. S38 ^{13}C NMR plot of **IS19**.

1.3.High resolution mass spectra of target compounds

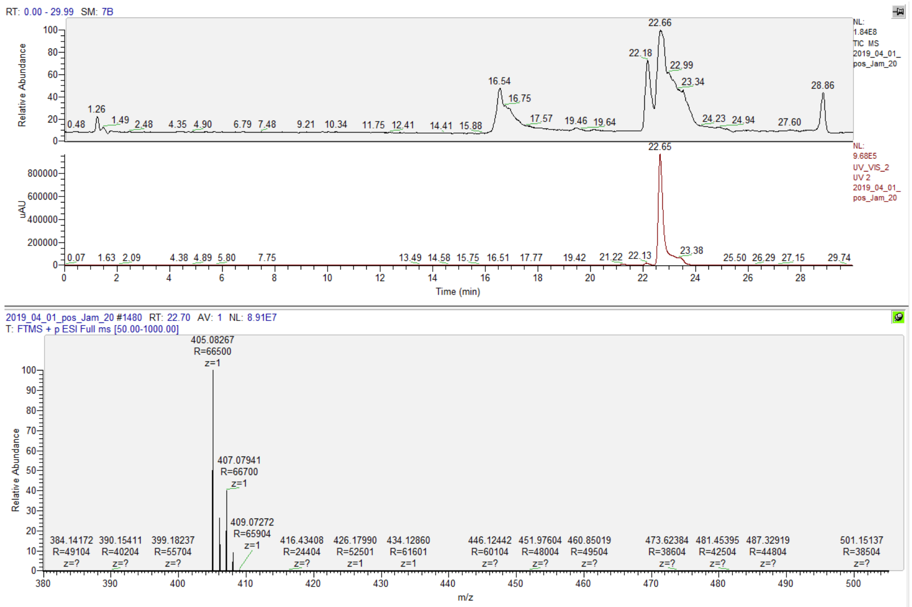

Fig. S39 HRMS spectrum of **IS1**.

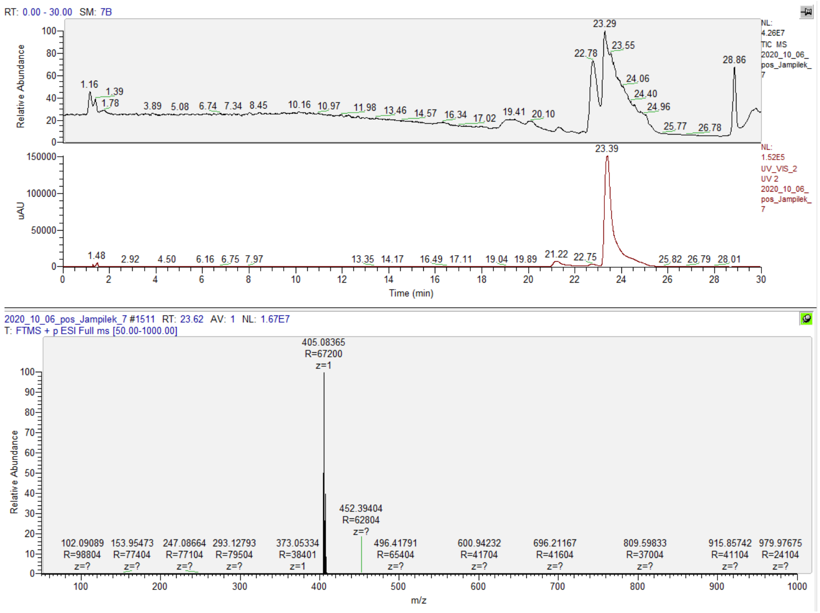

Fig. S40 HRMS spectrum of **IS2**.

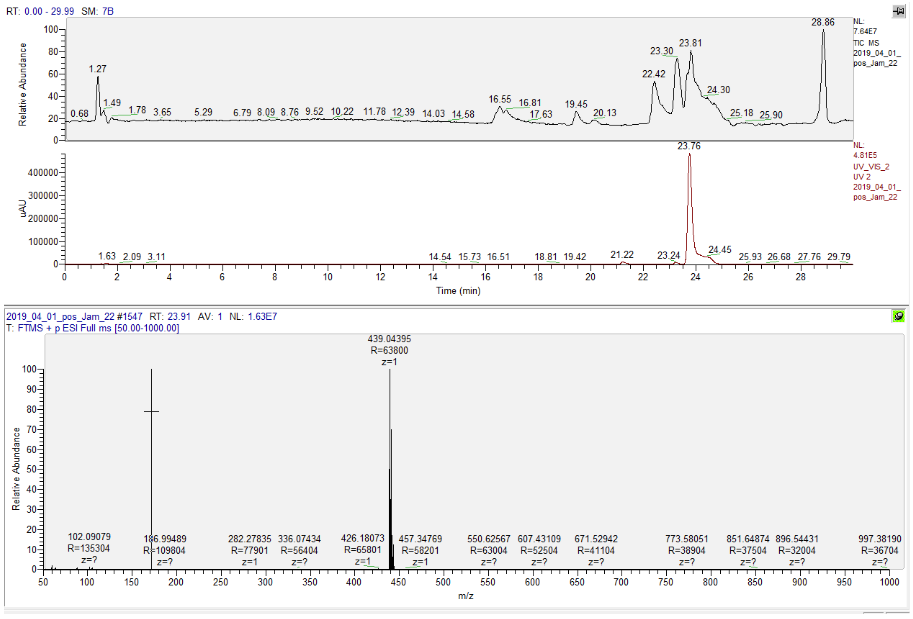

Fig. S41 HRMS spectrum of **IS3**.

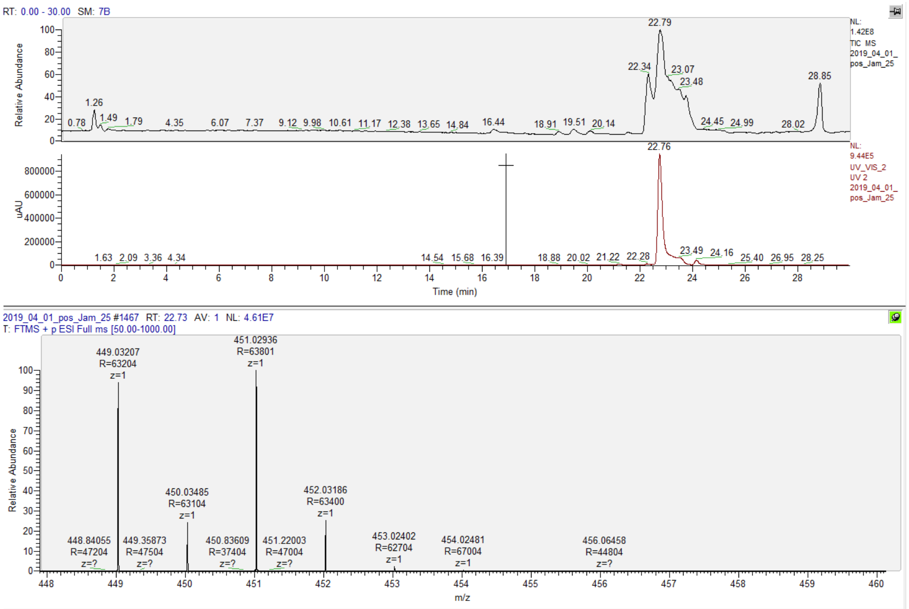

Fig. S42 HRMS spectrum of **IS4**.

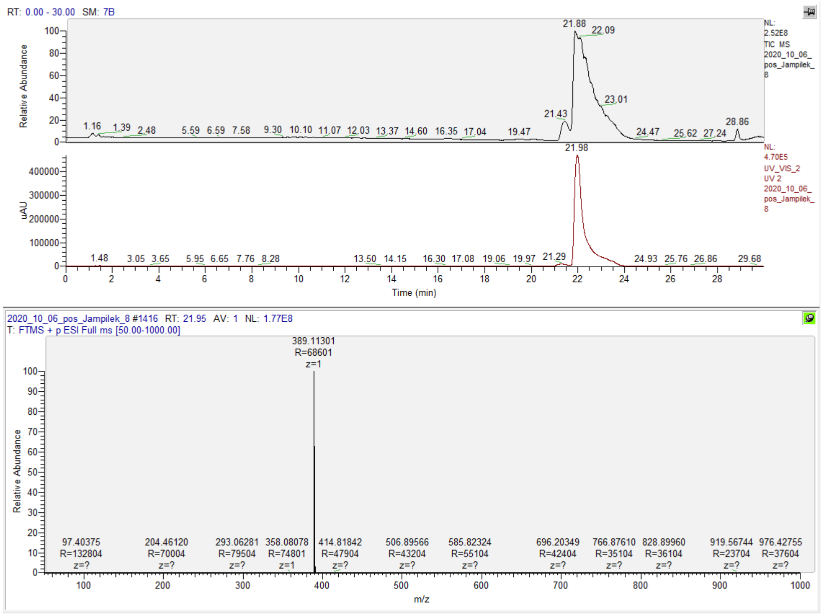

Fig. S43 HRMS spectrum of **IS5**.

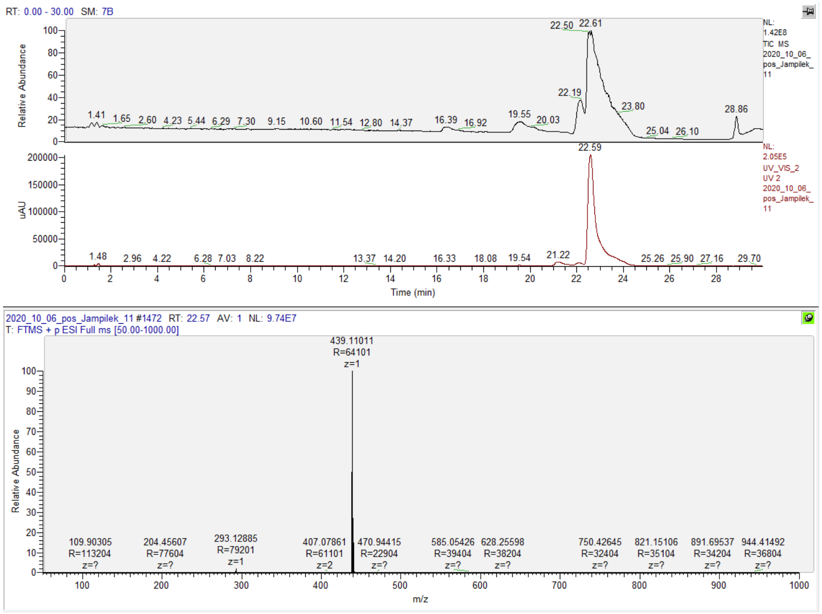

Fig. S44 HRMS spectrum of **IS6**.

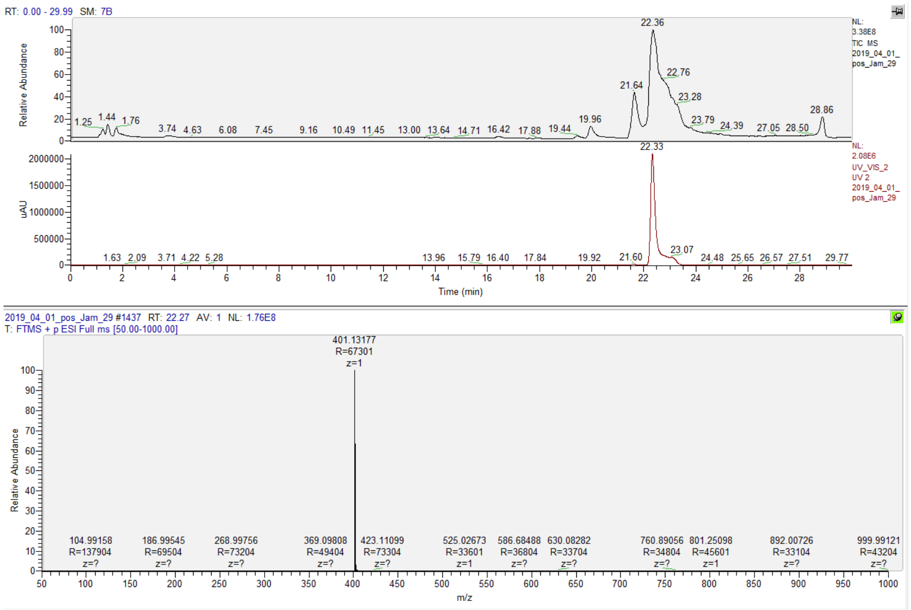

Fig. S45 HRMS spectrum of **IS7**.

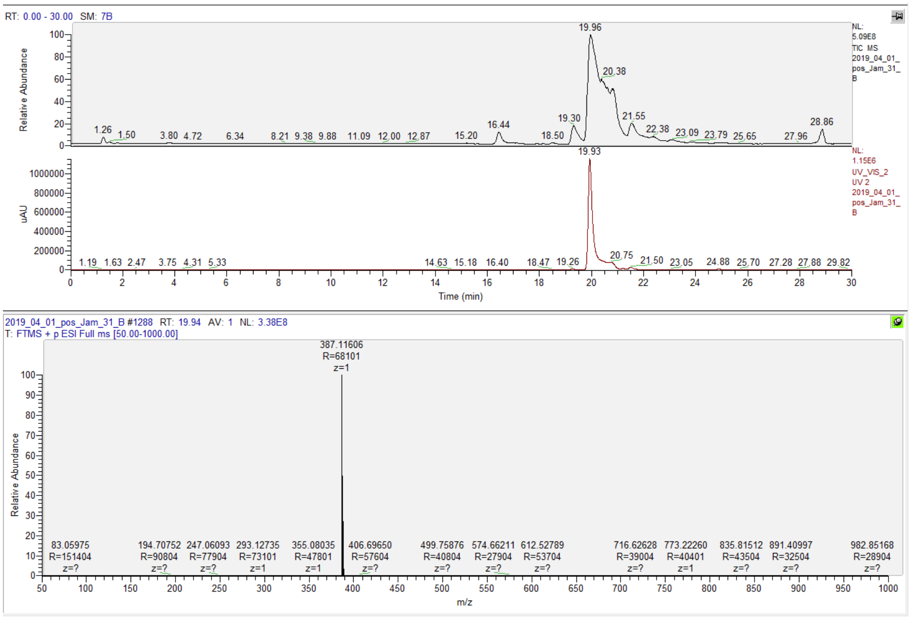

Fig. S46 HRMS spectrum of **IS8**.

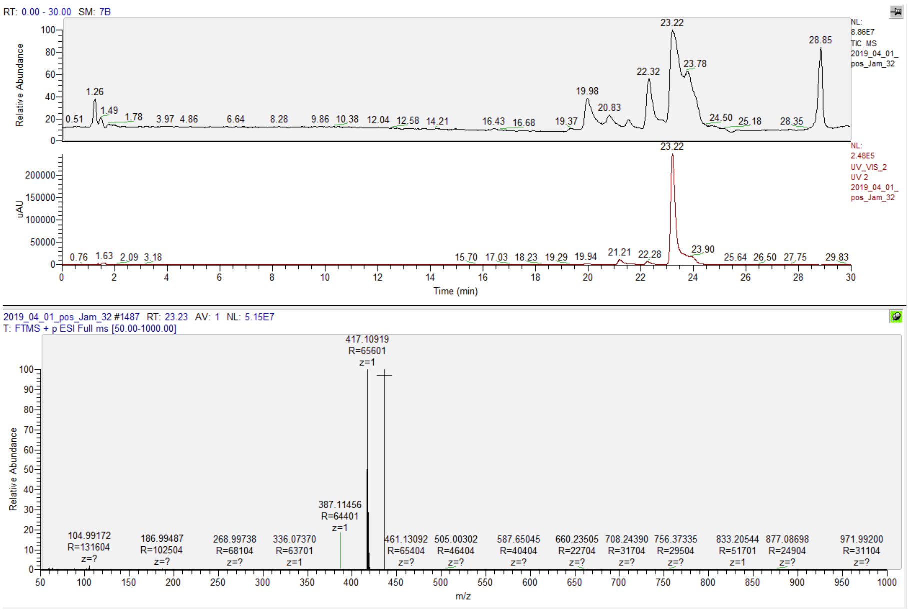

Fig. S47 HRMS spectrum of **IS9**.

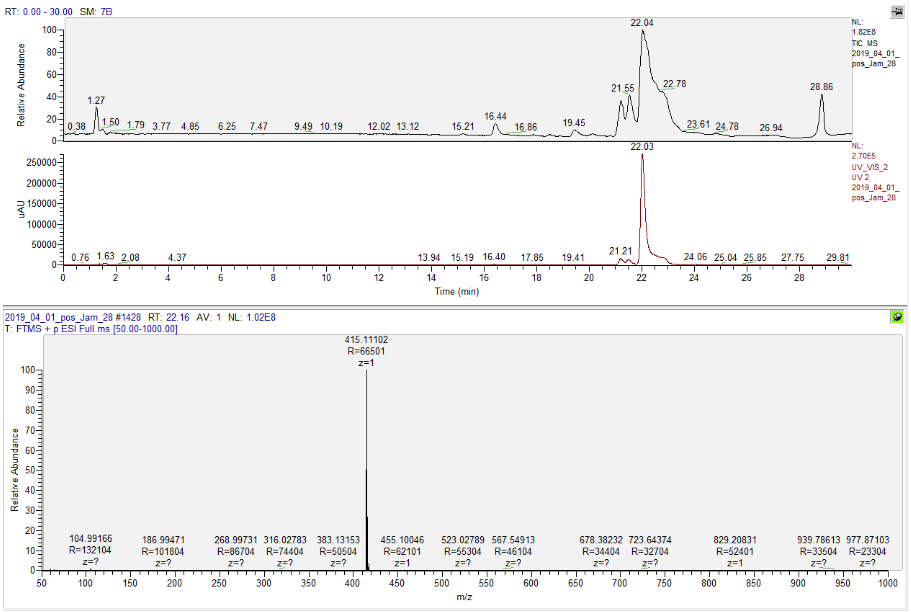

Fig. S48 HRMS spectrum of **IS10**.

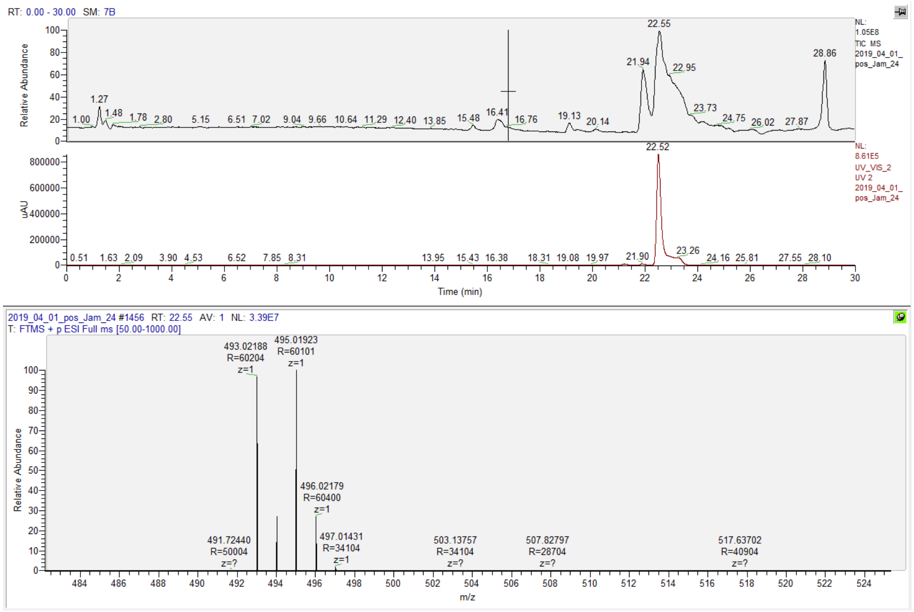

Fig. S49 HRMS spectrum of **IS11**.

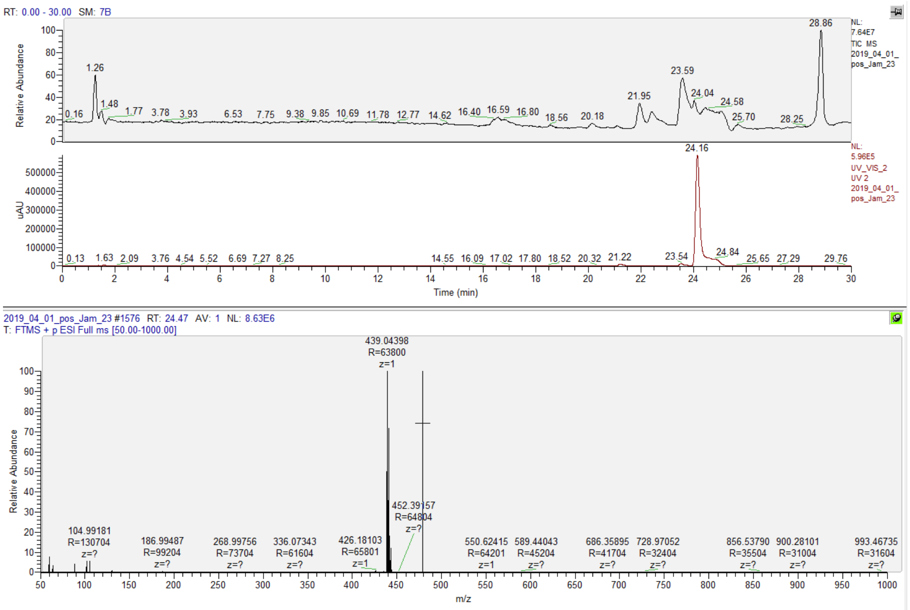

Fig. S50 HRMS spectrum of **IS12**.

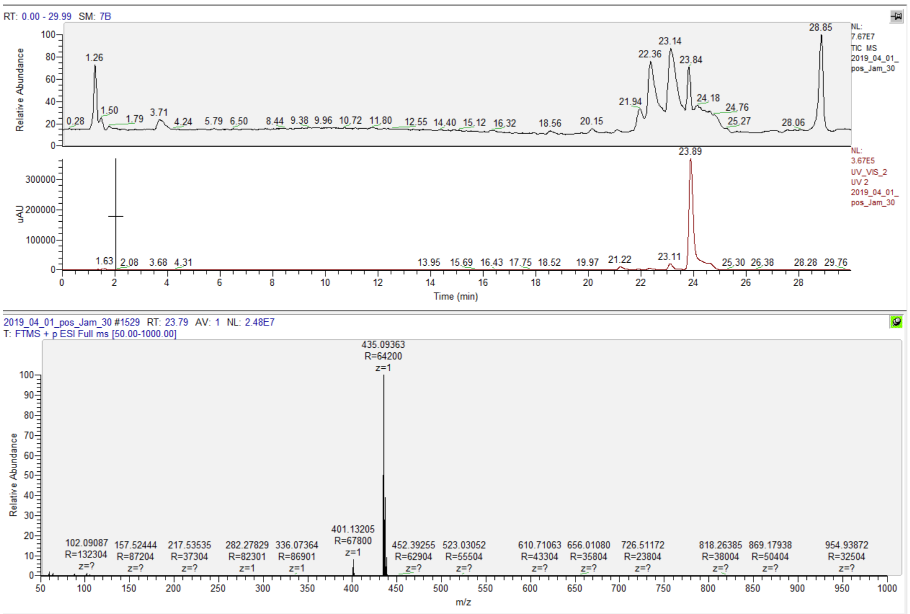

Fig. S51 HRMS spectrum of **IS13**.

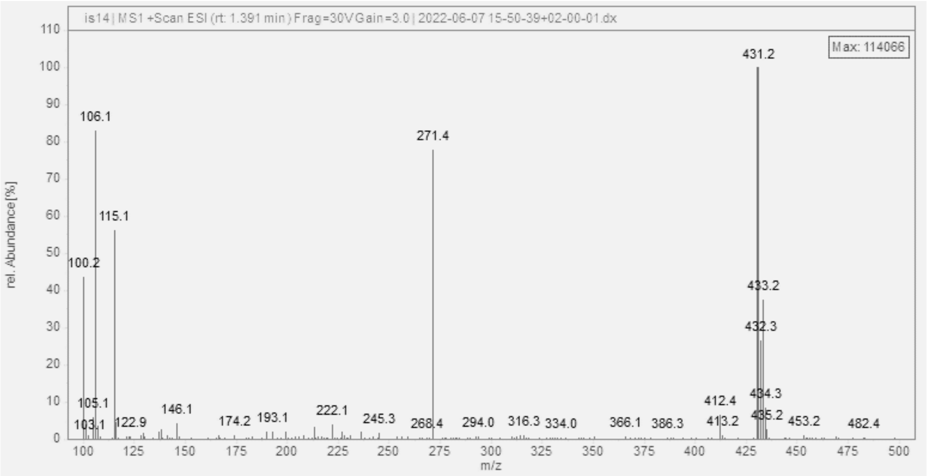

Fig. S52 HRMS spectrum of **IS14**.

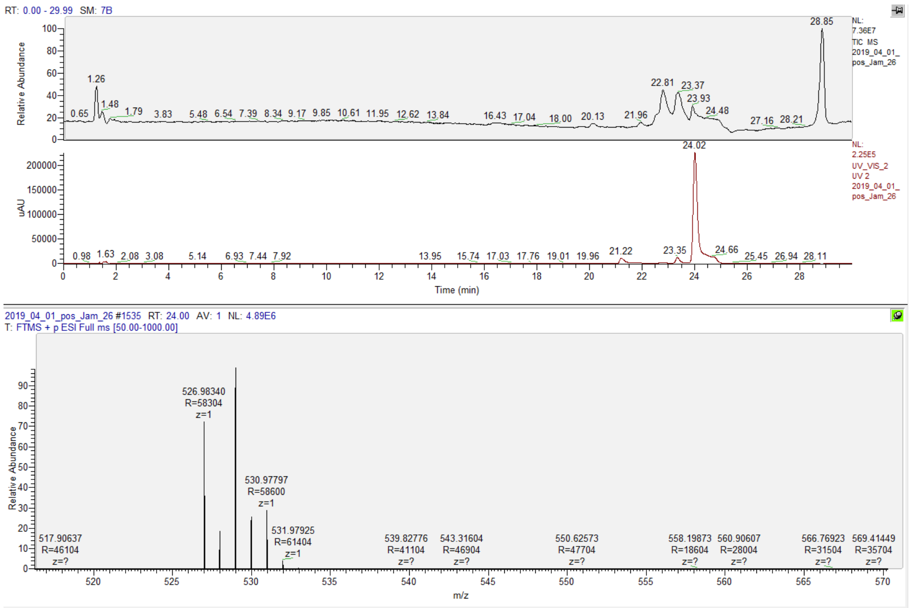

Fig. S53 HRMS spectrum of **IS15**.

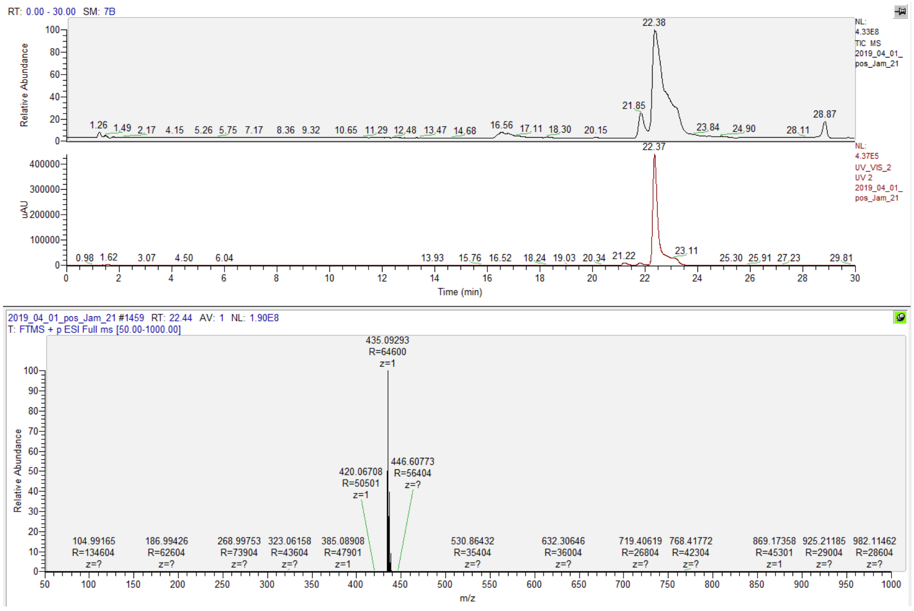

Fig. S54 HRMS spectrum of **IS16**.

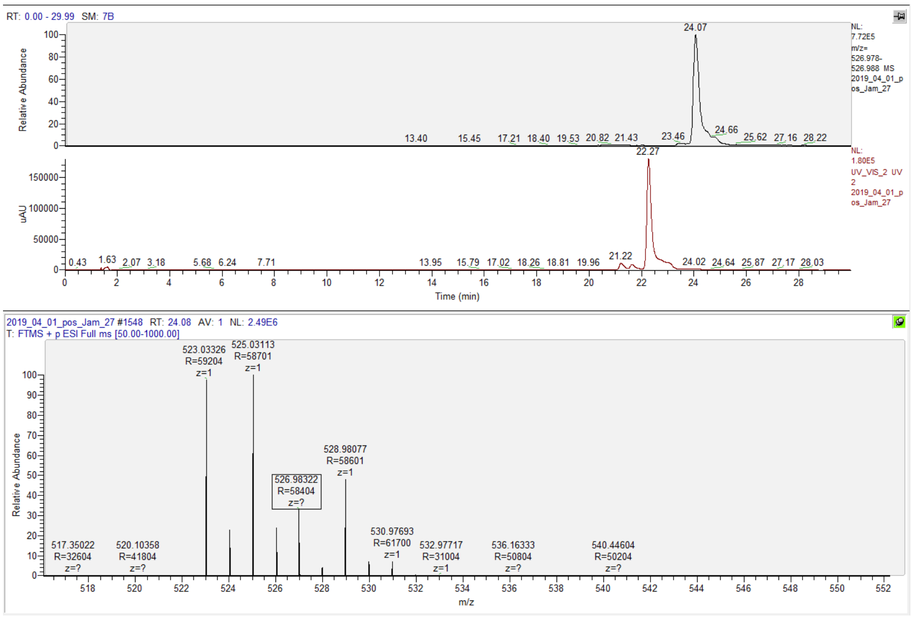

Fig. S55 HRMS spectrum of **IS17**.

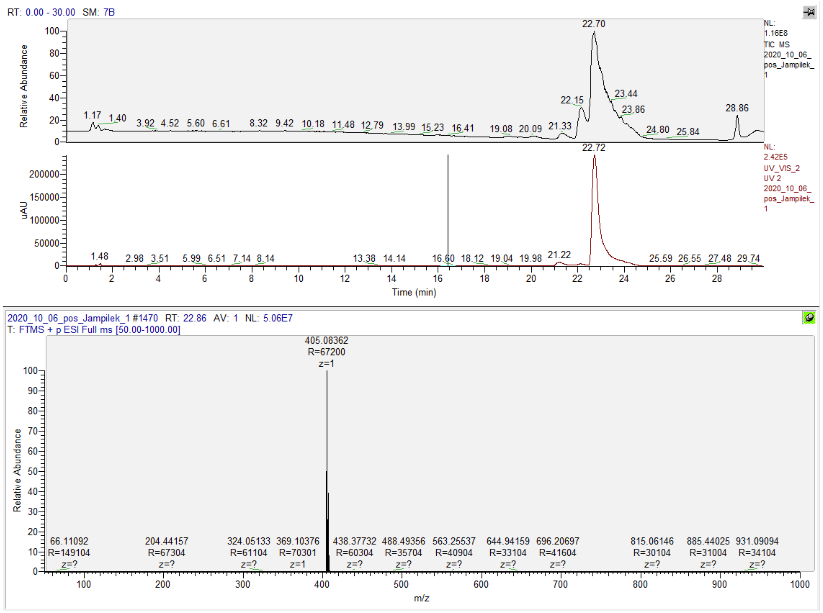

Fig. S56 HRMS spectrum of **IS18**.

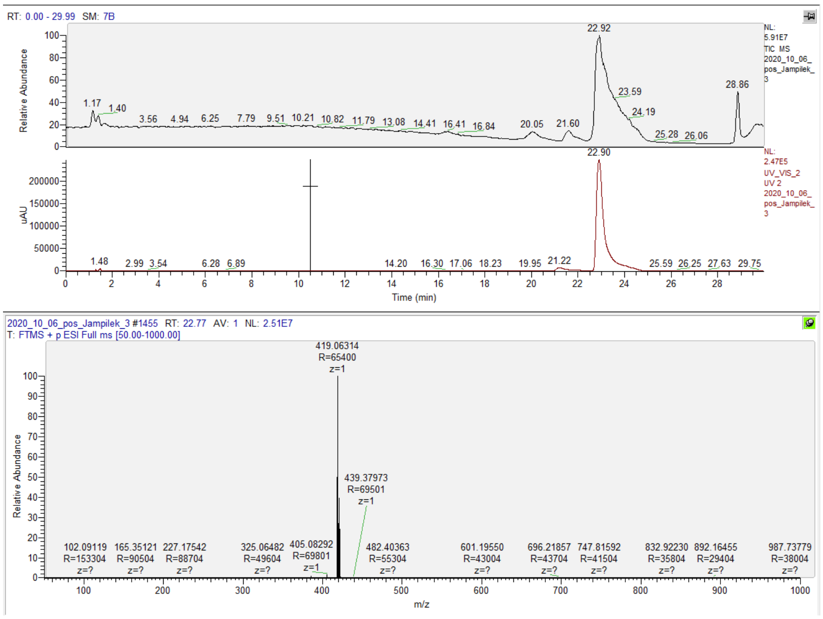

Fig. S57 HRMS spectrum of **IS19**.

2. Biological studies

Table S1. The selectivity indexes of active styrylquinazoline derivatives.

	K562	PANC-1	HCT 116 p53$^{+/+}$	HCT 116 p53$^{-/-}$	MCF-7	A549	U-251
IS1	3.89	1.00	1.00	1.00	1.35	1.00	1.00
IS8	22.98	1.40	1.00	1.00	3.11	1.00	1.00
IS10	12.24	1.00	2.33	2.65	1.54	1.00	1.00
IS16	3.20	-	1.67	-	1.00	1.86	1.20
CP-31398	8.10	1.00	1.34	0.95	0.93	1.00	1.33
Imatinib	187.97	1.00	0.56	0.49	1.00	1.00	1.00
GNF-2	120.19	1.00	1.00	-	1.00	1.00	1.00

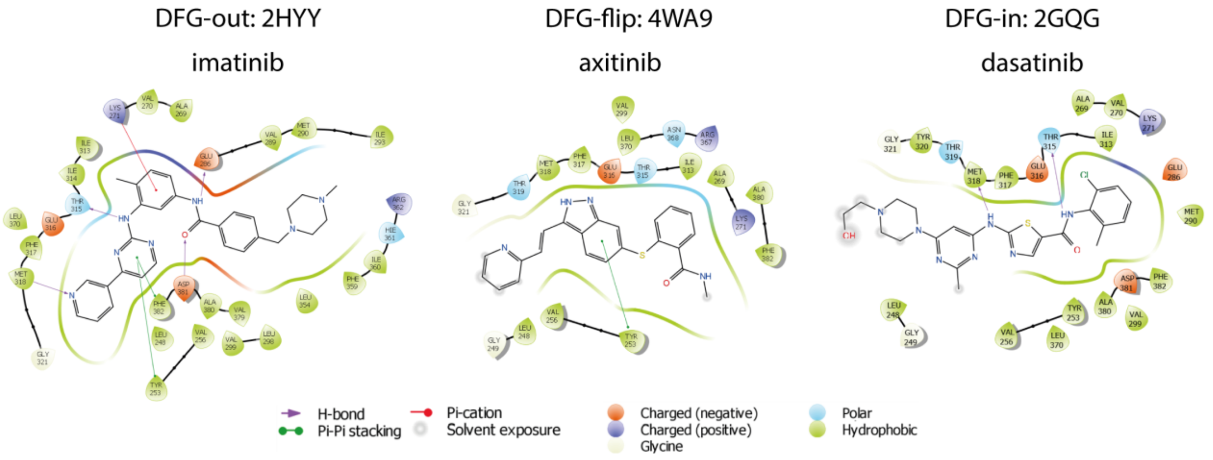

Fig. S58 The 2D diagrams with interactions between ABL inhibitors and ABL kinase in conformational states: DFG-out (2HYY), DFG-flip (4WAW9) and DFG-in (2GQG). The 2D diagrams were generated using Schrodinger Maestro 12 Software.

Table S2. Sequences of primer pairs used in determining the mRNA expression of tested genes.

Gene	Forward primer (5'→3')	Reverse primer (3'→5')
ABL	GAAGCCGCTCGTTGGAACT	CCTAAGACCCGGAGCTTTTCA
SRC	CCTCGTGCGAGAAAGTGAG	TGGCGTTGTCGAAGTCAG
CCNE2	TAGCTGGTCTGGCGAGGTT	GGTTCCAAGTCAGAATGCAGAA
HPRT1	CAGCCCTGGCGTCGTGATTAGT	CCAGCAGGTCAGCAAAGAAT
